# Digitizing Chemical Synthesis in 3D Printed Reactionware

**DOI:** 10.1002/anie.202116108

**Published:** 2022-03-25

**Authors:** Andrius Bubliauskas, Daniel J. Blair, Henry Powell‐Davies, Philip J. Kitson, Martin D. Burke, Leroy Cronin

**Affiliations:** ^1^ School of Chemistry The University of Glasgow Glasgow G12 8QQ UK; ^2^ Roger Adams Laboratory, School of Chemical Sciences University of Illinois Urbana-Champaign IL 61801 USA

**Keywords:** 3D Printing, C−C Coupling, Chemical Education, Reactionware, Unit Operations

## Abstract

Chemistry digitization requires an unambiguous link between experiments and the code used to generate the experimental conditions and outcomes, yet this process is not standardized, limiting the portability of any chemical code. What is needed is a universal approach to aid this process using a well‐defined standard that is composed of syntheses that are employed in modular hardware. Herein we present a new approach to the digitization of organic synthesis that combines process chemistry principles with 3D printed reactionware. This approach outlines the process for transforming unit operations into digitized hardware and well‐defined instructions that ensure effective synthesis. To demonstrate this, we outline the process for digitizing 3 MIDA boronate building blocks, an ester hydrolysis, a Wittig olefination, a Suzuki–Miyaura coupling reaction, and synthesis of the drug sulfanilamide.

## Graphical abstract

A new approach to the digitization of organic synthesis that combines reactions as building blocks with 3D printed reactor hardware or reactionware is presented. This practical approach allows the synthetic chemist to encode the unit operations allowing a digital blueprint to be built of the entire process.

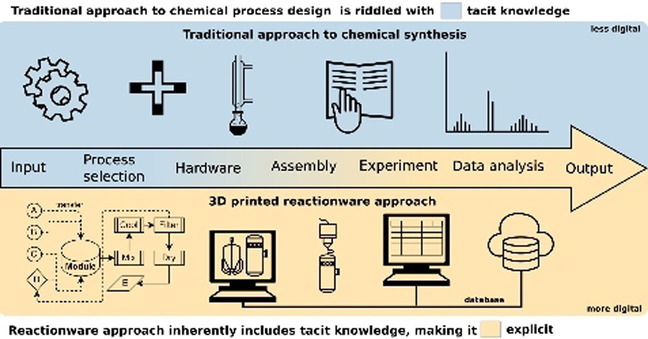

## Introduction

**Figure 1:**
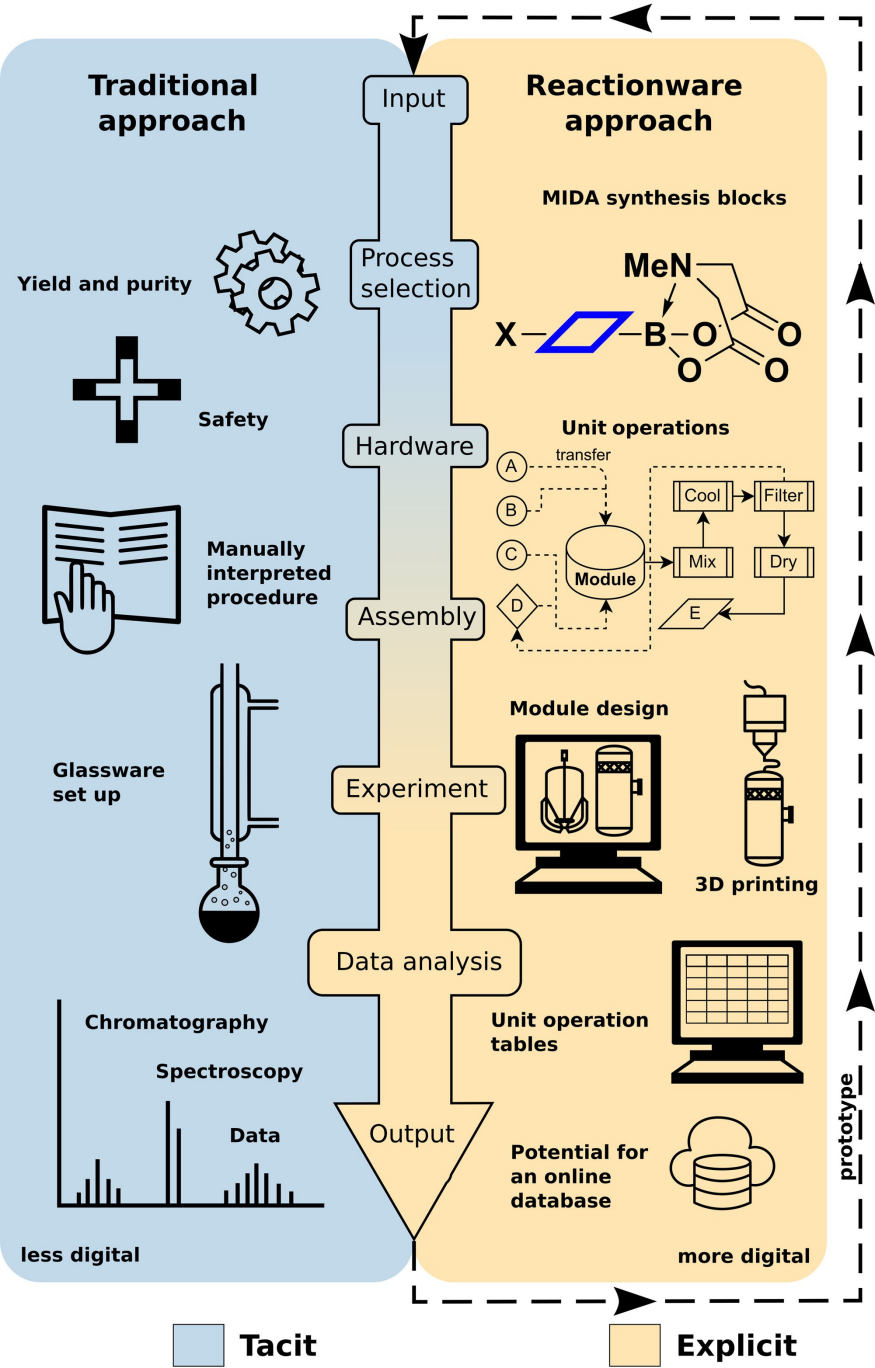
Traditional approach (left) and reactionware approach (right) to synthesis. In contrast to the traditional approach, a reactionware chemist will consider additional aspects on top of what is considered normally. All aspects in the traditional approach are inherently included in the reactionware approach when a chemical reaction is performed and recorded. The reactionware protocols are explicit, inherently including many process parameters, whereas the traditional approach is tacit in that a lot of considerations that were made by the chemist are lost in traditionally‐recorded protocols.

Automated chemical synthesis empowers non‐experts to access molecular function by capturing accumulated expertise within user‐friendly self‐contained devices.^[1]^ The impact of generalized automation has already been substantial in the areas of peptide,^[2]^ oligonucleotide,^[3]^ and oligosaccharide synthesis^[4]^ where commercial synthesizers are now routinely employed.^[5]^ Critically, the limited range of chemical reactions required to assemble the modular building blocks for these biopolymers has facilitated their capture within an automated regime.^[6]^ The digitization of chemical synthesis requires all the relevant parameters and processes to be accurately captured in a modular way.^[7]^ To achieve this digital transformation requires thinking about chemical synthesis at a different level of granularity and to be as precise and unambiguous as possible (Figure 1).^[8]^ For example, recording the number of reaction steps, reaction time and basic reagent information is not sufficient to encode a synthetic sequence. This is because reduction of chemical synthesis into practice requires a tremendous amount of tacit knowledge relating to specific reactions, reagents and chemotypes, limiting reproducibility.^[9]^ So, for reactions to be successfully digitized all unit operations (heating, stirring, transfer, etc.) need to be identified and accounted for. Defining and grouping unit operations is a common principle in process chemistry where every detail is captured, leading to fewer difficulties during automated synthesis platform development. By accounting for unit operations, the missing link of knowledge is made explicit in the design of chemical syntheses using reactionware^[10]^ ‐ a series of modular polypropylene 3D printed reactors dedicated for multi‐step synthesis, batch, or flow. In chemistry, 3D printing has found expansive applications in both education^[11]^ and synthesis.^[12]^ Reactionware, specifically, has been used in synthesis of drug molecules^[13]^ and on‐demand chemicals such as polyoxometalates, organic catalysts and protein labeler molecules,^[14]^ and recently for electrochemistry.^[15]^

Reactionware necessitates a more granular, process‐oriented mode of thinking as the chemical process is dissected into discrete individual unit operations which allows them to be grouped and assigned as sets of unit operations to modules. This brings a modular way of thinking about a chemical process to the chemist. In turn, the process is accounted for effectively, whilst keeping tight control over individual unit operations and their respective parameters, ultimately aiding reproducibility.^[16]^

Herein we demonstrate the application of this approach to a number of generic example syntheses of widely used organic transformations; an ester hydrolysis; a Wittig olefination; a Suzuki–Miyaura coupling reaction; and synthesis of the drug sulfanilamide. We also extend this approach to the design of systems which facilitate the use of modular chemistry to access large areas of synthetic space.

Progress has been made toward the generalized automated synthesis of small organic molecules through modular synthesis approaches such as the iterative assembly of *N*‐methyliminodiacetic acid (MIDA) boronate building blocks.^[17]^ While MIDA based synthesis platforms have limitations, they can access a wide range of chemical space and the technology continues to advance leading to increasingly expanded reach of chemical space.^[18]^ However, unlike peptides the requisite building blocks cannot be obtained from natural sources and are therefore not yet as widely commercially available. Thus, the power of chemical synthesis is still limited to those who have access to laboratories and relevant expertise. The modular nature of MIDA boronate chemistry can be combined with reactionware and has the potential to be applied in digital synthesis platforms holistically. In this regard we envisage that the digitization of chemical synthesis stands to bridge the divide between automated synthesis, accessible building blocks, and modular reaction methodologies.

The practical approach that the reactionware chemist takes towards chemical synthesis leads to easy access to building blocks. MIDA boronate chemistry can be defined using process chemistry principals and combined with reactionware to encapsulate several chemical syntheses that are important today. One important application of this approach is to seamlessly marry Lego‐like synthesis[^12a^, ^13^] and reactionware[^17^, ^19^] to democratize the molecule making process. This approach combines substrates for modular reactions with reactionware and can be prototyped rapidly in a reproducible manner. Further, this approach paves the way to innovation and collaboration through digital means and encompasses many fundamental aspects that require consideration when developing digital synthesis platforms: 1) total account of unit operations involved in the process and their application in reactors in the form of functional modules; 2) reactor design and the relationship between its geometry, module functionality and the chemical process at hand; 3) the format and content of a machine‐readable instructions. Many aspects of chemical synthesis are digitized when using reactionware. Digital reactor design files and explicit synthesis procedures are accompanied with basic synthesis data (NMRs, yields, purities, etc.) and can be shared globally. A key future goal will be to make unit operation tables machine readable to fully digitize the process. To facilitate the more widespread adoption of this strategy we provide two manuals dedicated for students/teachers, describing how reactionware can be applied in the context of chemical education. Presented reactions were selected based on common synthesis experiments that appear in undergraduate teaching syllabuses today.

## Results and Discussion

### 3D Printed Reaction Vessels “Reactionware”

Reactionware are composed of a series of modules with dedicated functionality that exhibit various structural features which are defined in code. The order in which these modules can be joined, and their features depend entirely on the chemical process and scale of reaction. Each unit operation (material addition, mixing, heating cooling, mixture separation, product isolation and purification, etc.) and unit operation parameters (reaction times, temperatures, volumes etc. as constraining factors) necessary to carry out the synthesis are considered for each module system individually. The type of chemical process dictates the type of module which can be generated using the open‐source *ChemSCAD* software.^[19]^ The various module types accommodate specific groupings of unit operations for example: Reactor (mixing, heating, cooling, evaporation), Filter Reactor (mostly product isolation or purification), Flow Reactor (flow chemistry) and Floating Filter Reactor (phase separations) among others.

Other considerations revolve around the physical state of compounds used during synthesis: adding liquids require an external inlet to be positioned either on the top or side of the 3D printed cylinder to avoid pressure build up inside the module. In contrast, powder addition requires larger inputs which can be threaded to fit screw caps. All modules can be inter‐connected via hollow connections between modules, or modules can be printed individually and connected via PTFE or other chemically resistant tubing. Polypropylene or other material fittings could be mounted easily with the correct tools and print post‐processing. Temperature control in reactionware cartridges can be achieved by heating with microwaves (polypropylene microwave absorption is negligible)^[20]^ or in traditional oil/sand baths with cooling achieved in ice/slurry baths. Polypropylene, being an insulator, requires reactionware to be heated to a higher temperature than is required in glassware. Moreover, polypropylene starts becoming malleable at around 140 °C, thus only lower temperature requiring reactions can be performed in reactionware. Reactionware systems can be sealed and kept under an inert atmosphere during an entire multi‐step procedure, or open to the environment, provided the chemical system is unaffected by atmospheric factors. Reaction intermediates can easily be isolated in individual modules, impurities controlled by imbedded glass frits or other filters. Crystallization and evaporation under reduced pressure can also be performed within reactionware modules. All reactionware‐associated files that were used in the syntheses described below are uploaded along with the Supporting Information.

To demonstrate the reduction of chemical synthesis to an entirely digitized format we present a series of syntheses that illustrate different challenges that arise during the digitization of chemical transformations using reactionware. We outline the elementary steps and considerations for composing operational reactionware cartridges across a series of chemical transformations of increasing complexity. A full list of materials, chemicals, detailed experiment procedure with 3D‐printing settings and instructions with user notes can be found in the Supporting Information. All procedures were performed in glassware, then in reactionware. Thus, Supporting Information manuals can be used as glassware/reactionware experiment templates.

In the Supporting Information, there are two sections. Section A describes individual glassware and reactionware experiments in detail and is accompanied with all the associated data. Section B, however, has been tailored for adoption in teaching laboratories as lab manuals for experiments in reactionware. This section contains, for each experiment: 1) an introduction that describes the chemistry in detail, 2) pre‐lab questions, 3) experimental procedure and, finally, 4) post‐lab questions that would help students understand the chemistry at hand.

### MIDA Boronate Ester Synthesis

Methyliminodiacetic acid (MIDA) boronates represent bench stable modular building blocks that are compatible with iterative automated synthesis^[17a]^ of increasing generality.^[18]^ However, despite their utility, many boronic acids are not yet commercially available in a stable MIDA boronate form. We thus focused on digitizing the recently reported method for the direct generation of MIDA boronates from boronic acids.^[17d]^ This reaction involves the condensation of a boronic acid with a dehydrated form of MIDA, dubbed MIDA anhydride. The reactionware monoliths (modules systems) were designed using *ChemSCAD*, a user‐friendly software dedicated specifically for producing reactionware 3D‐models.^[19]^ The models were printed using Ultimaker 2+3D printers and using Cura open‐source slicing software to prepare the digital gcode file that is read by the printer. During printing, a pause was pre‐programmed so that a glass filter frit could be inserted for the two modules Printer setting file (.curasettings, 3D models (.stl) and printer instruction (.gcode) files are available to download. Reactionware model specifications for recreating the models in *ChemSCAD* can be found in the Supporting Information (page S7) along with further details on 3D print post‐processing, part supplier lists, and other instructions.

**Figure 2:**
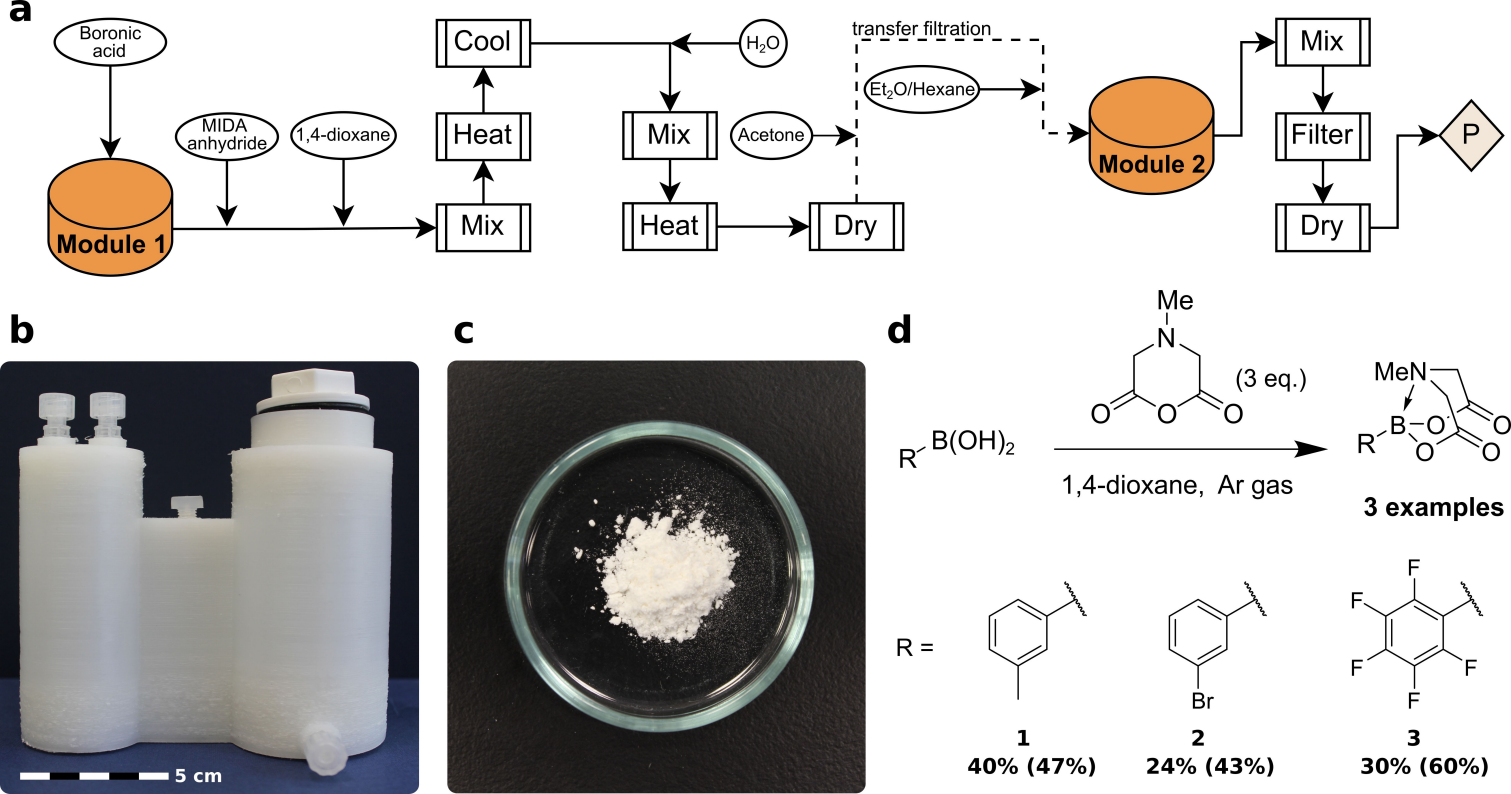
a) Process flow diagram displaying unit operations and their sequence (P: product), b) 3D‐printed reactionware as designed *ChemSCAD*, c) compound **3** product powder, d) reaction scheme for MIDA boronate ester synthesis (%Yield of compound prepared in reactionware is in bold with %Yield as prepared in glassware is in parentheses).

In this reactionware (Figure 2b), first, arylboronic acid_(s)_ and the MIDA anhydride_(s)_ are dissolved in anhydrous 1,4‐dioxane under inert atmosphere with heating. Water is added to quench excess MIDA anhydride converting it into the acetone insoluble diacid MIDA. The dioxane is evaporated under reduced pressure and mild heating at 70 °C. This is achieved in a heating bath, with a vacuum line attached to the port of the module. Then acetone is added, solubilizing the product and leaving solid impurities suspended in solution. A transfer line allows filtration of the acetone solution into a second reactionware module. The addition of diethyl ether and hexane to the acetone solution causes precipitation of the MIDA boronate which is isolated by filtration through opening a bottom outlet valve to provide the solid MIDA boronate. In total 15 unit operations capture the process of converting a boronic acid into a MIDA boronate using reactionware. Across 3 different boronic acids practical amounts of MIDA boronate product were obtained m‐tolyl **1** (104 mg), 3‐bromophenyl **2** (75 mg), and pentafluoro **3** (97 mg). This procedure was challenging in that it required an inert atmosphere within reactionware. Inert atmosphere was achieved by flushing reactionware with argon gas and equipping an argon balloon attached to a syringe via one of the Luer lock ports on the first reactionware module.

### Ester Hydrolysis

**Figure 3:**
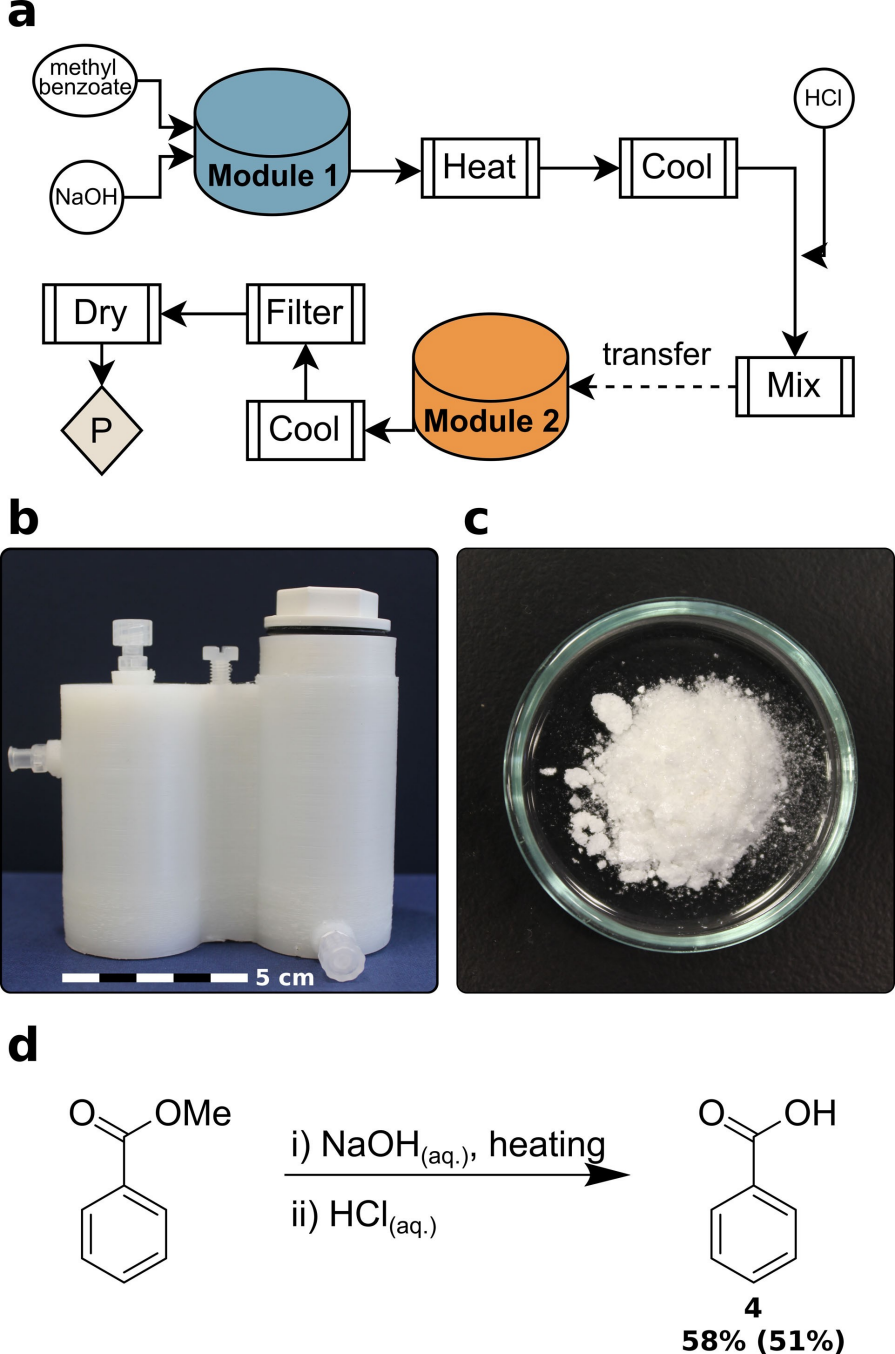
a) Process flow diagram displaying unit operations and their sequence for ester hydrolysis (P: product), b) 3D‐printed reactionware as designed using *ChemSCAD*, c) carboxylic acid product powder, d) reaction scheme for ester hydrolysis (%Yield of compound prepared in reactionware is in bold with %Yield as prepared in glassware is in parentheses).

In medicinal chemistry, ester hydrolysis is a widely used functional group interconversion (FGI) to obtain carboxylic acids.^[21]^ Thus, this FGI was chosen to be performed in a 2‐module reactionware (Figure 3b) as designed using *ChemSCAD*. The first 50 mL module is a “Reactor” module type and the second is a 50 mL “Filter Reactor”. Full module specifications can be found in the Supporting Information. The chemical transformation starts with NaOH_(aq.)_ is added to an ethanol solution of the ester methyl benzoate and then heated to reflux via submerging reactionware into an oil bath and mounting a 3D‐printed high‐surface condenser on the reactor module (Supporting Information, Figure 19b). Under alkaline conditions the sodium salt of the benzoic acid is formed along with MeOH, which is quenched through acidification. Post‐cooling, the mixture is filtered into a second reactionware module and the mixture is cooled causing precipitated of the benzoic acid product. The product was isolated by filtration through opening the bottom outlet valve to provide benzoic acid **4** (230 mg) in a process composed of 10 unit operations. Previously, heating under reflux without solvent loss in reactionware was difficult. This issue was solved by 3D printing a polypropylene high‐surface condenser that enables retaining reaction solvent within reactionware under heating.

### Wittig Olefination

**Figure 4:**
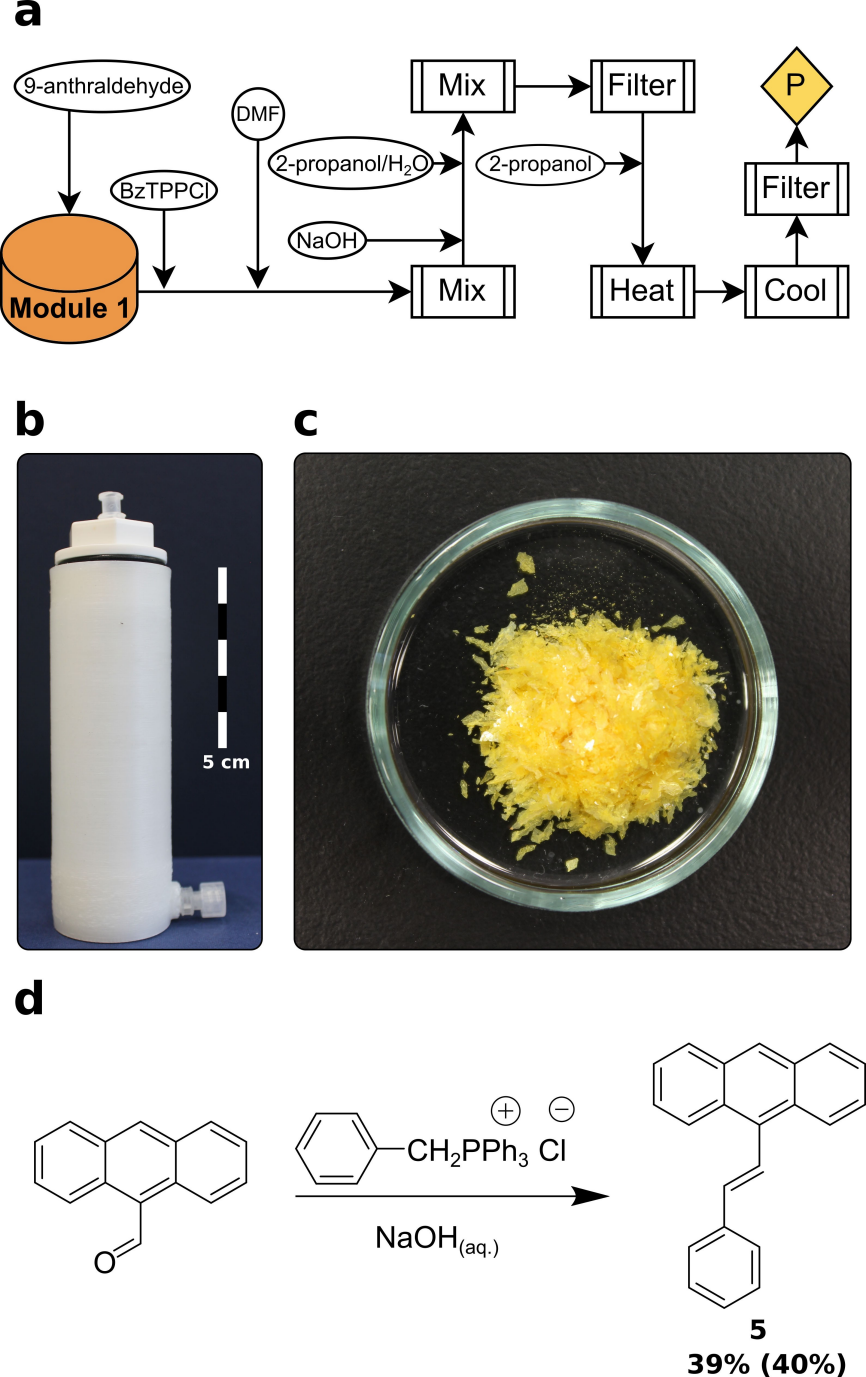
a) Process flow diagram displaying unit operations for Wittig olefination (P: product, BzTPPCl: benzyl triphenylphosphonium chloride), b) 3D‐printed reactionware as designed using *ChemSCAD*, c) alkene product crystals, d) reaction scheme for Wittig olefination (%Yield of compound prepared in reactionware is in bold with %Yield as prepared in glassware is in parentheses).

The Wittig reaction is a common method for transforming an aldehyde group to an alkene.^[22]^ The Wittig reaction is stereoselective and is known for producing the *Z*‐alkene in high selectivity.^[23]^ However the example process selected for reactionware produces the *E*‐alkene instead. The reaction was performed in a single 60 mL “Filter Reactor” module (Figure 4b) that can be recreated using *ChemSCAD* using the details in the Supporting Information. 9‐anthraldehyde_(s)_ and benzyltriphenylphosphonium chloride_(s)_ were added to the reactionware monolith. The Wittig reaction was initiated through addition of DMF and 50 % NaOH and stirred at RT for 1 h. The addition of a 2‐propanol/water mixture caused precipitation, allowing the triphenylphosphine by‐product to be subsequently removed by recrystallizing the crude mixture using 2‐propanol. The target compound was isolated as yellow plates (187 mg). This process is composed of 12 unit operations and uses only a single reactionware monolith to achieve reaction and purification.

### Suzuki–Miyaura Coupling

**Figure 5:**
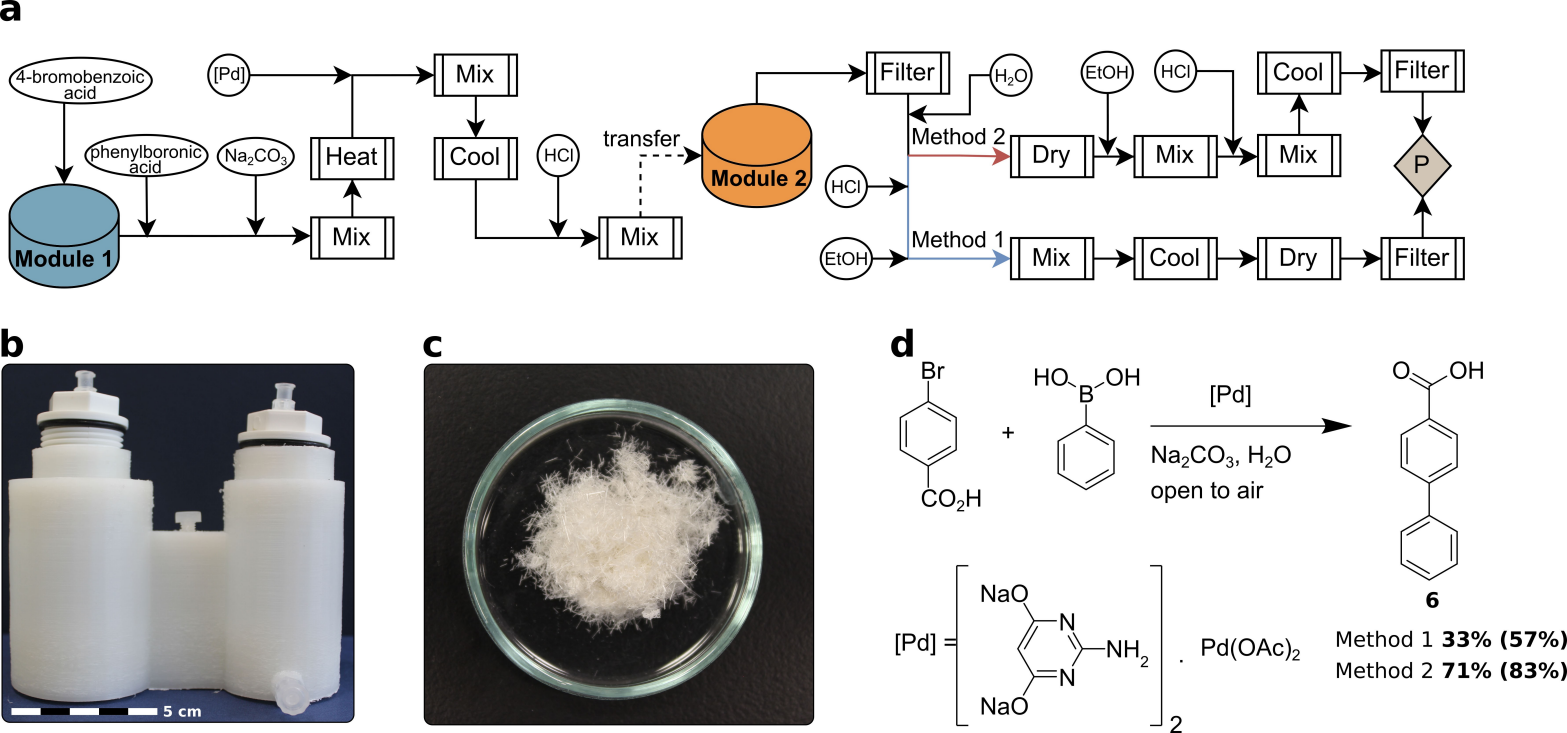
a) process flow diagram displaying unit operations and their sequence for Suzuki–Miyaura coupling (P: product), b) 3D‐printed reactionware as designed using *ChemSCAD*, c) compound **6** product crystals as prepared using Method 1, d) reaction scheme for Suzuki coupling (%Yield of compound prepared in reactionware is in bold with %Yield as prepared in glassware is in parentheses).

C−C bond forming reactions such as the Suzuki–Miyaura are one of the most common transformations in a synthetic lab,^[21]^ thus, a green protocol variation^[24]^ of the Suzuki–Miyaura reaction performed in water was chosen and two methods were developed. Both synthesis methods can be used using the same model of reactionware for which full module specifications can be found in the Supporting Information for easy reproduction using a 3D printer. This process starts by mixing an aryl‐halide with a boronic acid, coupling them together using a pre‐made stock solution of Pd catalyst under basic conditions and heating. Then, acid is added slowly to precipitate the crude product whilst avoiding excessive amounts of gas forming. The final product can be isolated by either recrystallization or using a precipitation with anti‐solvent and filtration approach. We provide two detailed process methods for the same target molecule. The yields of compound **6** are 31 % and 71 %, using Methods 1 and 2, respectively (Figure 5a). Recrystallization by first heating up a reaction solvent and then cooling it down requires precise amounts of solvent at a given temperature to determine the end point, making it difficult to obtain optimal yields without visual feedback. An alternative to purification by recrystallization is the use of anti‐solvent to precipitate desired compounds out of a medium, washing solubilized impurities away in the filtrate. Method 1 involves a recrystallization, albeit at a low yield, whereas for Method 2, it was found that water is an effective anti‐solvent for the system at hand, allowing the isolation of purified target compound at a much higher yield. For both methods, the same reactionware design was used. The first 100 mL Reactor module with a 1‐inch threaded cap was used to react the mixture, the second 65 mL Filter Reactor module (Figure 5b), with the same type of cap, was used to isolate the product crystals (163 mg)/powder (350 mg) via recrystallization or filtration. In this procedure, to achieve efficient mixing and control over reaction mixture behavior, the diameter of the first module was increased to 50 mm. It was observed with lower volume reactionware modules the effervescence of the reaction mixture would result in premature material transfer over to the subsequent module or spillage. Increasing the volume of the polypropylene vessel allowed for controlled mixing and contained reaction mixture effervescence resulting from hydrochloric acid being added to a carbonate solution.

### Sulfa Drug Synthesis

**Figure 6:**
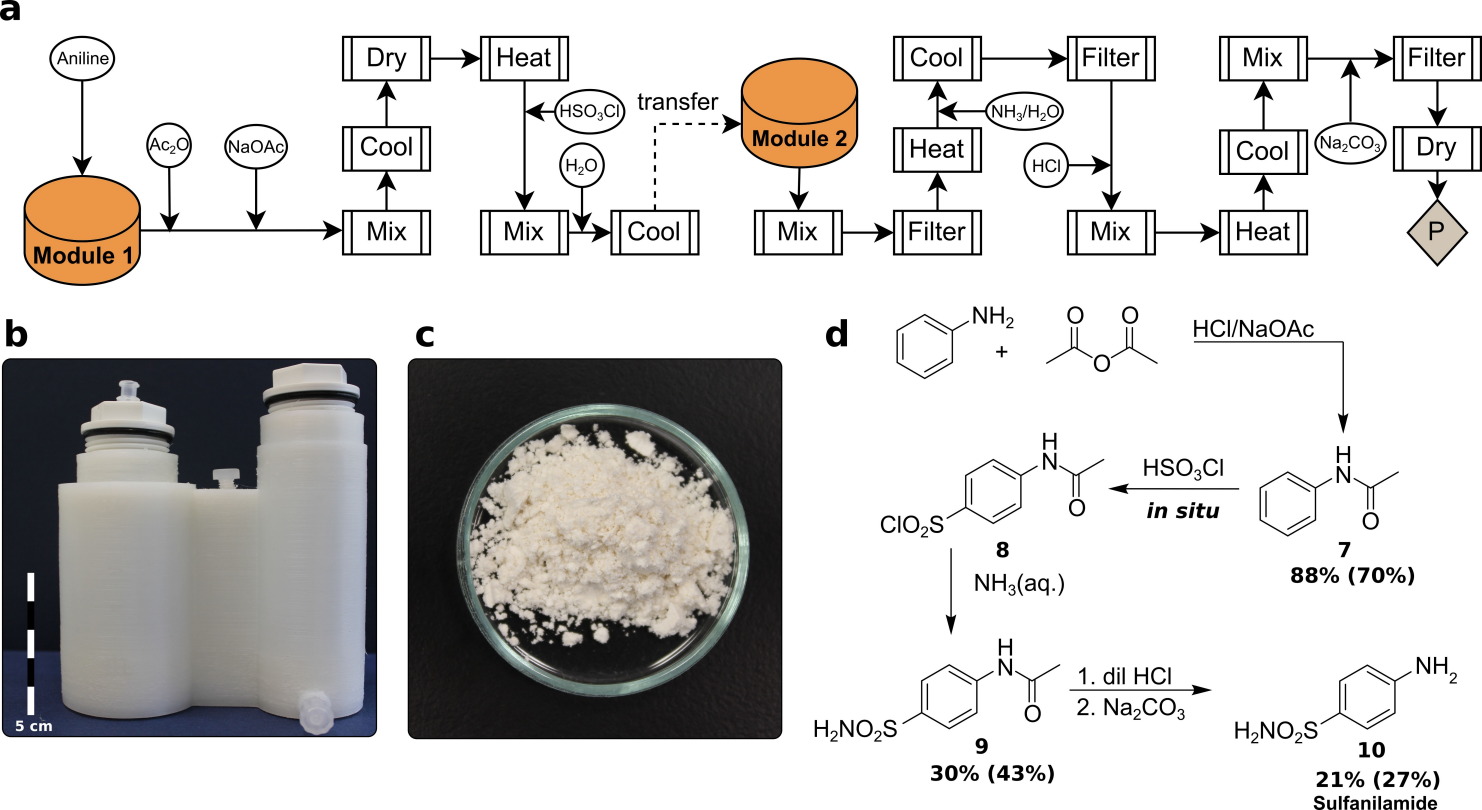
a) process flow diagram detailing unit operations for the synthesis of sulfanilamide (P: product), b) 3D‐printed reactionware as designed using *ChemSCAD*, c) Sulfanilamide product powder, d) reaction scheme for multi‐step sulfanilamide synthesis (%Yield of compound prepared in reactionware is in bold with %Yield as prepared in glassware is in parentheses).

In a final example we incorporated all aspects of unit operations (efficient mixing, cooling, drying, addition, filtration methods) and reactionware design formalized in the prior examples in a multi‐step^[25]^ a synthesis of a drug molecule—sulfanilamide (Figure 6). In particular, module enlargement for better stirring aided isolating larger yields of compound **7**. Albeit the process contains almost 40 unit operations, only two reactionware modules were required. Module 1—a 100 mL Filter Reactor with a threaded cap, and Module 2—a second Filter Reactor module of 70 mL with the same type of cap (Figure 6b). It starts with the dissolution of aniline in an aqueous acidic medium, where it is combined with acetic anhydride and a solution of sodium acetate under cooling. Powdered acetanilide (compound **7**) was isolated by filtration as a white crystalline powder. After extensive drying, chlorosulfonic acid was added, dissolving pre‐formed crystals, reacting them further with heating to produce *p‐*acetamidobenzenesulfonyl chloride (compound **8**) in situ. The mixture was added to ≈5 °C water, precipitating compound **8** which was isolated by filtration. The next step involved nucleophilic substitution of the chloride via the addition of conc. ammonia, and heating with mixing. Upon cooling, the *p‐*acetamidobenzenesulfonamide intermediate (compound **9**) crystallizes. In the final step, Sulfanilamide is formed by carrying out a deacetylation under acidic conditions, where *p‐*acetamidobenzenesulfonamide is dissolved in concentrated acidic aqueous media, then put under microwave irradiation for a half an hour using a conventional microwave oven at a low power setting. Finally, aqueous sodium carbonate is added to neutralize the medium as sulfanilamide, compound **10**, precipitates out as a white solid and is collected by filtration (379 mg) over four steps.

One challenge in the translation of the sulfanilamide protocol was achieving vacuum to sufficiently dry intermediates in reactionware cartridges. In the process, when compound **7** is formed, it is crucial to dry the crystalline powder well due to the reactive nature of chlorosulfonic acid as it will react vigorously even with trace amounts of water. It is essential for the print quality of the polypropylene vessel to be gapless. Moreover, to solve this, PTFE tape on fitting threads helped to achieve a vacuum of ≈25 mmHg. Also, the radius of Module 1 was increased to a slightly wider diameter of 46 mm to allow efficient mixing of the viscous reaction mixture to maximize the yield of compound **7**. A somewhat bigger challenge to translate the glassware procedure to reactionware was the final reaction step where a certain temperature was required for the compounds to react. The polypropylene will start to become malleable and deformed before the boiling point of aqueous conc. HCl and a solution‐state of the mixture is reached. To solve this, instead of heating in an oil bath, the mixture was put into a conventional microwave oven, allowing localized heating of the reaction mixture, but not the vessel itself. Then, base was added to reach a neutral pH, precipitating sulfanilamide, before isolating it via filtration.

Typically, when planning the execution of a targeted synthesis operation, reaction conditions are laid out to help predict the physical environment required for a molecule to react in a desired way. This determines which hardware, or objects, are required to generate the physical environment, and finally, unit operations determine the sequence and identity of actions that control a chemical process to achieve product. Currently chemists try to capture and report all the information required to reproduce or translate a chemical process to the best of their ability. However, a few left out details, that become tacit knowledge, can determine the difference between an unsuccessful or efficient chemical process. There is information loss with both aspects, reactivity and hardware, if approached traditionally. However, by using reactionware, chemists are forced to define and incorporate unit operations as a means of controlling a chemical process into decision making. Reactor design is an integral part of chemical process translation using reactionware, this is what requires unit operations to be explicitly defined for optimal process control and replication. Classifying unit operations by type and defining the precise order of sequence unit operations is an approach towards chemistry that is holistic,^[26]^ which is beneficial when developing more complex, intelligent process selection strategies using machine learning or other artificial intelligence techniques. Considering chemistry from an abstract perspective, allows the grouping of reactionware modules by chemical transformation. This way, modules can be combined easily later, when performing new and complex syntheses for chemical processes that have been observed before. It is worthwhile to mention that procedures might require adaptation when translating a process from glassware to reactionware if they were to perform better. This is seen from the Suzuki reaction presented in this paper, whilst Method 1 uses recrystallisation, Method 2 which involves precipitation provides a much higher yield.

**Figure 7:**
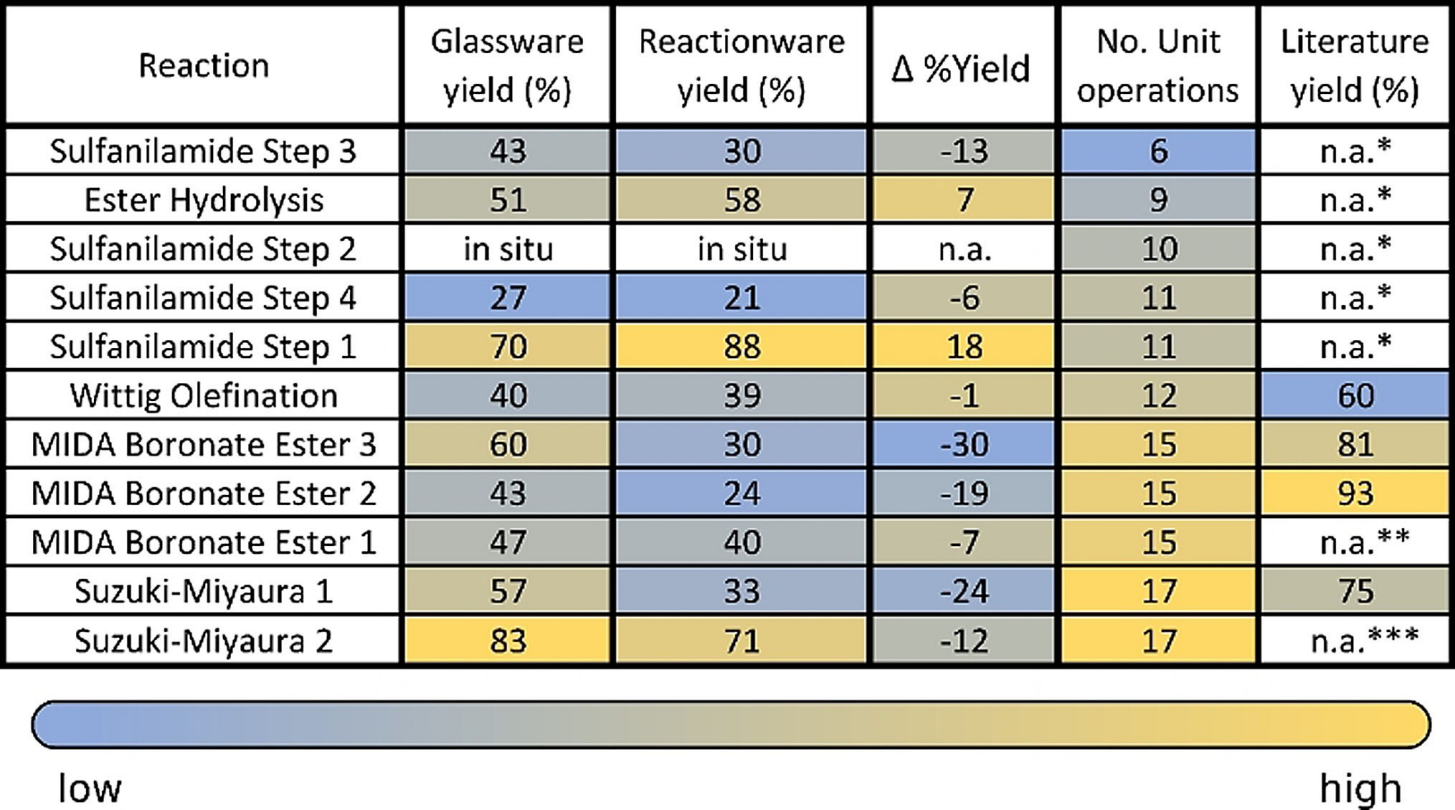
Comparison of yields obtained from literature procedures in glassware and translated procedures in reactionware. Entries sorted by the number of unit operations. The Δ %Yield is difference in %Yield between glassware and reactionware. * Yields not disclosed in student manuals. ** No literature data as this compound was prepared using a before‐unused substrate based on a general procedure. *** No literature data as this method was developed in‐house.

Chemical process translation from one platform to another requires a large amount of information to be captured explicitly to be successful. This can lead to loss of efficiency for a given process, as can be seen in the %Yield differences between original procedures (literature) and respective procedures as reproduced in glassware and the translated procedures in reactionware (Figure 7). Moreover, as the complexity of a process increases (No. unit op. >15, Figure 7), the Δ %Yield between original and translated processes increases accordingly. Yield loss can be explained by inherent challenges associated in moving synthesis to a different platform but also in part, by the accumulation of tacit information when performing multi‐step syntheses. Thus, chemical process translation between platforms can be optimized by removing tacit information in the reported procedures, or imbedding tacit knowledge in new approaches, much like reactionware replaces tacit knowledge that is imbedded in traditional approaches towards synthesis by making it intrinsically explicit in unit operation tables (see Supporting Information). Continued development of the reactionware approach should lead to substantially improved reproducibility in chemical synthesis.

## Conclusion

In conclusion, a cognitive approach to the digitization and control of organic synthesis was developed and expanded upon. This approach focuses on modularization and translation of chemical processes in a way that enables translation to reactionware‐based syntheses. Reactionware significantly reduces the amount of tacit knowledge that is imbedded in traditional approaches towards synthesis. This reactionware approach also stands to help standardize chemistry processes and increase their reproducibility. Reactionware is also partly digital, allowing the development of a database of reactionware‐based protocols.

The reactionware approach also has substantial potential to facilitate teaching of organic synthesis. This paper specifically describes 11 chemical processes that are ready to be applied in undergraduate teaching as 3D printed reactionware‐based experiments. The approach is also amenable to online sharing of 3D printed reactor‐based organic chemistry experiments. Finally, this non‐specialist‐friendly approach has substantial potential to help expand access to the molecule making process.

## Data Availability

Item 1: Design files of reactionware and high‐surface reflux con‐denser (.stl, .gcode, .ccad).

Item 2: Supporting Information comprised of two Sections: “Section A—Glassware and reactionware procedures for target com‐pounds” and “Section B—Laboratory tutorials for students”.

Item 3: Exercise file for guiding students through the sulfanilamide synthesis project.

Item 4: File describing installation of ChemSCAD.

## Conflict of interest

L.C. is the inventor on a patent entitled “Digital Reactionware” Publication number: 2020033518. The University of Illinois has filed patent applications related to MIDA boronates.

1

## Supporting information

As a service to our authors and readers, this journal provides supporting information supplied by the authors. Such materials are peer reviewed and may be re‐organized for online delivery, but are not copy‐edited or typeset. Technical support issues arising from supporting information (other than missing files) should be addressed to the authors.

Supporting Information

Supporting Information

Supporting Information

Supporting Information

## Data Availability

The data that support the findings of this study are available in the supplementary material of this article.
