# Supplementary material for: Digitizing Chemical Synthesis in 3D Printed Reactionware
Source: Angew Chem Int Ed Engl. 2022 Mar 25;61(24):e202116108. doi: 10.1002/anie.202116108 (PMC9186708; doi:10.1002/anie.202116108)
Supplement: Supplementary file 2 — Supporting Information [file ANIE-61-0-s004.zip › Exercise.docx]

**Process selection and reactor design for multi-step sulfanilamide synthesis**

**Workflow:**

- Two methods of synthesizing an antibacterial drug - sulfanilamide - are provided. Choose one which is more overall viable based on such factors as safety, environmental friendliness, efficiency. Write a short paragraph contrasting the routes. When looking to choose between two processes or optimising an already established chemical process there are some key things which one must consider. These include but are not limited to: number of steps, number of unit operations (heating, mixing, filtering, crystallizing), difficulty involved carrying out individual unit operations, material cost and hazards, use of high-energy profile equipment, total synthesis time, waste produced, etc.
- Identify the unit operations within the process.
- Prepare a process flow diagram (PFD). An example is provided in the resources section (Fig. 3).
- Install ChemSCAD, use the software to generate a 3D model of a reactor system capable of performing the process outlined in the PFD on a bench scale (20-100 mL reactors).
- Save reactionware models as .ccad files using ChemSCAD and turn in those files as answers.

**Route 1**

**Ref.: Williamson, K.L. Macroscale and Microscale Organic Experiments; Heath: Lexington, MA, 1994; pp 541-557. ^1^**


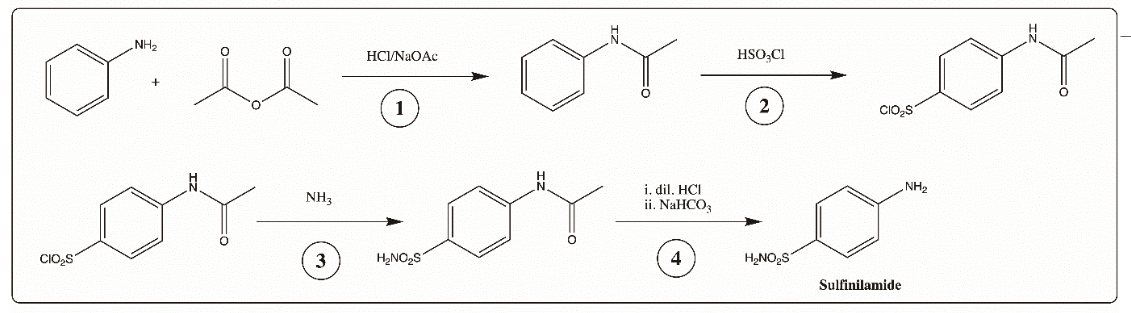


**Figure 1:** sulfonilamide synthesis scheme – route 1.

Step 1 Acetanilide: Dissolve 2 g of aniline in 60 mL water and 2 mL conc. HCl in a 125 mL Erlenmeyer flask. If the solution is colored, vacuum filter it through a pad of decolorizing Carbon (charcoal). Measure out 2.4 mL of acetic anhydride and prepare a solution of 2 g of sodium acetate in 12 mL of water. Add the acetic anhydride to the solution of aniline with stirring, and at once add the sodium acetate solution. Stir the mixture for ~ 5 mins, cool it in ice, and collect the product acetanilide. This material needs to be completely dry before the next step. Characterize the acetanilide (fluffy white crystals). [Please note:- If crystals are not soluble in dichloromethane, then take the IR in mineral oil]

Step 2 p-Acetamidobenzenesulfonyl chloride: Place 2 g of dry acetanilide in a dry 25-mL Erlenmeyer flask. Add 5 mL of chlorosulfonic acid (corrosive and reacts violently with water) a few drops at a time using a Pasteur pipet (no metal needles!). After ~10 min the reaction should subside and almost all of the acetanilide will have dissolved. Heat the mixture in a hot water bath for ~10 min to complete the reaction. Pipet the mixture slowly with stirring into 24 mL of ice water in another 125-mL Erlenmeyer flask (use extreme caution). Rinse the reaction flask with cold water and stir the product until an even suspension of white solid is obtained. Vacuum filter the pacetamidobenzenesulfonyl chloride (Chalky-white solid) and wash it with water. [Please note:- If crystals are not soluble in dichloromethane, then take the IR in mineral oil] p-

Step 3 Acetamidobenzenesulfonamide: Transfer the moist solid to the rinsed 125-mL Erlenmeyer and add 9 mL of concentrated ammonia (ammonium hydroxide) and 9 mL of water. Heat the mixture to just below the boiling point on a hot plate with occasional swirling for 5 min. Cool the mixture in an ice bath and collect the pacetamidobenzenesulfonamide (very small white needle like crystals) by suction filtration and allow it to drain thoroughly. [Please note:- If crystals are not soluble in dichloromethane, then take the IR in mineral oil]

Step 4 Sulfanilamide: Transfer the moist solid to a 125-mL erlenmeyer, add 3 mL of con HCl and 6 mL of water. Boil the mixture gently until the solid dissolves and then continue heating at the boiling point for ~10 min longer (do not evaporate to dryness). Cool the solution to room temperature. No solid should deposit (v. light beige colored clear solution). If solid is seen, continue heating for a while longer. To the cool solution (on ice), add a saturated aqueous solution of 2 g of sodium bicarbonate until the solution is neutral to pH paper (still keeping the flask on ice). Cool the resulting solid on ice and vacuum filter the sulfanilamide.

Recrystallize the sulfanilamide from minimum amount of water. Characterize sulfanilamide (white product) with H1 -NMR (in DMSO solvent), IR, and melting point. Assign all characteristic data points of the material and comment on changes in the spectra through each step from starting material through product. [Please note:- If crystals are not soluble in dichloromethane, then take the IR in mineral oil]

**Route 2**

**Ref.:** [**https://worldwide.espacenet.com/publicationDetails/biblio?CC=CN&NR=105175294B&KC=&FT=E&locale=en_EP**](https://worldwide.espacenet.com/publicationDetails/biblio?CC=CN&NR=105175294B&KC=&FT=E&locale=en_EP)


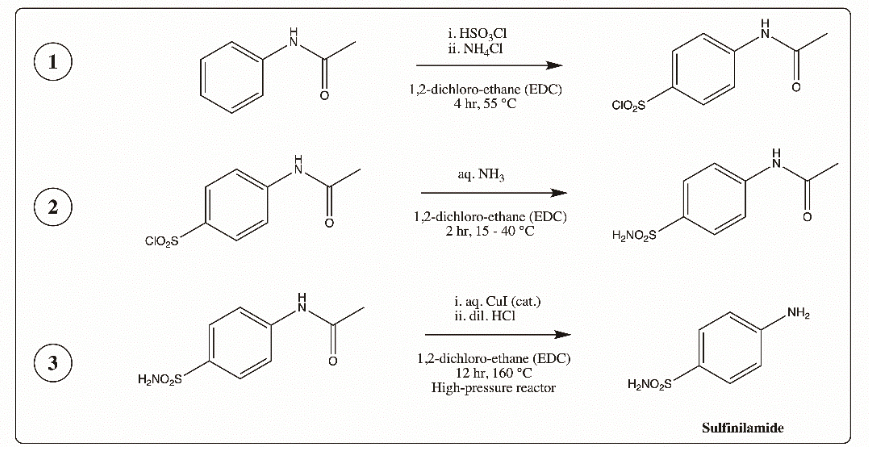


**Figure 2:** sulfonilamide synthesis scheme – route 2.

The present invention relates to a method for synthesizing sulfanilamide by using chlorobenzene as a raw material. The method for synthesizing sulfanilamide by using chlorobenzene as the raw material is characterized by comprising the following steps: (a) adding ethylene dichloride and ammonium chloride, and dropwise adding chlorosulfonic acid into a reaction vessel, heating the reaction vessel to 50~60 DEG C and dropwise adding chlorobenzene to react continuously for 2-5h, and cooling the reaction vessel to 15~20 DEG C to obtain a first mixed liquid, wherein the mass ratio of the ethylene dichloride, the ammonium chloride, the chlorosulfonic acid and the chlorobenzene is (20~30):1:(20~30):(10~15); and (b) dropwise adding the first mixed liquid into ammonia water with concentration of 22~25%, after stirring and reacting the mixture for 0.5~1 hour, heating the mixture to 40~42 DEG C, continuously stirring and reacting the mixture for 1~2 h; then transferring the mixture to a high pressure reaction vessel, adding a catalyst, heating the mixture to 160~200 DEG C, carrying out reaction for 10~12 h; performing cooling, pressure relieving, steaming out the excess ammonia, adjusting the pH to 6.5~6.7, precipitating the solid, and after filtration and separation, performing drying, wherein the mass ratio of the ammonia water, the catalyst and the chlorobenzene is (120~150):1:(8~15). By using chlorosulfonic acid as sulfonation and sulfonyl chlorination reagents, chlorobenzene sulfonyl chloride can be synthesized in one step, and sulfanilamide can be obtained by ammonolysis. The materials directly enter into a high-pressure vessel for ammonolysis without an intermediate separation process, so that the method is relatively short in route, easy to operate and high in total product yield.

**Resources**

**
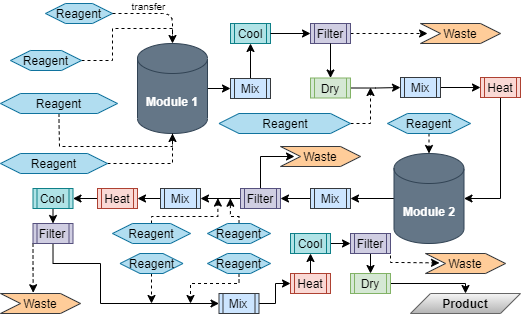
**

**Figure 3:** Example process flow diagram (PFD).

- PFD’s can easily be prepared using https://app.diagrams.net/
