# Supplementary material for: Digitizing Chemical Synthesis in 3D Printed Reactionware
Source: Angew Chem Int Ed Engl. 2022 Mar 25;61(24):e202116108. doi: 10.1002/anie.202116108 (PMC9186708; doi:10.1002/anie.202116108)
Supplement: Supplementary file 2 — Supporting Information [file ANIE-61-0-s004.zip › Installing ChemSCAD.docx]

**Installing ChemSCAD – March 2021**

ChemSCAD requires Python **3.6** or above. Tested and working with latest Python release (3.8).

See installation video instructions here: Z:\group\0-Papers in Progress\0-in-press\ChemSCAD-WDH\After acceptance\ChemSCAD_installation_updated.mp4

ChemSCAD was tested with OpenSCAD 2019-05 (most up-to-date version as of March 2020)

- Install OpenSCAD for your operating system as follows:

For Windows:

All OpenSCAD dependencies including the binaries are included in the setup.py for ChemSCAD, so will be installed automatically when installing the requirements.txt file.

Therefore, please proceed to the ChemSCAD installations instructions below.

For Mac:

OpenSCAD can be installed using the .dmg installable from the following link: <https://files.openscad.org/OpenSCAD-2019.05.dmg>

Install this .dmg file, making sure to accept all permissions in Security & Privacy from within System Preferences.

**ChemSCAD installation for Windows:**

Prior to installing ChemSCAD it is recommended to make a virtual environment (venv) in order to not have any conflicts with existing installed pip packages.

py -m pip install --user virtualenv

py -m venv chemscad-env

.\chemscad-env\Scripts\activate

PyQt5 *may* have to be installed manually, dependent on your installation. Install with:

pip install PyQt5

Installation is simple, simply pip-install the chemscad package:

pip install chemscad

ChemSCAD can now be run from a command line using the command: chemscad
