# Supplementary material for: Digitizing Chemical Synthesis in 3D Printed Reactionware
Source: Angew Chem Int Ed Engl. 2022 Mar 25;61(24):e202116108. doi: 10.1002/anie.202116108 (PMC9186708; doi:10.1002/anie.202116108)
Supplement: Supplementary file 4 — Supporting Information [file ANIE-61-0-s003.pdf]

Angewandte Chemie
Eine Zeitschrift der Gesellschaft Deutscher Chemiker

Supporting Information

Digitizing Chemical Synthesis in 3D Printed Reactionware

A. Bubliauskas, D. J. Blair, H. Powell-Davies, P. J. Kitson, M. D. Burke, L. Cronin**

Contents

Section A: Glassware and reactionware procedures for target compounds 4

Unit operations and operation tables 4

Applying unit operations in practice: material transfer between reactionware modules 4

1. Supplementary methods 5

1.1. Reactionware print postprocessing and assembly of reactionware cartidges 5

1.2. Reactionware module specifications 7

1.3. 3D printing settings 8

1.4. Common reactionware questions 9

2. MIDA boronate esters 10

2.1. 3-methylphenylboronic acid 10

2.1.1. Glassware synthesis 10

2.1.2. Reactionware synthesis 12

2.1.3. Operations Table 14

2.2. 3-bromophenylboronic acid 15

2.2.1. Glassware synthesis 15

2.2.2. Reactionware synthesis 16

2.2.3. Operations Table 18

2.3. Pentafluorophenylboronic acid MIDA ester 19

2.3.1. Glassware synthesis 19

2.3.2. Reactionware synthesis 21

2.3.3. Operations Table 23

3. Ester hydrolysis 24

3.1. Glassware synthesis 24

3.2. Reactionware synthesis 24

3.2.1. Operations Table 26

4. Wittig olefination 27

4.1. Glassware synthesis 27

4.2. Reactionware synthesis 28

4.2.1. Operations Table 30

5. Suzuki coupling 31

5.1. Method 1 31

5.1.1. Glassware synthesis 31

5.1.2. Reactionware synthesis 32

5.1.1. Operations Table 34

5.2. Method 2 35

5.2.1. Glassware synthesis ... 35
5.2.2. Reactionware synthesis ... 37
5.2.1. Operations Table ... 39
6. Sulfanilamide synthesis ... 41
6.1. Glassware synthesis ... 41
6.2. Reactionware synthesis ... 41
6.2.1. Operations Table ... 45
Section B: Laboratory Tutorials for Students ... 48
7. MIDA boronate formation: preparing coupling reagents ... 49
Introduction ... 49
Experimental procedure: ... 49
Pre-lab and post-lab questions: ... 50
8. Ester hydrolysis: FGIs and identification of unknown compound ... 52
Introduction ... 52
Pre-lab questions ... 52
Experimental procedure ... 54
Post-lab questions ... 55
9. Wittig olefination: forming C=C bonds ... 56
Introduction ... 56
Experimental procedure: ... 56
Pre-lab questions & post-lab questions: ... 57
10. Suzuki-Miyaura: forming C-C bonds ... 59
Introduction ... 59
Method 1/2 ... 59
Experimental procedure ... 59
Method 2/2 ... 61
Experimental procedure ... 61
Pre- and post-lab questions: ... 62
11. Synthesis of sulfanilamide: preparing drug molecules ... 64
Introduction ... 64
Pre-lab questions ... 65
Experimental procedure ... 70
References ... 73
Appendix ... 74

Section A: Glassware and reactionware procedures for target compounds

Unit operations and operation tables

Operation tables are provided with each reactionware synthesis. These should aid users in understanding the procedural flow while carrying out that particular synthesis in reactionware. Icon meanings are summarized in Figure 1 below.

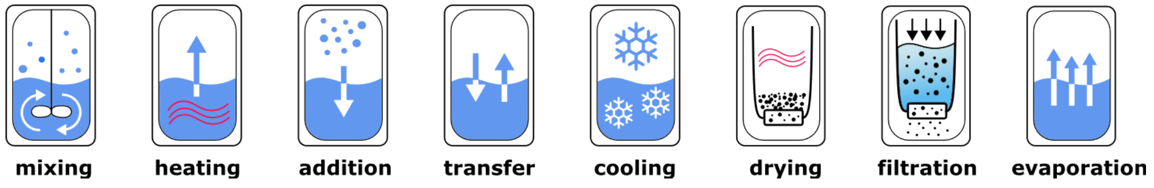

Figure 1: Unit operation icons and their meanings

Applying unit operations in practice: material transfer between reactionware modules

In reactionware, it is possible to transfer a reaction mixture from one reactor to another via applying a controlled flow of compressed air / nitrogen gas (by way of a thread screw to control the flow rate). For example, a nitrogen line would attached to the top Luer inlet of Module 1 and the cannula screw between Module 1 and Module 2 will be released slightly to allow the steady transfer of the mixture from Module 1 to Module 2, under constant stirring. The flow rate can be easily adjusted by way of further loosening the screw to increase the flow rate, allowing for fine control over this depending on mixture viscosity.

1. Supplementary methods

Solvents and reagents were used as received from commercial suppliers unless otherwise stated. Polypropylene feedstock for 3D printing was purchased from Barnes Plastic Welding Equipment Ltd., Blackburn, UK. 3D printing was achieved on Ultimaker 2+ FDM 3D printers supplied by Ultimaker and modified by the authors to print with polypropylene. ^{1}H, ^{13}C NMR spectra were recorded on a Bruker Avance III HD 600 MHz and Bruker Avance II 400 MHz spectrometers. Chemical shifts are reported in ppm relative to residual solvent (multiplicities are given as s: singlet, d: doublet, t: triplet, q: quartet, m: multiplet, with coupling constants reported in Hz). Infrared Spectroscopy: All samples were collected in transmission mode using an ATR fitted JASCO FT-IR-410 spectrometer. Wavenumbers are given in cm^{-1}.

1.1. Reactionware print postprocessing and assembly of reactionware cartidges

After 3D printing, reactionware monoliths (module systems) are post-processed manually by drilling cavities and making threads manually to accommodate fittings such as Luer adaptors, screw-valves, etc. Reactionware monolith post-processing is described below and a bill of materials is presented for any parts that are needed for the post-processing.

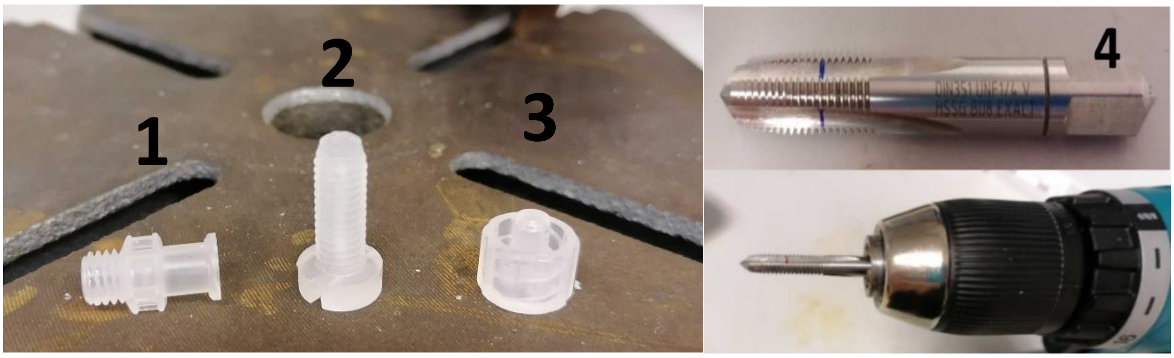

Figure 2: Common fittings that are used in reactionware. **1** - polypropylene Luer adaptor fo fit syringes/caps/high surface condensers; **2** - screw-valve; **3** – Luer adaptor cap, **4** – thread tap.

Reference number	Part	Part no	Company	Link
**1**	Masterflex Fitting, Polypropylene, Straight, Female Luer to Threaded Adapter, 1/4-28 UNF	WZ-45508-66	Cole-Parmer	https://www.coleparmer.com/i/cole-parmer-female-luer-x-1-4-28-unf-thread-pp-25-pk/4550866?searchterm=WZ-45508-66
**2**	M6 X 16mm Cheese Head Screws - Polypropylene	SFE-M6-16-PP	Accu	https://www.accu.co.uk/en/cheese-head-screws/496635-SFE-M6-16-PP

**3**	Cole-Parmer Animal Free Male Luer Lock Plug, PP, 25/pk	SI-30800-30	Cole-parmer	https://www.coleparmer.com/i/cole-parmer-female-luer-x-1-4-28-unf-thread-pp-25-pk/4550866?searchterm=WZ-45508-66
**4**	RS PRO HSS 1/4-28 Straight Flute Tap Set, 56 mm Length	668-1093	RS Components	https://uk.rs-online.com/web/p/threading-taps/6681093/

Table 1: Links for purchasing parts 1-4.

Cavities to accommodate adaptors are made using a hand-held drill using various drill bits.

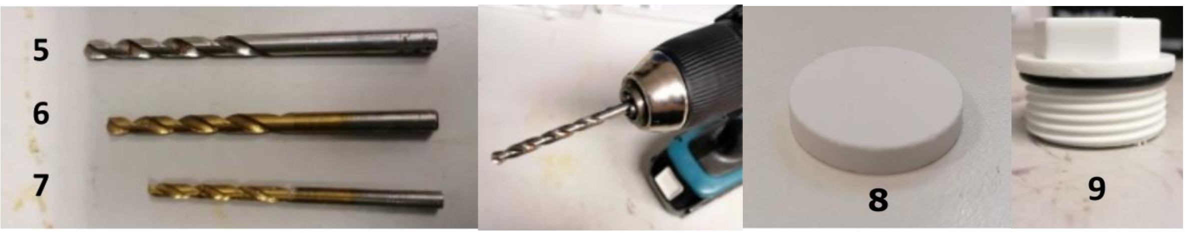

Figure 3: From left to right: **5**-**7** drill bits of various sizes are used for making cavities to accomodate different adaptors. **8** – glass filter disc. **9** – 1” screw-cap.

Reference number	Part
**5**	Drill bit 5.4 mm diameter
**6**	Drill bit 5.0 mm diameter
**7**	Drill bit 4.0 mm diameter

Table 2: Sizes of drill bits used for reactionware print post-processing.

**8**	Duran filter discs (porosity 3)	SCOT251520304	VWR	https://uk.vwr.com/store/catalog/product.jsp?catalog_number=SCOT251520201#stiboGrid
**9**	1” Polypropylene cap			https://www.aliexpress.com/item/32960615886.html?spm=a2g0o.productlist.0.0.28c63478Dk6cBO&algo_pvid=a2c62159-a647-4a3c-84fd-0de884775b7a&algo_exp_id=a2c62159-a647-4a3c-84fd-0de884775b7a-9&pdp_ext_f=%7B%22sku_id%22%3A%226650389692 7%22%7D

Table 3: Links of suppliers for purchasing filter discs and screw caps.

1.2. Reactionware module specifications

Reactionware$_{Module}$	A		B	C		D		E	
Reactor$_{Type}$	Filter Reactor	Filter Reactor	Filter Reactor	Reactor	Filter Reactor	Reactor	Filter Reactor	Filter Reactor	Filter Reactor
Reactor$_{Volume}$	100	100	60	50	50	100	65	100	73
Top$_{Type}$	CRWCO	CRWCO	CRWO	CRWO	CRWCO	CRWCO	CRWCO	CRWCO	CRWCO
Bottom$_{Type}$	RWIO	RWEO	RWEO	RWIO	RWEO	RWIO	RWEO	RWIO	RWEO
No$_{Inputs/Outputs}$	2 / 1	2 / 1	1 / 1	2 / 1	2 / 1	1 / 1	2 / 1	1 / 1	2 / 1
Diameter$_{Internal\ Pipe}$ (mm)	3	3	3	3	3	4	4	3	3

Table 4: Reactionware module specifications necessary for recreating same reactionware models using *ChemSCAD* software, where: A – reactionware for MIDA boronate ester formation, B – Wittig Olefination, C – Ester Hydrolysis, D – Suzuki-Miyaura coupling, E – Sulfanilamide synthesis. Abbreviations meaning for reproducing models in *ChemSCAD*: CRWO - Closed Round With Outlet, CRWCO - Closed Round with Custom Outlet, RWIO - Round with Internal Outlet, RWEO – Round with External Outlet.

1.3. 3D printing settings

Reactionware was 3D printed using polypropylene filament (3 mm) using the following settings in Cura:

Quality	Value
Layer Height	0.3 mm
Initial Layer Height	0.4 mm
Line Width	0.6 mm

Shell	Value
Wall Thickness	1.2 mm
Wall Line Count	2 mm
Outer Wall Wipe Distance	0.3 mm
Top/Bottom Thickness	3 mm
Top Layers	10
Bottom Layers	10
Top/Bottom Pattern	Lines
Bottom Pattern Initial Layer	Lines
Compensate Wall Overlaps	Yes

Infill	Value
Infill Density	100 %
Infill Line Distance	0.6 mm
Infill Pattern	Lines
Infill Line Multiplier	1
Infill Layer Thickness	0.3 mm

Material	Value
Initial Layer Flow	140 %
Enable Retraction	Yes
Retraction Minimum Travel	1.2 mm
Maximum Retraction Count	12
Minimum Extrusion Distance Window	1 mm
Nozzle Switch Retraction Distance/Speed	20 mm/s

Speed	Value
Print Speed	15 mm/s
Outer/Inner Wall Speed	10 mm/s
Top/Bottom Speed	15 mm/s
Support Speed	20 mm/s
Travel Speed	40 mm/s
Initial Layer Speed	10 mm/s
Enable Acceleration Control	Yes
Print Acceleration	4000 mm/s^2
Wall Acceleration	1000 mm/s^2

Travel	Value
Avoid Printed Parts When Travelling	Yes
Z hop When Retracted	Yes

Cooling	Value
Enable Print Cooling	Yes
Fan Speed	50 %
Minimum Layer Time	15 s
Minimum Speed	10 mm/s

Support	Value
Generate support	Yes
Support Overhang Angle	60°
Support Density	15%

Build Plate Adhesion	Value
Build Plate Adhesion Type	Raft
Raft Top Layers	2
Raft Top Layer Thickness	0.3 mm
Raft Print Speed	10 mm/s

1.4. Common reactionware questions

Why are Reactionware printed using polypropylene?

The modules are 3D printed using polypropylene and the limitations are in-line with the boundaries of the physical properties of polypropylene. Polypropylene starts becoming malleable at around 140 °C, thus the maximum recommended temperature for reactionware modules should not exceed 120 °C or reactors will start deforming. Reactionware cartridges have been shown to withstand pressures of around 5 bar. Reactionware modules are compatible with most strong bases and acids, some strong acids (like chlorosulfonic acid used in the sulfanilamide synthesis as presented in the paper) will discolorise reactionware. As for solvent compatibility, reactionware can be used with most solvents. However, swelling of cartridges was observed when reactions with toluene/xylene have been carried out at higher temperatures (>80 °C), same was observed with dimethylformamide. Other materials for reactionware cartridges are available. Cartridges with synthetically-useful applications could be manufactured using nylon, as well as PEEK (polyether ether ketone).

Are there challenges associated with performing multiple steps in reactionware?

Generally, the more reactionware modules, the lower the final yield. This is due to reaction material that might be leftover in previous reactors. The purity could be affected by increasing the number of modules too, this depends on the chemical process at hand. Say a substitution reaction is followed by an oxidation and then purification. If this process is carried out in one module, all material is contained in one module. For example in a three module system of (Modulesubstitution -> Moduleoxidation -> Modulepurification) if transfers are incomplete, the Modulesubstitution will have leftover reaction mixture, which affects stoichiometry in Moduleoxidation, where ‘extra’ oxidation agent might over-oxidise the substrate, resulting in impurities.

What are the topological considerations of reactionware design?

Module templates are based on designs which have shown good mass transfer properties for the volumes used. The modules were primarily designed based on reaction scale (i.e. anticipated reaction volume) and proposed synthetic techniques which determine the number and type of inlets/outlets. During reactionware development mass transfer rates are also considered. For example, if during experiment iteration, mixing is insufficient, the radius of the module may be increased to allow better mixing by accommodating larger stir bars.

2. MIDA boronate esters

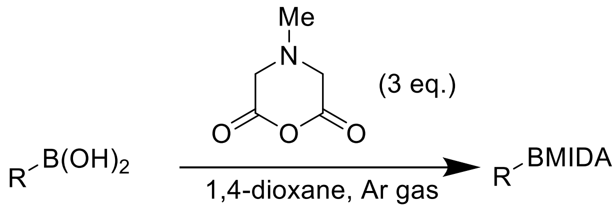

Scheme 1: general scheme for the preparation of MIDA-functionalised boronic acids.

The following procedures have been adapted from the general procedure provided by Burke et al. in their recent work.[1] The work-up procedure was altered to allow an anti-solvent approach, leading to precipitation of the desired MIDA ester product.

2.1. 3-methylphenylboronic acid

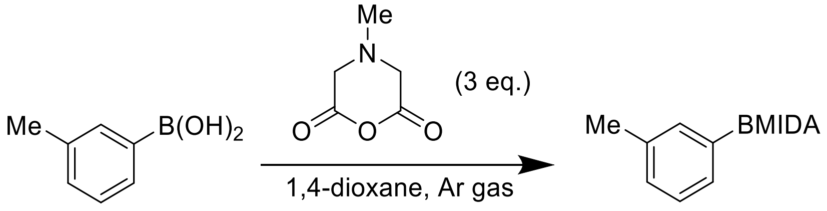

1

Scheme 2: synthetic scheme for the preparation of 3-methylphenyl MIDA boronic ester (compound **1**).

2.1.1. Glassware synthesis

3-Methylphenylboronic acid (136 mg, 1 mmol, 1 eq.) and MIDA anhydride (3 mmol, 3 eq.) was dissolved in dry dioxane (2.5 mL). The glass vial was capped and was flushed with argon gas using a balloon. The vial containing the mixture was suspended in a pre-heated 70 °C oil bath and stirred for 24 h. After stirring for named time, the mixture was allowed to cool to RT. Deionized water (18 µL, 1 mmol, 1 eq.) and dioxane (2.5 mL) was added to the solution and stirred for an hour. The mixture was transferred to a small round bottom flask and evaporated using a rotary evaporator. 5 mL of acetone was added to the flask under stirring, forming a suspension. Then the suspension was filtered and the filtrate collected. To the filtrate was added a Et_2O:hexane (1:1, 40 mL) mixture and stirred. A precipitate formed which was then filtered, dried in a desiccator under vacuum and **1** was collected as a white solid (116 mg, 47 % yield).

^{1}H NMR (600.1 MHz; 303 K; DMSO; δ, ppm): 7.26-7.16 (4H, m), 4.34-4.29 (2H, d), 4.12-4.08 (2H, d), 2.49 (3H, s), 2.31 (3H, s).

^{13}C NMR (150 MHz; 303 K; DMSO; δ, ppm): 169.9, 136.9, 133.4, 130.0, 129.9, 128.1, 62.3, 48.2, 21.7.

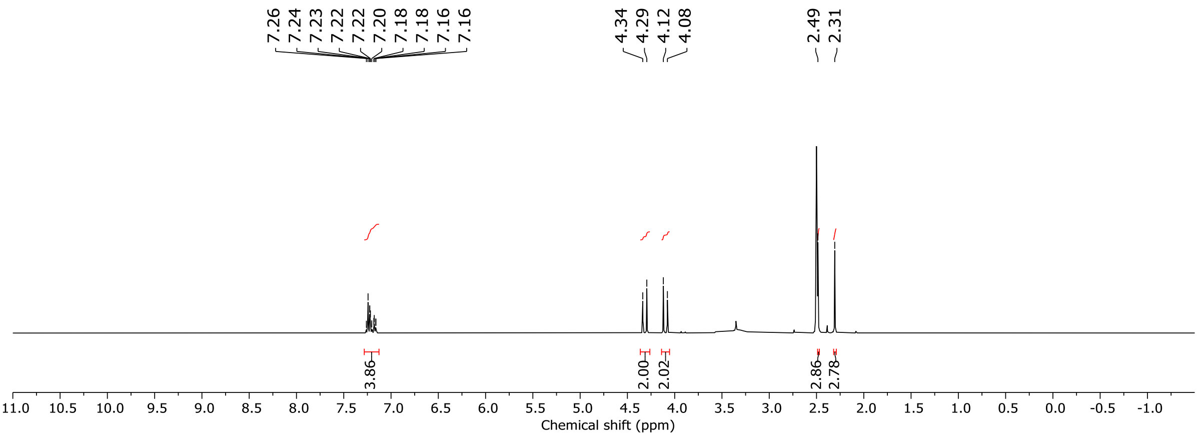

Figure 4: ^{1}H NMR (d_6-DMSO) of 3-methylphenylboronic MIDA ester synthesised in glassware (compound **1**).

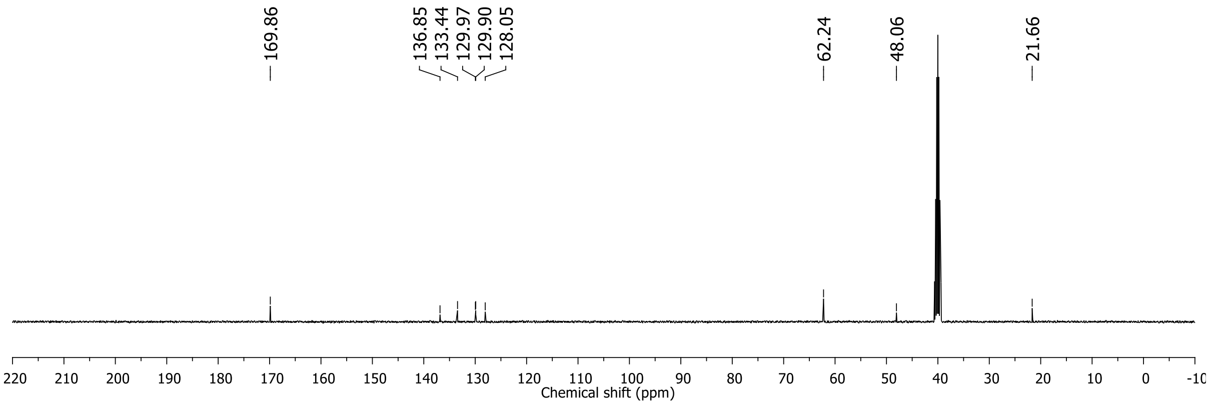

Figure 5: ^{13}C NMR (d_6-DMSO) of 3-methylphenylboronic MIDA ester synthesised in glassware (compound **1**).

2.1.2. Reactionware synthesis

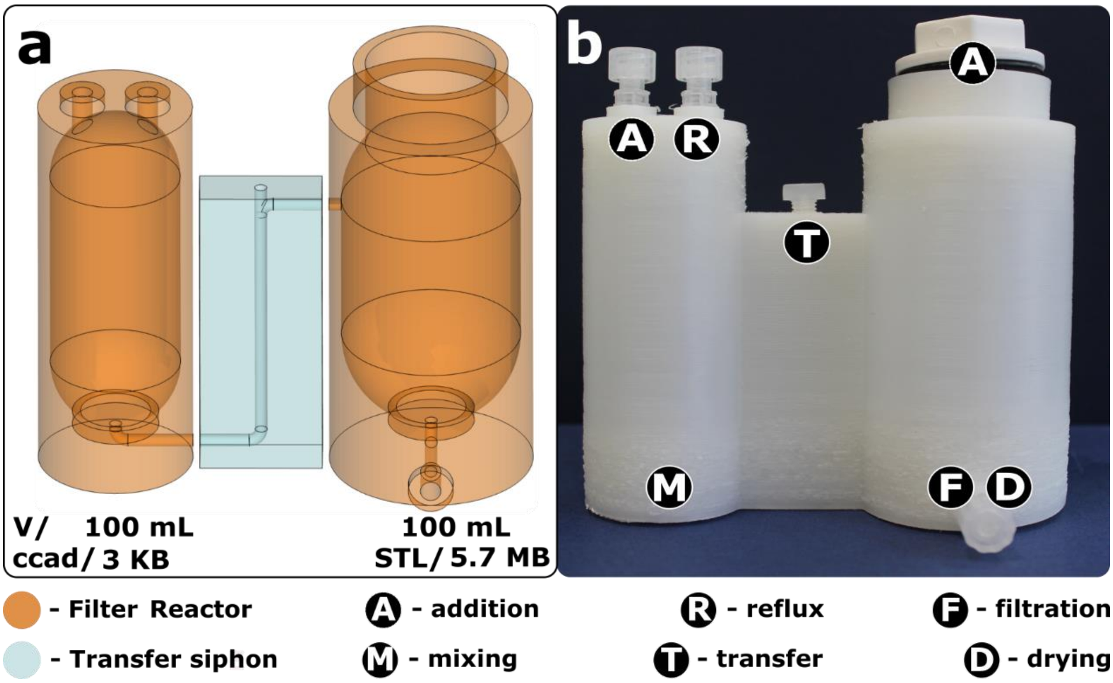

Figure 6: Reactionware for MIDA boronate ester formation, where **a** –transparent model with individual volumes of each Filter Reactor and associated file sizes, **b** – front view of real model, associated unit operations, their types and locations are highlighted (see legend).

3-Methylphenyl boronic acid (136 mg, 1 mmol, 1 eq.) and MIDA anhydride (387 mg, 3 mmol, 3 eq.) was charged to a flame-dried beaker along with anhydrous 1,4-dioxane (2.5 mL) and dissolved under stirring. Module 1 was flushed with a ballon of argon gas. The prepared mixture was transferred to Module 1 using a syringe. A 3D-printed high surface condenser was attached to Port 1, and all other ports were closed shut before lowering reactionware into a pre-heated oil bath (100 °C), where it was left to stir for 24 h. After stirring, the mixture was allowed to cool to RT. H_2O (18 µL, 1 mmol, 1 eq.) and 1,4-dioxane (2.5 mL) was added to Module 1 and left to stir for 1 hour. Reactionware was lowered into a pre-heated oil-bath (50 °C), and a vacuum line was attached to Module 1 and the solvent evaporated (24 hours, 50 mmHg). Once dry, acetone (5 mL) was charged to Module 1 and the reaction was stirred for 20 minutes. A mixture of Et_2O:hexane (40 mL, 1:1) was added to Module 2. Reaction mixture was transferred from Module 1 to Module 2 whilst stirring in Module 2. Module 2 was left to stir for 30 minutes, whilst a white solid precipitated. A vacuum line was attached to the bottom port of Module 2 and a filtration was carried out. The product, **1**, was dried in reactionware overnight and isolated as a white powder 104 mg, 40% yield).

^{1}H NMR (600.1 MHz; 303 K; DMSO; δ, ppm): 7.26-7.16 (4H, m), 4.34-4.29 (2H, d), 4.12-4.08 (2H, d), 2.48 (3H, s), 2.31 (3H, s).

^{13}C NMR (150 MHz; 303 K; DMSO; δ, ppm): 169.9, 136.9, 133.4, 130.0, 128.1, 62.2, 48.1, 21.7.

ν_{max} (neat, cm^{-1}) 3011, 2966, 1773, 1747,1464, 1420, 1402, 1335.

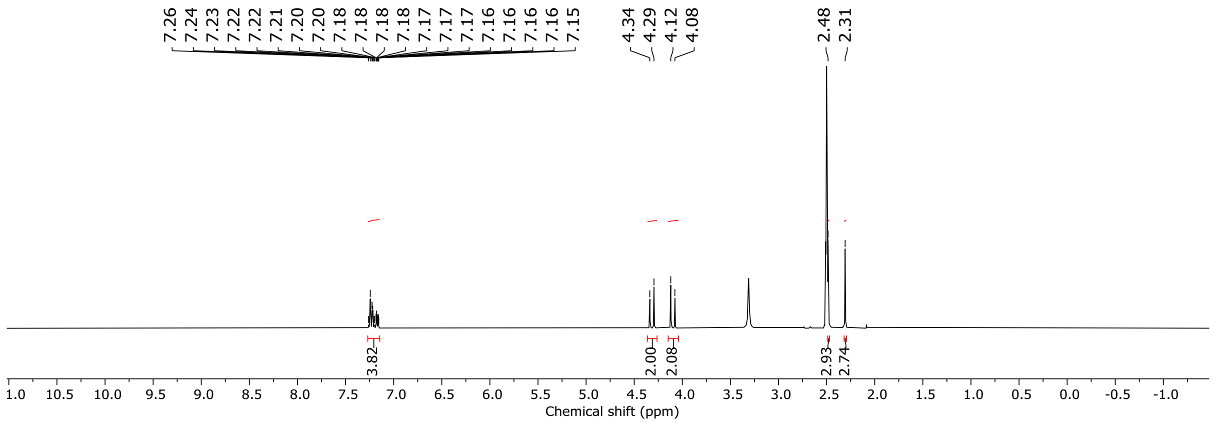

Figure 7: ^{1}H NMR (d_6-DMSO) of 3-methylphenylboronic MIDA ester synthesised in reactionware (compound **1**).

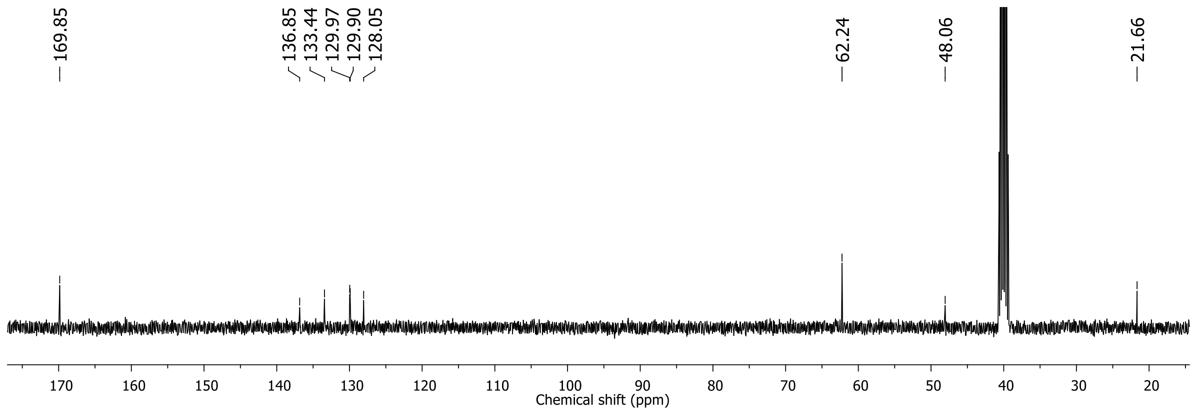

Figure 8: ^{13}C NMR (d_6-DMSO) of 3-methylphenylboronic MIDA ester synthesised in reactionware (compound **1**).

2.1.3. Operations Table

Operation	Icon	Reagent (g, mL)	From	To	Solvent (mL)	Temp (°C)	Stir Rate (RPM)	Time (min)
**Add**		3-methylphenyl boronic acid (136 mg, 1 mmol, 1 eq.)	Spatula	Module 1		RT	0	0.5
**Add**		MIDA anhydride (387 mg, 3 mmol, 3 eq.)	Spatula	Module 1		RT	0	0.5
**Add**			Syringe	Module 1	1,4-dioxane (2.5 mL)	RT	0	0.5
**Mix**				Module 1		RT	250	5
**Heat**				Module 1		100	250	1440
**Cool**				Module 1		RT	250	30
**Add**		H_2O (18 µL, 1 mmol, 1 eq.)	Syringe	Module 1	1,4-dioxane (2.5 mL)	RT	250	5
**Mix**				Module 1		RT	250	60
**Heat**				Module 1		50	250	30
**Dry**				Module 1		50	250	1440
**Add**			Syringe	Module 1	Acetone (5 mL)	RT	250	20
**Add**			Syringe	Module 2	Et_2O:Hexane (40 mL, 1:1)	RT	0	0.5
**Transfer**			Module 1	Module 2		RT	250	5
**Mix**				Module 2		RT	500	30
**Filter**				Module 2		RT	250	5
**Dry**				Module 2		RT	0	1440

Table 5: Operations table for the execution of the cartridge synthesis of 3-methylphenyl MIDA boronate ester (compound **1**).

2.2. 3-bromophenylboronic acid

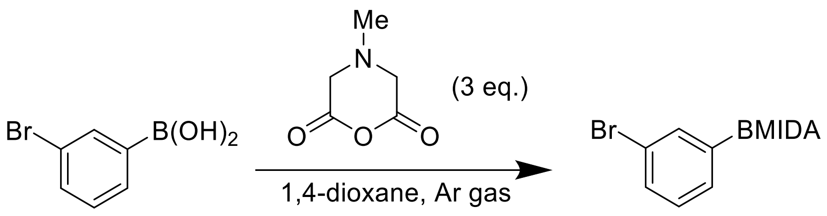

2

Scheme 3: synthetic scheme for the preparation of 3-bromophenyl MIDA boronic ester (compound **2**).

2.2.1. Glassware synthesis

3-Bromophenylboronic acid (136 mg, 1 mmol, 1 eq.) and MIDA anhydride (3 mmol, 3 eq.) was dissolved in dry dioxane (2.5 mL). The glass vial was capped and was flushed with argon gas using a balloon. The vial containing the mixture was uspended in a pre-heated 70 °C oil bath and stirred for 24 h. After stirring for named time, the mixture was allowed to cool to RT. Deionized water (18 µL, 1 mmol, 1 eq.) and dioxane (2.5 mL) was added to the solution and stirred for an hour. The mixture was transferred to a small round bottom flask and evaporated using a rotary evaporator. 5 mL of acetone was added to the flask under stirring, forming a suspension. Then the suspension was filtered and the filtrate collected. To the filtrate was added a Et_2O:hexane (1:1, 40 mL) mixture and stirred. A precipitate formed which was then filtered, dried in a desiccator under vacuum and the product, **2**, was collected as a white solid (135 mg, 43% yield).

^{1}H NMR (600.1 MHz; 303 K; DMSO; δ, ppm): 7.59-7.55 (2H, m), 7.44-7.42 (1H, tt), 7.34-7.33 (1H, t), 4.36-4.32 (2H, d), 4.17-4.12 (2H, d), 2.55 (3H, s).

^{13}C NMR (150 MHz; 303 K; DMSO; δ, ppm): 169.7, 135.4, 132.2, 131.9, 130.5, 122.4, 62.5, 48.2.

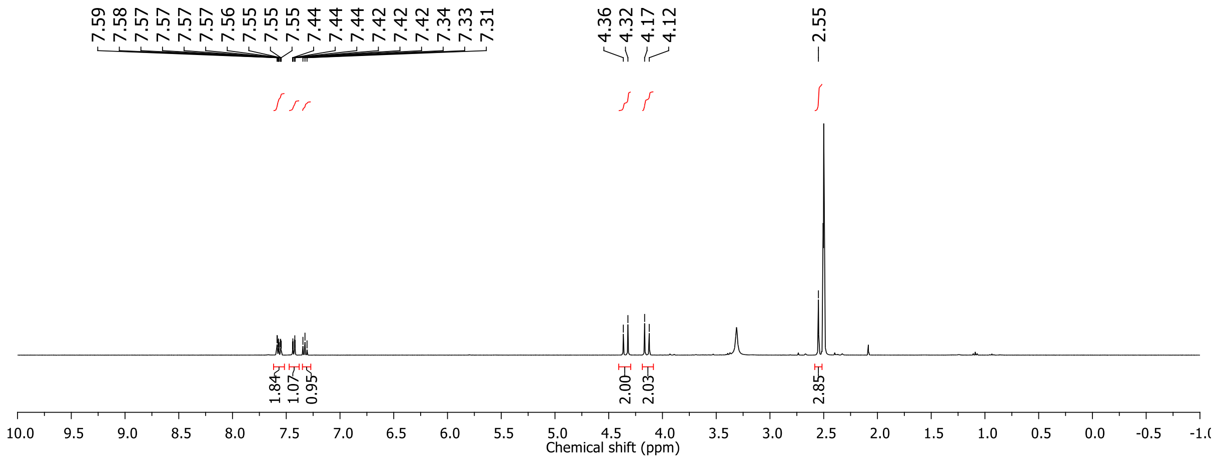

Figure 9: ^{1}H NMR of 3-bromophenylboronic acid MIDA ester made in glassware (compound **2**).

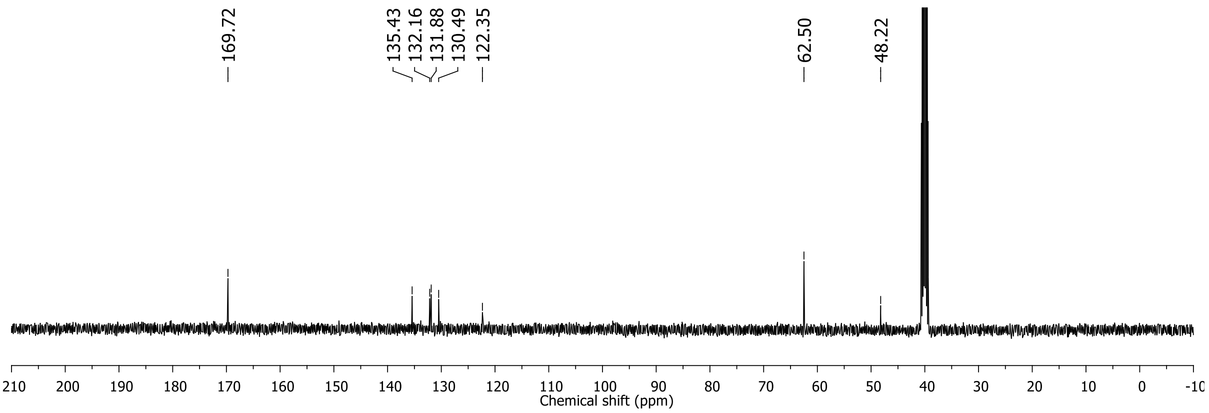

Figure 10: ^{13}C NMR of 3-bromophenylboronic acid MIDA ester made in glassware (compound **2**).

2.2.2. Reactionware synthesis

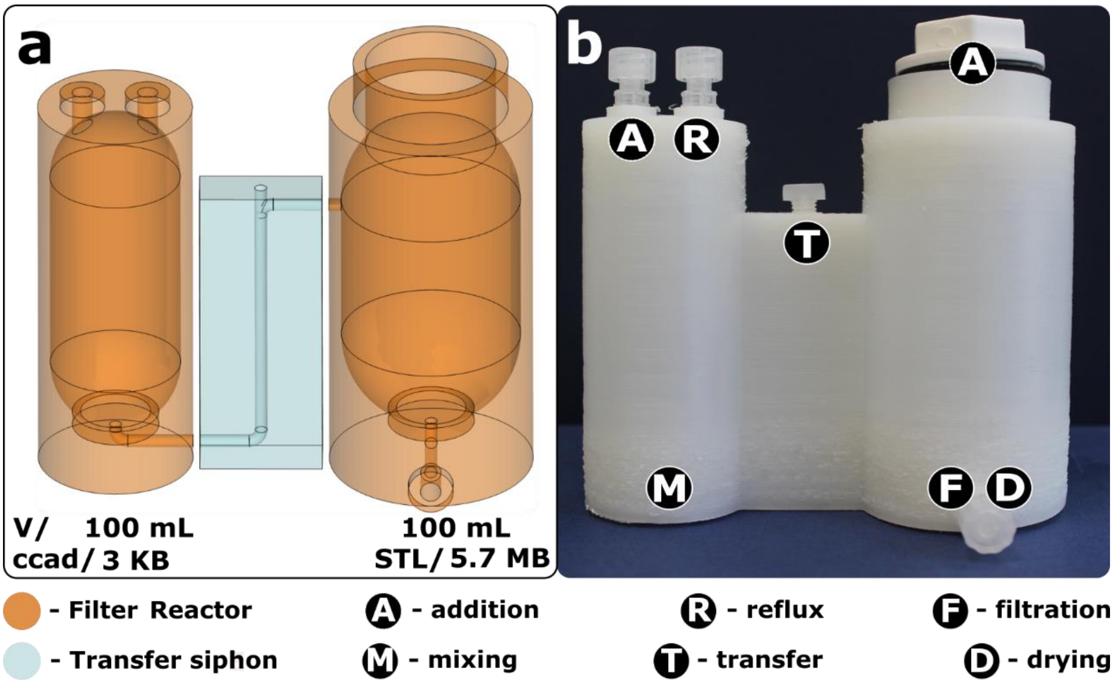

Figure 11: Reactionware for MIDA boronate ester formation, where **a** –transparent model with individual volumes of each Filter Reactor and associated file sizes, **b** – front view of real model, associated unit operations, their types and locations are highlighted (see legend).

3-Bromophenyl boronic acid (201 mg, 1 mmol, 1 eq.) and MIDA anhydride (387 mg, 3 mmol, 3 eq.) was charged to a flame-dried beaker along with anhydrous 1,4-dioxane (2.5 mL) and dissolved under stirring. Module 1 was flushed with a ballon of argon gas. The prepared mixture was transferred to Module 1 using a syringe. A 3D-printed high surface condenser was attached to Port 1, and all other ports were closed shut before lowering reactionware into a pre-heated oil bath (100 °C), where it was left to stir for 24 h. Post stirring, the mixture was allowed to cool to RT. H_2O (18 µL, 1 mmol, 1 eq.) and 1,4-dioxane (2.5 mL) was added to Module 1 and left to stir for 1 hour. Reactionware was lowered into a pre-heated oil-bath (70 °C), and a vacuum line was attached to Module 1 and the solvent partially evaporated (15 min, 50 mmHg), then dried more (10 min, 15 mmHg). Once dry, acetone (5

mL) was charged to Module 1 and the reaction was stirred for 20 minutes. A mixture of Et_2O:hexane (40 mL, 1:1) was added to Module 2. Reaction mixture was transferred from Module 1 to Module 2 whilst stirring in Module 2. Module 2 was left to stir for 30 minutes, whilst a white solid precipitated. A vacuum line was attached to the bottom port of Module 2 and a filtration was carried out. The product, **2**, was dried in reactionware overnight and isolated as a white powder (75 mg, 24% yield).

^{1}H NMR (600.1 MHz; 303 K; DMSO; δ, ppm): 7.58-7.56 (2H, m), 7.43-7.42 (1H, tt), 7.34-7.31 (1H, t), 4.35-4.33 (2H, d), 4.16-4.13 (2H, d), 2.55 (3H, s).

^{13}C NMR (150 MHz; 303 K; DMSO; δ, ppm): 169.3, 135.0, 132.7, 131.4, 130.1, 121.9, 62.0, 47.8.

ν_{max} (neat, cm^{-1}) 3010, 2964, 1757, 1552, 1465, 1451, 1434.

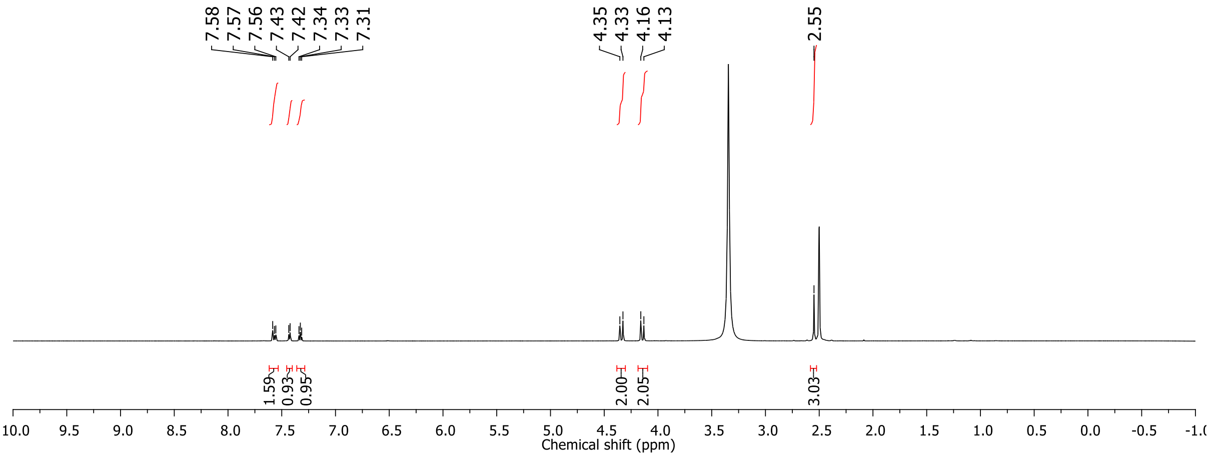

Figure 12: ^{1}H NMR of 3-bromophenyl MIDA boronic ester made in reactionware (compound **2**).

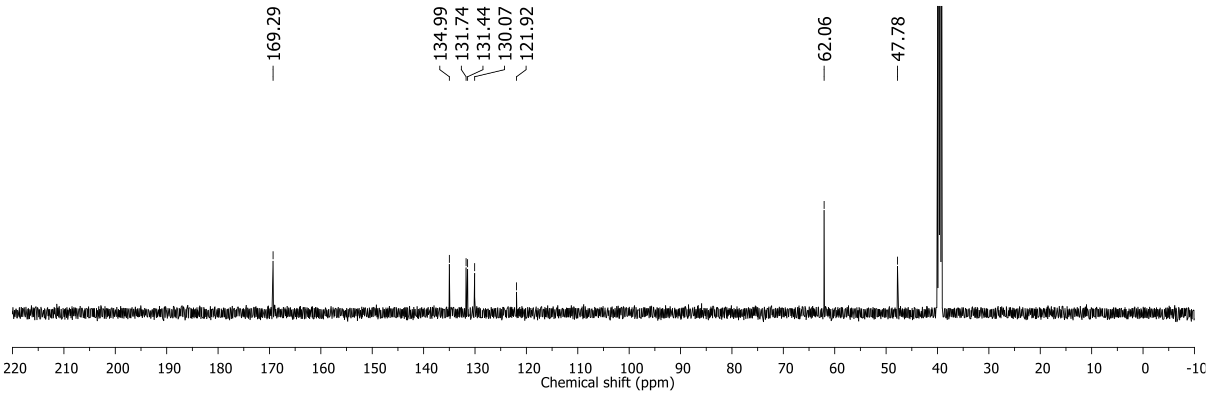

Figure 13: ^{13}C NMR of 3-bromophenyl MIDA boronic ester made in reactionware (compound **2**).

2.2.3. Operations Table

Operation	Icon	Reagent (g, mL)	From	To	Solvent (mL)	Temp (°C)	Stir Rate (RPM)	Time (min)
**Add**	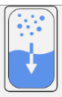	3-bromophenyl boronic acid (201 mg, 1 mmol, 1 eq.)	Spatula	Module 1		RT	0	0.5
**Add**	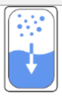	MIDA anhydride (387 mg, 3 mmol, 3 eq.)	Spatula	Module 1		RT	0	0.5
**Add**	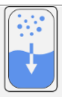		Syringe	Module 1	1,4-dioxane (2.5 mL)	RT	0	0.5
**Mix**	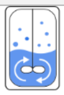			Module 1		RT	250	5
**Heat**	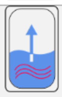			Module 1		100	250	1440
**Cool**	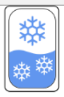			Module 1		RT	250	30
**Add**	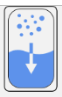	H_2O (18 µL, 1 mmol, 1 eq.)	Syringe	Module 1	1,4-dioxane (2.5 mL)	RT	250	5
**Mix**	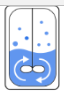			Module 1		RT	250	60
**Heat**	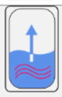			Module 1		70	250	15
**Dry**	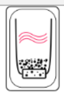			Module 1		70	250	10
**Add**	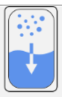		Syringe	Module 1	Acetone (5 mL)	RT	250	20
**Add**	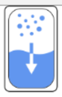		Syringe	Module 2	Et_2O:Hexane (40 mL, 1:1)	RT	0	0.5
**Transfer**	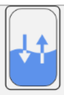		Module 1	Module 2		RT	250	5
**Mix**	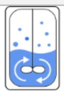			Module 2		RT	500	30
**Filter**	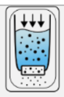			Module 2		RT	250	5
**Dry**	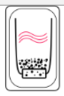			Module 2		RT	0	1440

Table 6: Operations table for the execution of the cartridge synthesis of 3-bromophenyl MIDA boronate ester (compound **2**).

2.3. Pentafluorophenylboronic acid MIDA ester

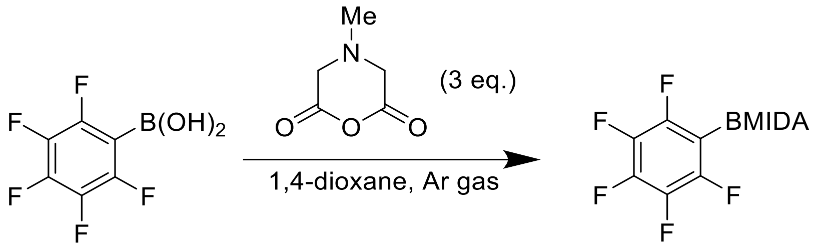

3

Scheme 4: synthetic scheme for the preparation of pentafluoro-phenyl MIDA boronic ester (compound **3**).

2.3.1. Glassware synthesis

A 250 mL three-necked round bottom flask was equipped with stir bar, a waterless condenser (CondenSyn) and connected to a Schlenk line. Rubber septa were added to the two open ports and the system placed under vaccum. Once under vaccum, the set-up was flame-dried using a propane gas cannister to remove any residual moisture. After this, the set-up was purged with N_2 using a three time vaccum/N_2 purge cycle, leaving the system under an inert atmosphere. To the flask, pentafluorophenyl boronic acid (1 mmol, 211 mg) and MIDA anhydride (3 eq, 387 mg) was added and the purge cycle repeated after drying the set-up under vaccum prior to purging. Using an Ar balloon, 1,4-dioxane (7.5 mL) was added to the flask under vigorous stirring. The flask was then lowered into a pre-heated aluminium heating block (DrySyn) at 70 °C. The solution was stirred for 24 hr. After this time had elapsed, the heating block was removed and the flask allowed to cool to room temperature. Once cooled, deionised water (1 mmol, 18 µL) was added to the flask and stirred for 1 hr. Following this, the solution was evaporated to dryness using a rotary evaporator. To the off-white solid, acetone (5 mL) was added and the solution stirred for 20 mins, causing a precipitate to form. The precipitate was filtered off (recovered MIDA acid) and the filtrate added to a vigorously stirred solution of Et_2O:Hexane (1:1, 120 mL). This mixture was stirred for 1 hr, leading to precipitation of the final MIDA ester product, **3** (194mg, **60 %** yield, 97% purity).

^{1}H NMR (400.2 MHz; 303 K; DMSO; δ, ppm): 4.47-4.42 (2H, d), 4.19-4.15 (2H, d), 2.73 (3H, s).

^{13}C NMR (100.6 MHz; 303 K; DMSO; δ, ppm): 168.9, 62.6, 57.3, 47.7.

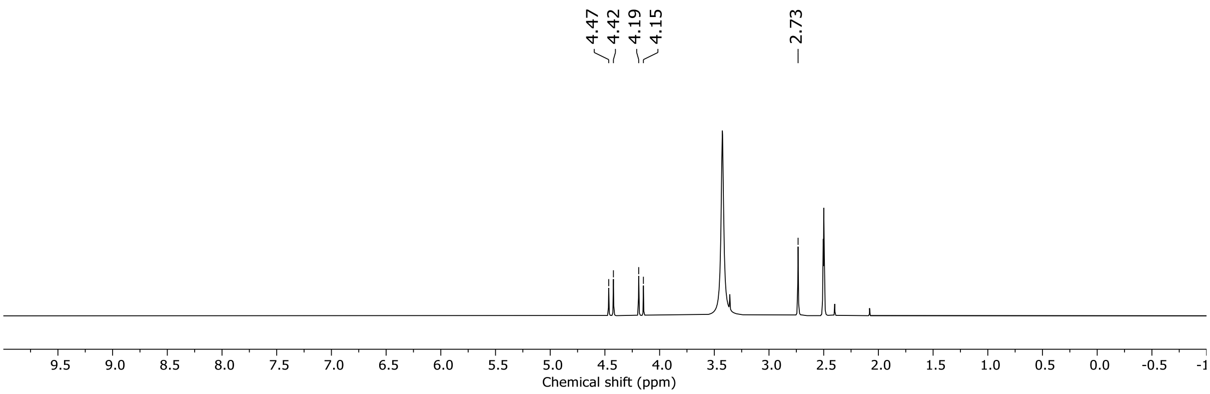

Figure 14: ^{1}H NMR of pentafluorophenylboronic acid MIDA ester made in glassware (compound **3**).

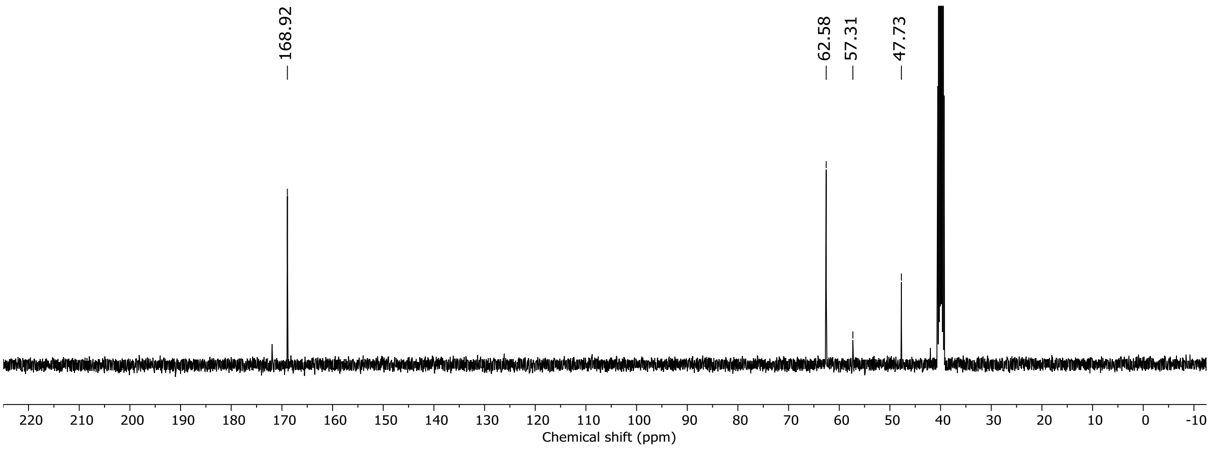

Figure 15: ^{13}C NMR of pentafluorophenylboronic acid MIDA ester made in glassware (compound **3**).

2.3.2. Reactionware synthesis

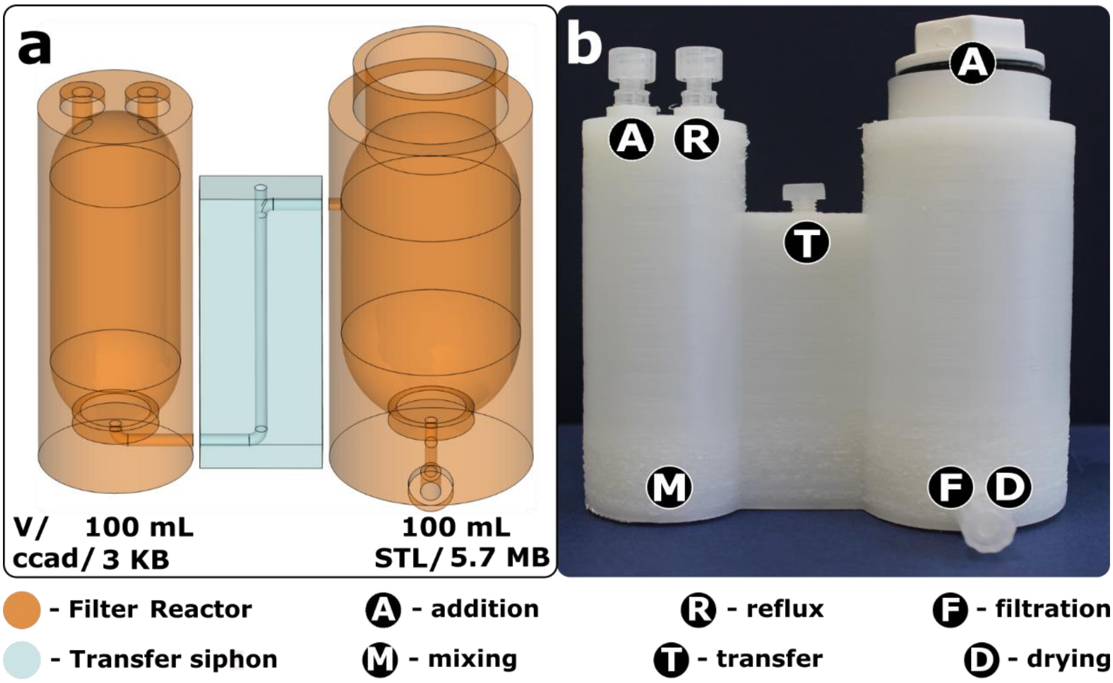

Figure 16: Reactionware for MIDA boronate ester formation, where **a** –transparent model with individual volumes of each Filter Reactor and associated file sizes, **b** – front view of real model, associated unit operations, their types and locations are highlighted (see legend).

The monolith here consisted of two filter reactors (**M1** and **M2**) connected via a cannula. **M1** has two drilled Luer inputs on top to allow reagent addition. The cannula has a drilled hole to fit a threaded PP screw which can aid with nitrogen flow allowing for transfer of material from **M1** to **M2**. **M2** is an open filter reactor, with a threaded screw cap allowing for sealed conditions to be maintained during work-up and filtration/evaporation. Once stir bars had been added to both chambers and all ports were sealed, an Ar ballon was attached to one of the Luer inlets on top of **M1** and was flushed completely to remove any air, before sealing the ports with screw tops. In a separate flame-dried 100 mL glass beaker equipped with a stir bar, pentafluorophenyl boronic acid (1 mmol, 211 mg) and MIDA anhydride (3 eq, 387 mg) was added, along with anhydrous 1,4-dioxane (7.5 mL) and the reaction mixture was stirred for 10 min, until all reagents were dissolved. The solution was added directly to **M1** using a syringe before closing the ports as above. The monolith was lowered into a pre-heated 90 °C silica oil bath and was heated for 20 hours. After this time had elapsed, the monolith was removed from the oil bath and allowed to cool completely to room temperature (approx. 30 mins). To **M1**, deionised water (1 mmol, 18 µL) was added and the mixture allowed to stir for 1 hr. After this, a vaccum line adapter was attached to one of the Luer inlets on top of **M1** and the mixture was dried overnight at 50 °C (70 °C oil bath) at 50 mmHg. Then, the vaccum line was removed and acetone (5 mL) was added to **M1**, and stirred vigorously for 20 min. Now, using a nitrogen line attached to **M1**, the reaction mixture was transferred into **M2** (using the screw to control rate of transfer) which contained a 1:1 mixture of Et_2O:Hexane (40 mL). Once fully transferred, any open ports on **M1** were closed and the reaction mixture stirred for 30 mins to precipitate the desired MIDA ester product, **3**, which was dried under vacuum overnight (97mg, **30%** yield, purity 97%).

^{1}H NMR (600.1 MHz; 303 K; DMSO; δ, ppm): 4.45 (d, *J* = 17.3 Hz, 2H), 4.18 (d, *J* = 17.3 Hz, 2H), 2.74 (s, 3H).

^{13}C NMR (150 MHz; 303 K; DMSO; δ, ppm): 168.38, 61.96, 47.08.

ν_{max} (neat, cm^{-1}) 3008, 2966, 1758, 1651, 1525, 1467, 1423, 1392.

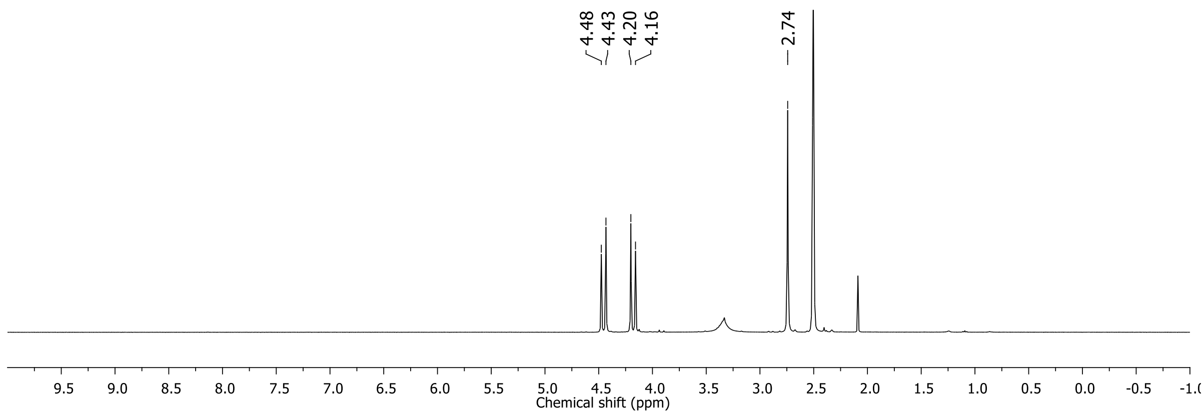

Figure 17: ^{1}H NMR of pentafluorophenylboronic acid MIDA ester made in reactionware (compound **3**).

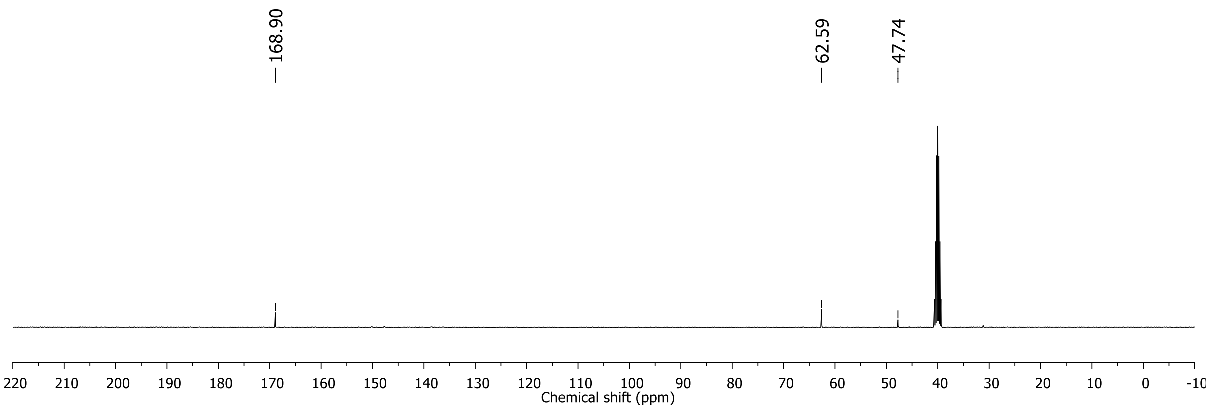

Figure 18: ^{13}C NMR of pentafluorophenylboronic acid MIDA ester made in reactionware (compound **3**).

2.3.3. Operations Table

Operation	Icon	Reagent (g, mL)	From	To	Solvent (mL)	Temp (°C)	Stir Rate (RPM)	Time (min)
**Add**		pentafuluorophenyl boronic acid (211 mg, 1 mmol, 1 eq.)	Spatula	Module 1		RT	0	0.5
**Add**		MIDA anhydride (387 mg, 3 mmol, 3 eq.)	Spatula	Module 1		RT	0	0.5
**Add**			Syringe	Module 1	1,4-dioxane (2.5 mL)	RT	0	0.5
**Mix**				Module 1		RT	250	5
**Heat**				Module 1		100	250	1440
**Cool**				Module 1		RT	250	30
**Add**		H_2O (18 µL, 1 mmol, 1 eq.)	Syringe	Module 1	1,4-dioxane (2.5 mL)	RT	250	5
**Mix**				Module 1		RT	250	60
**Heat**				Module 1		70	250	15
**Dry**				Module 1		70	250	10
**Add**			Syringe	Module 1	Acetone (5 mL)	RT	250	20
**Add**			Syringe	Module 2	Et_2O:Hexane (40 mL, 1:1)	RT	0	0.5
**Transfer**			Module 1	Module 2		RT	250	5
**Mix**				Module 2		RT	500	30
**Filter**				Module 2		RT	250	5
**Dry**				Module 2		RT	0	1440

Table 7: Operations table for the execution of the cartridge synthesis of pentafluorophenyl MIDA boronate ester (compound **3**).

3. Ester hydrolysis

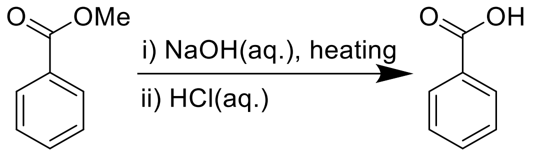

4

Scheme 5: Ester hydrolysis, benzoic acid synthesis (compound **4**).

3.1. Glassware synthesis

The glassware procedure is based on a manual from a University of Glasgow undergraduate chemistry course "Synthesis 1" (2019) – Experiment 5: Carboxylic Acid and Ester Functional Groups. Scanned experiment pages can be found in the appendix. It was repeated accordingly, using methyl benzoate as the ester. The final product, benzoic acid, was afforded as a white powder (357 mg, **51%**) (**c**ompound **4**).

3.2. Reactionware synthesis

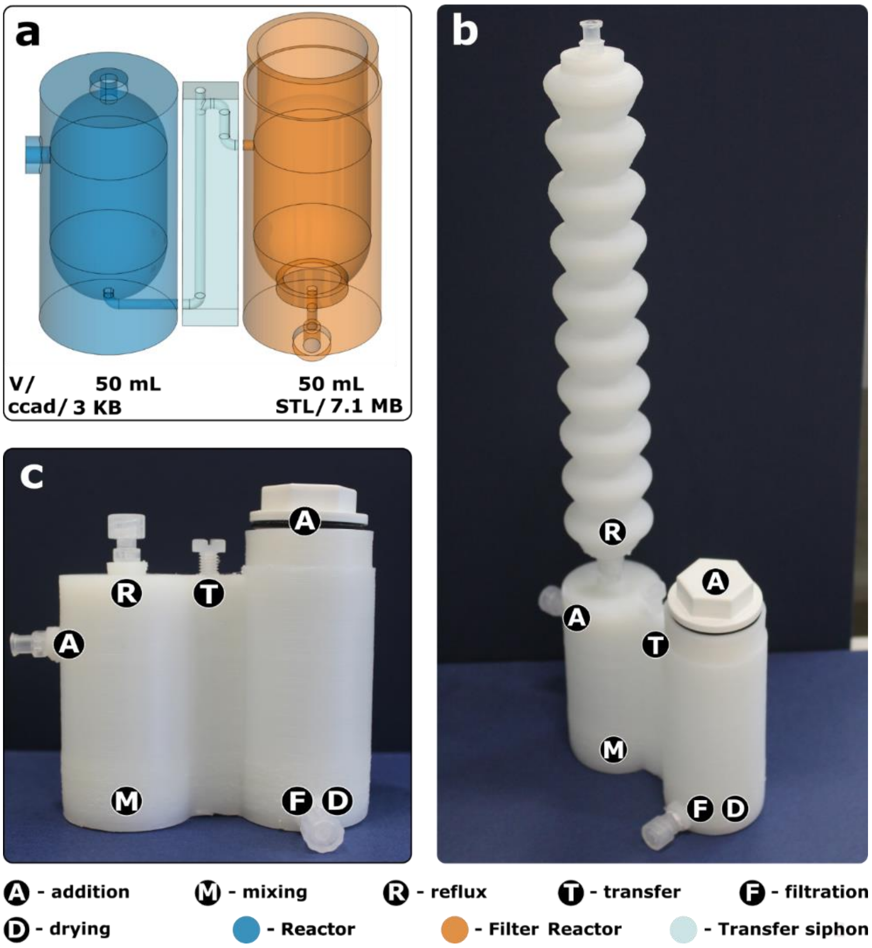

Figure 19: Reactionware for ester hydrolysis, where **a** –transparent model with individual volumes of each reactor and associated file sizes, **b** – isometric view of the real model, with a 3D-printed high surface condenser attached, **c** – front view of real model without the condenser. Associated unit operations, their types and locations are highlighted (see legend).

A Pasteur pipette was used to add methyl benzoate (20 drops, 424 mg) into Module 1 keeping both ports 1 and 2 open. $NaOH_{(aq.)}$ (15 mL, 5 M) and EtOH (3 mL) was added to Module 1 *via* syringe. Port 2 was equipped with 3D-printed polypropylene reflux condenser and port 1 was closed. The reactionware monolith was submerged in an oil bath (T = 110 °C) and left to reflux for 1 hour. After the 1 hour period, the monolith was raised out of the oil bath and the leftover oil was wiped from the sides of the monolith. After allowing the mixture the mixture to cool for 5 minutes $HCl_{(aq.)}$ (15 mL, 5 M) was through port 2 *via* syringe with a small stirrer bar. The mixture was stirred for 10 minutes. The mixture was transferred from Module 1 to Module 2 by opening ports 2 and 3 along with the screw valve and applying pressure of compressed air through port 2. The monolith was submerged into an ice bath and left for 30 minutes before carrying out a filtration at Module 2 by applying vacuum to port 4. After allowing the mixture to dry under vacuum, the product, **4** was afforded as a white powder (230 mg, **58%**). ^{1}H NMR (600.1 MHz; 303 K; DMSO; δ, ppm; J, Hz): 7.96 -7.93 (2H; m), 7.62-7.58 (1H; tt; 8.2), 7.51-7.46 (2H; m).

v_{max} (neat, cm^{-1}) 3071, 3009, 2827, 2667, 2552, 1745, 1678, 1600, 1583.

The reaction completion can be monitored by ^{1}H NMR by comparing a sample of the starting material with a sample of isolated product. The disappearance of the methyl group signal can be seen.

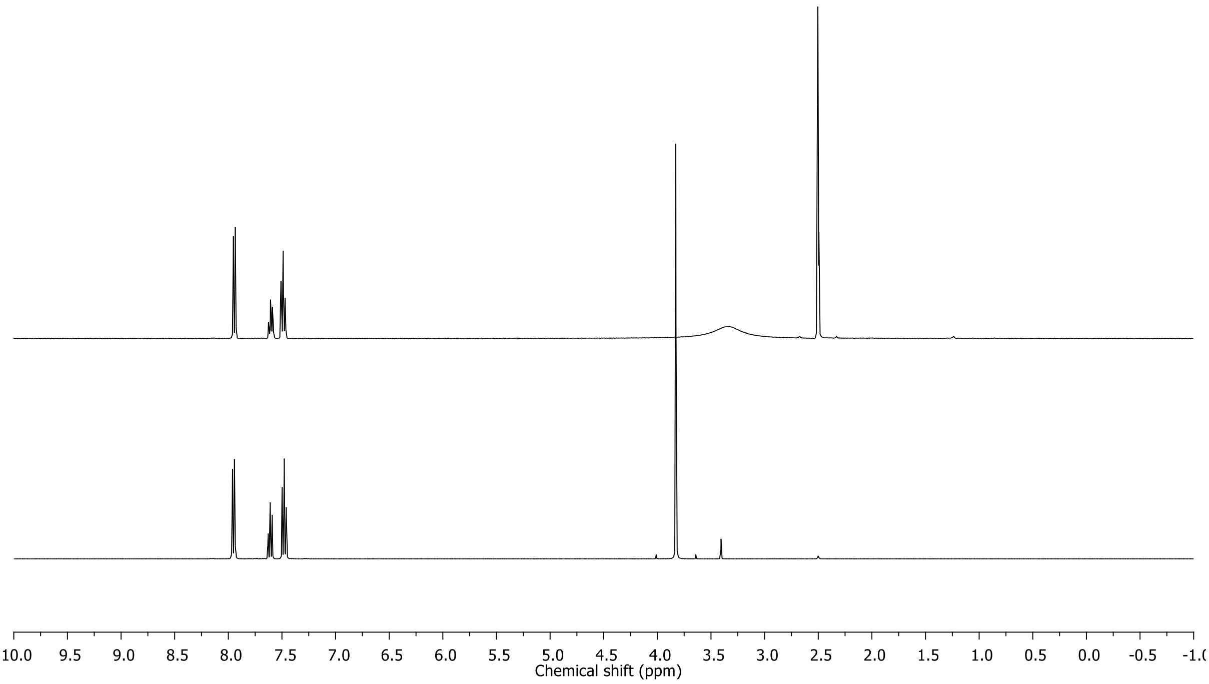

Figure 20: ^{1}H NMR featuring the disapperance of a $-CH_3$ signal.

3.2.1. Operations Table

Operation	Icon	Reagent (g, mL)	From	To	Solvent (mL)	Temp (°C)	Stir Rate (RPM)	Time (min)	Hazard
**Add**	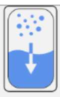	Methyl benzoate (424 mg)	Syringe	Module 1		RT	0	0	
**Add**	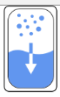	5M NaOH (15 mL)	Syringe	Module 1	EtOH (3 mL)	RT	0	0.5	Corrosive
**Heat**	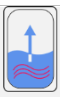			Module 1		110	250	60	
**Cool**	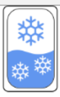			Module 1		0	250	5	
**Add**	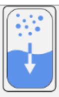	5M HCl (15 mL, pH 1)		Module 1		RT	250	0.5	Corrosive
**Mix**	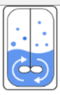			Module 1		RT	250	10	
**Transfer**	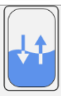		Module 1	Module 2		RT	250	0.5	
**Cool**	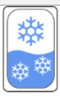			Module 2		0	250	30	
**Filter**	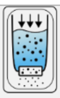			Module 2		RT	250	5	
**Dry**	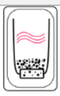	Methyl benzoate (424 mg)		Module 2		RT	0	1440	

Table 8: Operations table for the execution of the cartridge synthesis of benzoic acid (compound **4**).

4. Wittig olefination

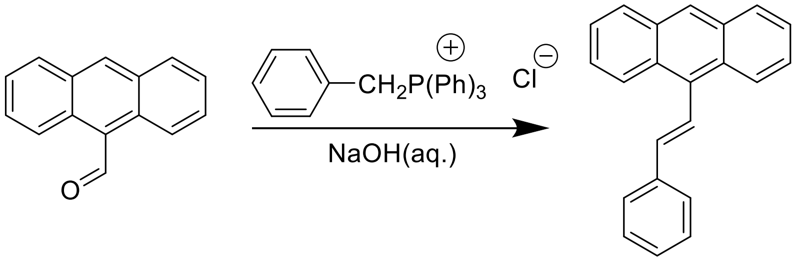

5

Scheme 6: Synthesis scheme for the Wittig reaction using 9-anthraldehyde (compound **5**).

The glassware synthesis is an adaptation from a known literature procedure.[2] The scale has been increased three-fold for easier handling in reactionware. The final recrystallization solvent was replaced from 2-propanol to 1-propanol as it has a lower boiling point and thus is more convenient to use in reactionware.

4.1. Glassware synthesis

The following was placed in a 10 mL conical flask: 9-anthraldehyde (0.345 g, 1.7 mmol); benzyltriphenylphosphonium chloride (0.6 g, 1.8 mmol); DMF (1.5 mL); medium size stirrer bar. The mixture was stirred vigorously before 50% $NaOH_{(aq.)}$ (0.75 mL) was added using a micropipette. The reaction mixture was stirred for 1 hour and after that time period 2-propanol/H_2O (12 mL, 1:1) was added. The reaction mixture was stirred for a further 10 minutes as a yellow precipitate formed. The reaction mixture was filtered using a Buchner funnel and dried for 10 minutes. The yellow powder was transferred to a 25 mL conical flask and 2-propanol (12 mL) was added, the mixture was heated to boiling point in a water bath until all of the solid dissolved. The clear yellow solution was let to cool to room temperature as the product, **2**, crystallized as yellow crystals (0.187 g; 0.65 mmol; **40 %**). ^{1}H and ^{13}C NMR spectra were identical to that of literature and that of the product prepared in reactionware in the next section.[2]

4.2. Reactionware synthesis

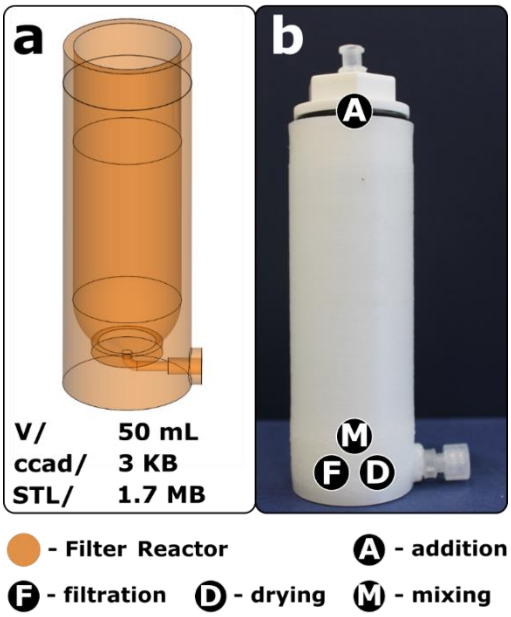

Figure 21: Reactionware for the Wittig olefination reaction, where **a** –transparent model with individual volumes of the Filter Reactor and associated file sizes, **b** – front view of real model. Associated unit operations, their types and locations are highlighted (see legend).

9-Anthraldehyde (0.345 g, 1.7 mmol), benzyltriphenylphosphonium chloride (0.6 g, 1.8 mmol) and DMF (1.5 mL) were introduced into the cylindrical polypropylene filter reactor reactionware vessel with a stirrer bar. With stirring, 50% $NaOH_{(aq.)}$ (0.75 mL) was added using a pipette dropwise. The reaction mixture was allowed to stir for 1 hour. Afterward, 2-propanol/H_2O (12 mL, 1:1) was added and the mixture was further stired for 10 minutes. A vacuum line with a solvent trap was attached to port 2 and the mixture was filtered. The vacuum line was detached and port 2 closed. An oil bath was heated up to 115 °C. 2-propanol (12 mL) was added to the vessel and it was submerged into the oil bath just above the solvent front. Port 1 was closed and a 3D printed reflux condenser was attached to Port 1_{aux}.. The mixture was heated to 95 °C with stirring until the contents were dissolved (about 30 min). The reactionware vessel was then taken out of the oil bath and let to cool down to room temperature. As the mixture cooled down the product, **2**, crystallized as yellow plates and was collected by filtration (0.182 g, **39%**).

^{1}H NMR (400.13 MHz; $CDCl_3$): 8.39 (1H; s), 8.37-8.33 (2H; m), 8.03-7.98 (2H; m), 7.93-7.89 (1H; d; J=16.6 Hz, vynilic), 7.68-7.66 (2H, m), 7.49-7.42 (6H, m), 7.37-7.32 (1H, m), 6.96-6.92 (1H, d, J=16.6 Hz, vinylic);

^{13}C{^{1}H} NMR (400.13 MHz, $CDCl_3$): 137.4, 132.8, 131.5, 129.8, 128.9, 128.7, 128.0, 126.6, 126.5, 126.0, 125.5, 125.2, 124.9.

ν_{max} (neat, cm^{-1}) 3024, 1620, 1516, 1493, 1448, 1409, 1385.

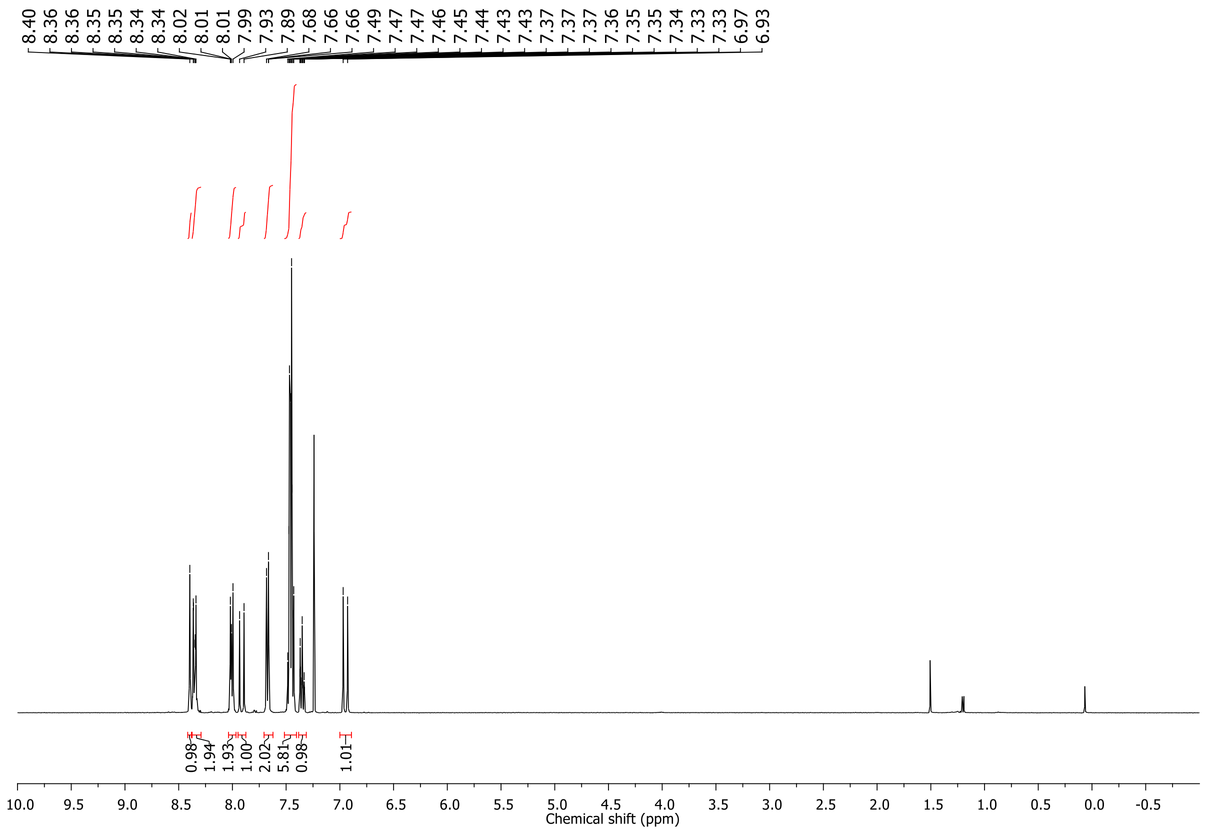

Figure 22: ^{1}H NMR of the Wittig reaction product (compound **5**).

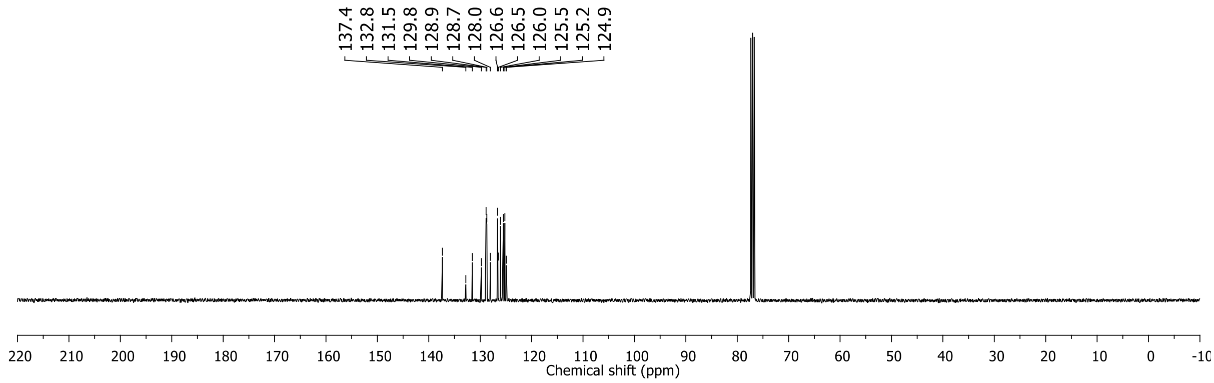

Figure 23: ^{13}C NMR of the Wittig reaction product (compound **5**).

4.2.1. Operations Table

Operation	Icon	Reagent (g, mL)	From	To	Solvent (mL)	Temp (°C)	Stir Rate (RPM)	Time (min)	Hazard
**Add**	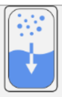	9-anthraldehyde (0.345 g, 1.7 mmol)	Spatula	Module 1		RT	0	0.5	
**Add**	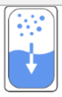	benzyltriphenylphosphonium chloride (0.6 g, 1.8 mmol)	Spatula	Module 1		RT	0	0.5	
**Add**	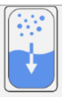		Syringe	Module 1	DMF (1.5 mL)	RT	0	0.5	
**Mix**	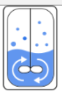			Module 1		RT	250	5	
**Add**	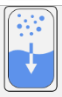	50% $NaOH_{(aq.)}$ (0.75 mL)	Syringe	Module 1		RT	250	60	Corrosive
**Add**	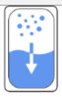		Syringe	Module 1	2-propanol/H_2O (12 mL, 1:1)	RT	250	0.5	
**Mix**	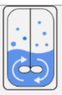			Module 1		RT	250	10	
**Filter**	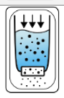			Module 1		RT	250	5	
**Add**	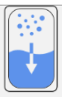		Syringe	Module 1	2-propanol (12 mL)	RT	250	0.5	
**Heat**	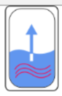			Module 1		95	250	30	
**Cool**	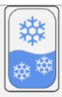			Module 1		RT	0	30	
**Filter**	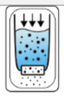			Module 1		RT	250	0.5	

Table 9: Operations table for the execution of the cartridge synthesis of trans-9-(2-Phenylethenyl)anthracene (compound **5**).

5. Suzuki coupling

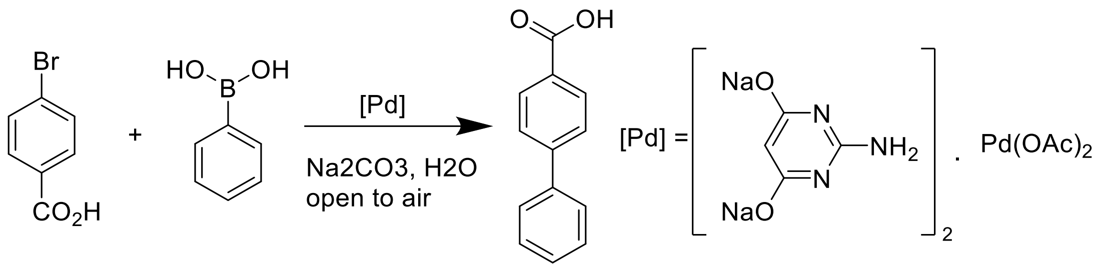

6

Scheme 7: synthesis scheme used for Suzuki coupling (compound **6**).

5.1. Method 1

5.1.1. Glassware synthesis

4-Bromobenzoic acid (0.5 g) and phenylboronic acid (0.37 g) were loaded to a 100 mL flask along with a stir bar. $Na_2CO_{3\,(aq.)}$ (0.8 g in 15 mL H_2O) was prepared in a separate beaker. The aqueous carbonate solution was added to the flask containing the bromobenzoic and phenylboronic acids and stirred for 10 minutes until everything was dissolved. In the mean time, 1 mL of previously prepared [Pd] catalyst (see [Pd] catalyst preparation section) was measured using a micro pipette and added to the 100 mL flask. The mixture was heated to 70 °C in a sand bath (set to 80 °C) and left to stir for 30 minutes. After cooling,the mixture was allowed to cool to RT and then submerged in an ice bath for 20 minutes. 1 M HCl (25 mL) was added very slowly with vigorous stirring to the flask. The mixture was stirred for another 5 minutes after the acid was added. The mixture was filtered using a Buchner funnel. The residue was washed with 5 mL H_2O and dried for 2 minutes. The product was recrystallized from a 1 M HCl (4 mL) and EtOH (20 mL) mixture which was heated to 70 °C. Afterwards, the mixture was cooled to room temperature, followed a submersion in an ice bath for 20 minutes. The mixture was filtered and the product, **6**, was isolated as a white powder (287 mg, 57% yield).

^{1}H NMR (600.1 MHz; 303 K; DMSO; δ, ppm): 12.97 (1H, s), 8.03-8.02 (2H, d), 7.81-7.79 (2H, d), 7.74-7.73 (2H, d), 7.52-7.49 (2H, t), 7.44-7.41 (1H, t).

^{13}C NMR (150 MHz; 303 K; DMSO; δ, ppm): 167.2, 144.4, 139.1, 130.0, 129.7, 129.1, 128.3, 127.0, 126.9.

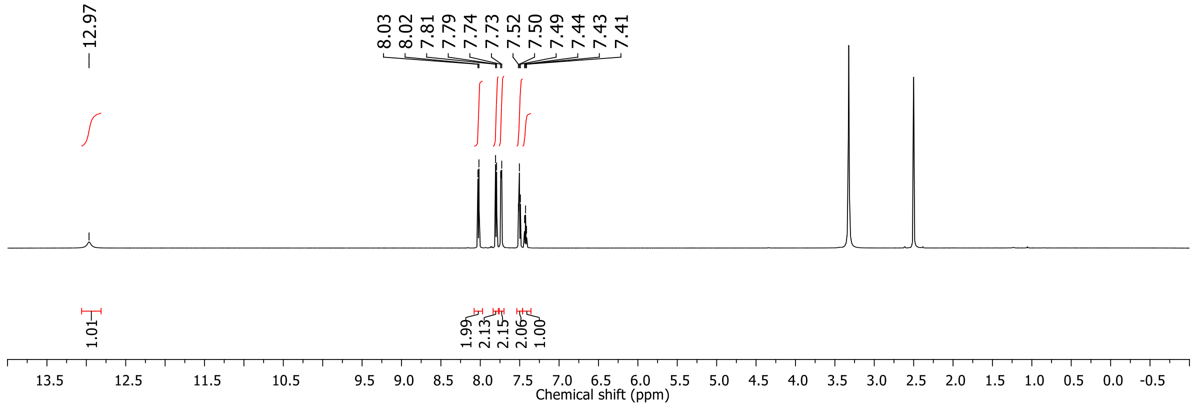

Figure 24: ^{1}H NMR of compound **6** synthesised using method 1 in glassware.

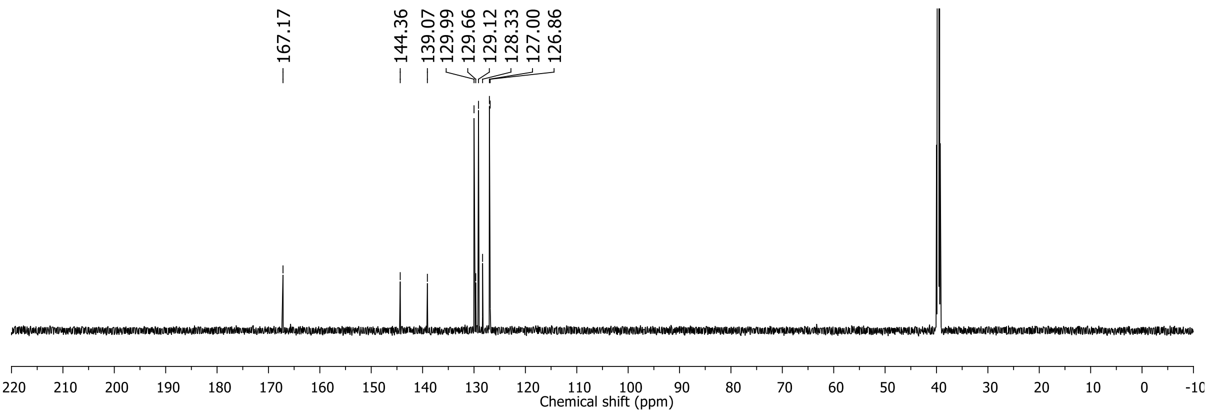

Figure 25: ^{13}C NMR of compound **6** synthesised using method 1 in glassware.

5.1.2. Reactionware synthesis

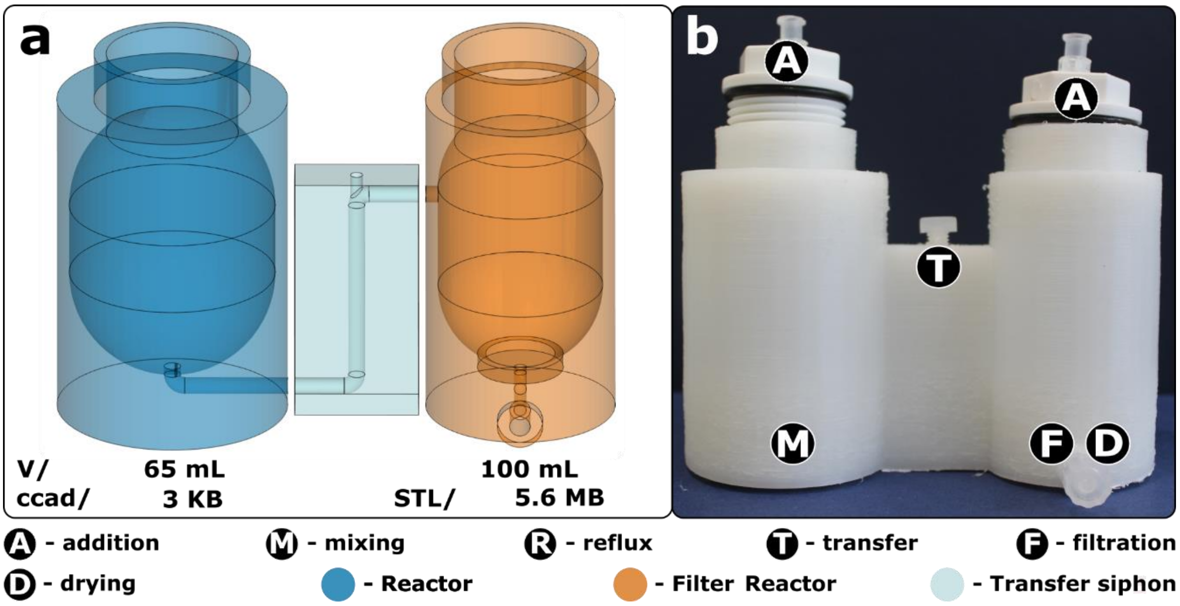

Figure 26: Reactionware for the Suzuki-Miyaura reaction, **a** – transparent model with individual volumes of reactors and associated file sizes, **b** – front view of real model, associated unit operations, their types and locations are highlighted (see legend).

4-Bromobenzoic acid (0.5 g) and phenylboronic acid (0.37 g) were loaded to Module 1 along with a stir bar. Na_2CO_3 (0.8 g in 15 mL H_2O) was added to Module 1 as a solution. The mixture was stirred for 10 minutes, 1 mL of previously prepared [Pd] catalyst was added to Module 1. The mixture was heated to 70 °C in a sand bath (set to 90 °C) and left to stir for 30 minutes. After cooling the mixture to RT, reactionware was submerged in an ice bath. 1 M HCl (25 mL) was slowly added to Module 1 with stirring. The mixture was stirred for another 5 minutes after the acid was added. The mixture was transferred from Module 1 to Module 2 where a filtration was carried out. The residue was washed with 5 mL H_2O and dried for 2 minutes under vacuum. 1 M HCl (4 mL), EtOH (20 mL) and a stir bar was added to Module 2 and the mixture was heated to 70 °C in a water bath. Afterwards, the mixture was cooled to room temperature, followed a submersion in an ice bath for 20 minutes. The mixture was filtered and the product was isolated as a white powder. The product, **6**, was then dried thoroughly overnight under vaccum (163 mg, 33% yield).

^{1}H NMR (600.1 MHz; 303 K; DMSO; δ, ppm): 12.96 (broad H-OR), 8.03-8.02 (2H, d), 7.80-7.79 (2H, d), 7.73-7.72 (2H, d), 7.51-7.49 (2H, t), 7.43-7.41 (1H, t).

^{13}C NMR (150 MHz; 303 K; DMSO; δ, ppm): 167.2, 144.4, 139.1, 130.0, 129.7, 129.1, 128.3, 127.0, 126.9.

ν_{max} (neat, cm^{-1}) 2807, 2545, 1672, 1607, 1582, 1561, 1518.

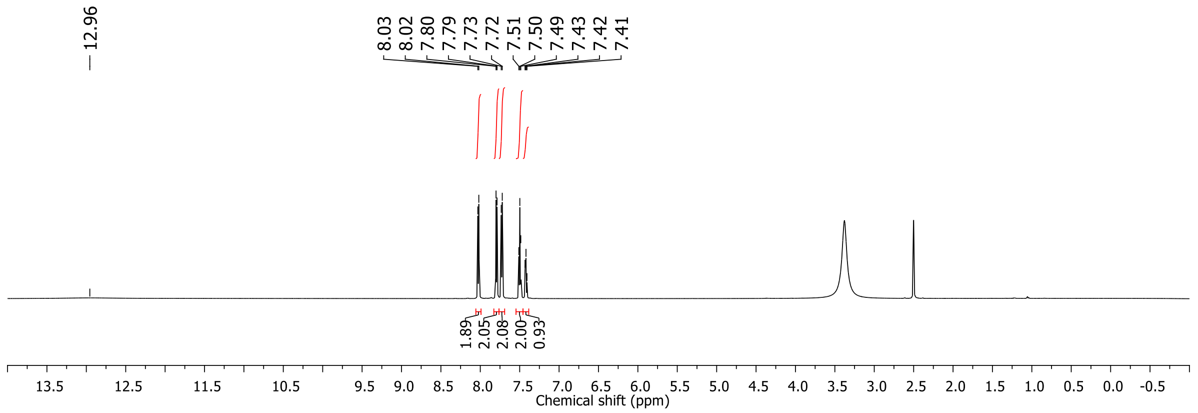

Figure 27: ^{1}H NMR of compound **6** synthesised using method 1 in reactionware.

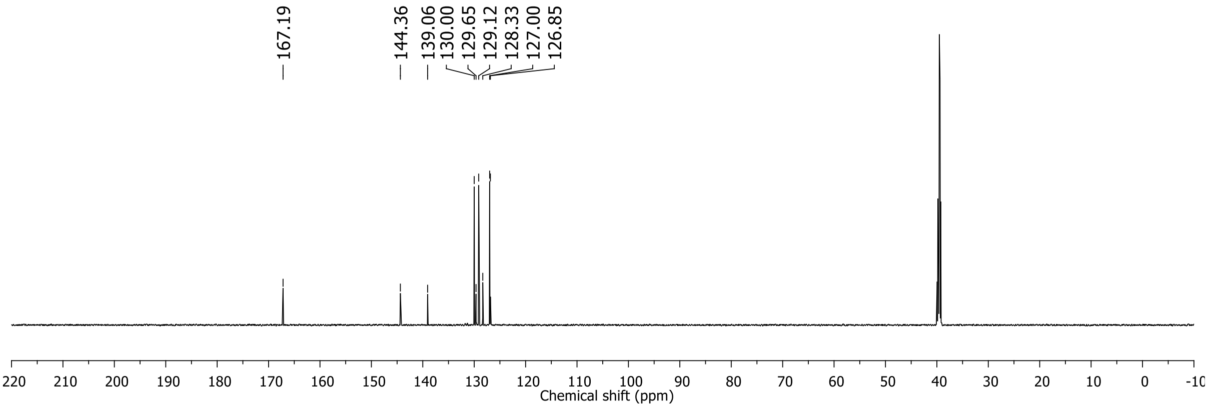

Figure 28: ^{13}C NMR of the compound **6** synthesised using method 1 in reactionware.

5.1.1. Operations Table

Operation	Icon	Reagent (g, mL)	From	To	Solvent (mL)	Temp (°C)	Stir Rate (RPM)	Time (min)	Hazard
**Add**		4-bromobenzoic acid (0.5 g, 2.5 mmol)	Spatula	Module 1		RT	0	0.5	
**Add**		phenylboronic acid (0.37 g, 3.0 mmol)	Spatula	Module 1		RT	0	0.5	
**Add**			Syringe	Module 1	sodium carbonate (0.80 g in 15 mL water, 7.5 mmol)	RT	0	0.5	
**Mix**				Module 1		RT	250	10	
**Heat**				Module 1		90	250	30	
**Add**				Module 1	palladium catalyst stock solution (1.0 mL, 0.25 mM)	90	250	0.5	
**Mix**				Module 1		90	250	30	
**Cool**				Module 1		RT	250	30	
**Add**		1 M HCl (25 mL)		Module 1		RT	500	10	Corrosive, slow addition*
**Mix**				Module 1		RT	500	5	
**Transfer**			Module 1	Module 2		RT	500	5	
**Filter**				Module 2		RT	500	0.5	
**Add**				Module 2	5 mL H_2O	RT	250	0.5	
**Add**		1 M HCl (4 mL)		Module 2		RT	250	0.5	Corrosive
**Add**				Module 2	EtOH (20 mL)	RT	250	0.5	
**Mix**				Module 2		70	250	30	

**Cool**	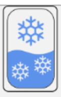			Module 2		RT	250	20	
**Filter**	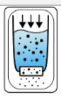			Module 2		RT	250	5	
**Dry**	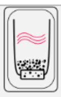			Module 2		RT	250	1440	

Table 10: Operations table for the execution of the cartridge synthesis of 4-phenyl benzoic acid. ***Slow addition is required to prevent uncontrolled effervescence.**

5.2. Method 2

5.2.1. Glassware synthesis

4-Bromobenzoic acid (0.50 g, 2.5 mmol) was loaded into a 250 mL Erlenmeyer flask containing a stir bar, followed by phenylboronic acid (0.37 g, 3.0 mmol). In a separate 50 mL beaker, sodium carbonate (0.80 g, 7.5 mmol) was added, followed by 15 mL of deionized water. This solution was stirred for 10 minutes at room temperature until full dissolution was observed. Once dissolved, this solution was added directly into the Erlenmeyer flask and stirred vigoursoly for 10 minutes at room temperature until full dissolution was observed. Once all the reactants were dissolved, the flask was lowered into a pre-heated water bath and heated to 70 ºC. When this temperature was reached, the palladium catalyst solution (1.0 mL, 0.25 mM) was added via a syringe and the reaction mixture stirred for 30 mins at 70 ºC. Upon of the catalyst, the solution appeared yellow in colour. After stirring for 10 mins, the product appeared to precipate as a white solid. The resultant mixture was further stirred for 20 mins to ensure reaction completion. Once this time had elapsed, the flask was removed from the heat and left to cool to room temperature for 30 mins. Once cooled, the flask was placed directly into an ice bath and with stirring hydrochloric acid (25 mL, 1 M) was slowly added into the flask using a pipette. (Caution! The HCl must be added dropwise! The HCl reacts with the excess carbonate to generate carbon dioxide. If the HCl is added too quickly, the solution may foam over the reaction vessel.) Once all added, the resultant mixture was stirred for 5 mins. The crude product was isolated by vaccum filtration, the flask was washed with 5 mL of deionised water, and the resulting white solid dried on the filter for 10 mins. To obtain purified product, the solid was added into a clean, dry 150 mL Erlenmeyer flask equipped with a stir bar. To the flask, 10 mL of EtOH and 20 mL of deionised water were added. With stirring, hydrochloric acid (5 mL, 1 M) was added slowly using a pipette. The resultant mixture was stirred at room temperature to precipate the purified product as a white crystalline solid. The product was vaccum filtered and washed twice with 5 mL of ice-cold ethanol to remove any remaining impurities. The product, **6**, was dried under vaccum in a dessicator overnight. The mass of the product, **6**, obtained was (0.433 g, 2.18 mmol), pure yield 83 %, purity 95% by ESI(-) LC-MS.

^{1}H NMR (600.1 MHz; 303 K; DMSO; δ, ppm; J, Hz): 12.98 (broad <u>H</u>O-R), 8.04-8.03 (2H, d), 7.79-7.78 (2H, d), 7.72-7.71 (2H, d), 7.50-7.48 (2H, t), 7.42-7.40 (1H, t).

^{13}C NMR (150 MHz; 303 K; DMSO; δ, ppm; J, Hz): 167.2, 144.4, 139.1, 130.0, 129.7, 129.1, 128.3, 127.0, 126.8.

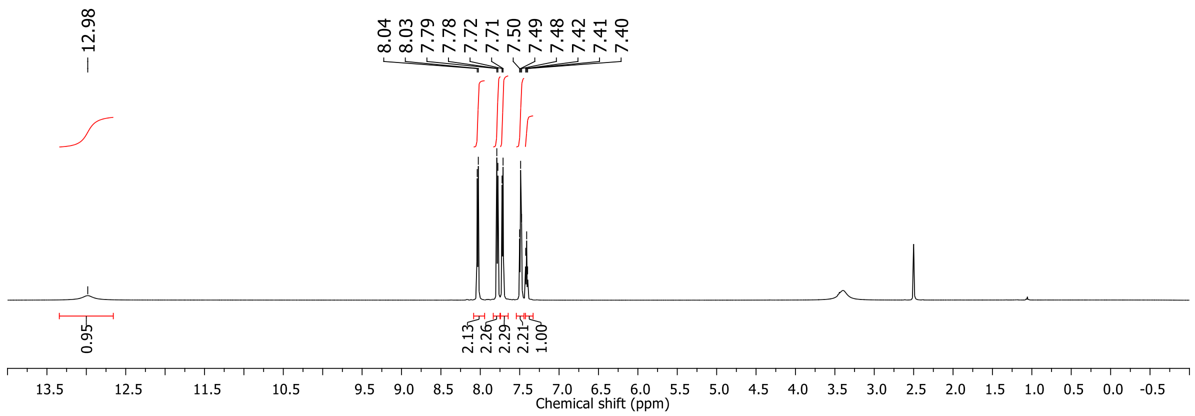

Figure 29: ^{1}H NMR of compound **6** synthesised using method 2 in glassware.

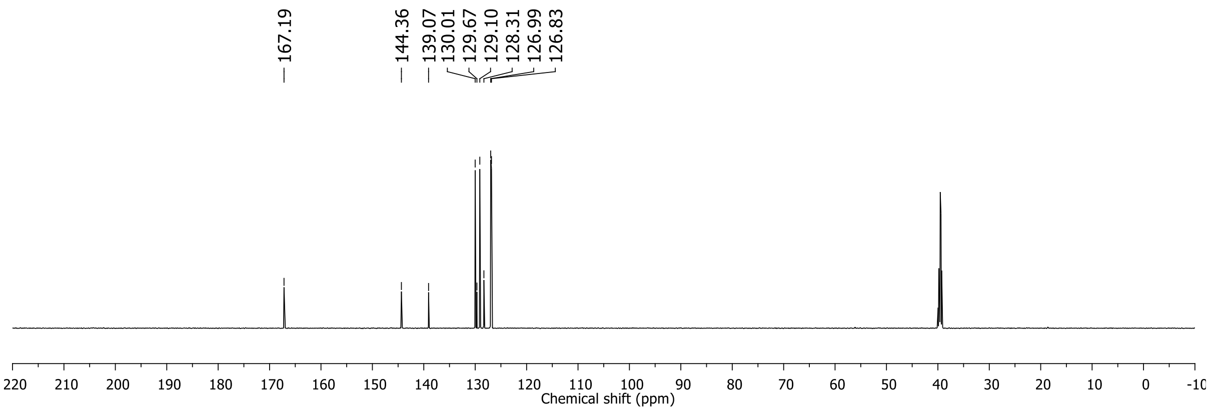

Figure 30: ^{1}H NMR of compound **6** synthesised using method 2 in glassware.

5.2.2. Reactionware synthesis

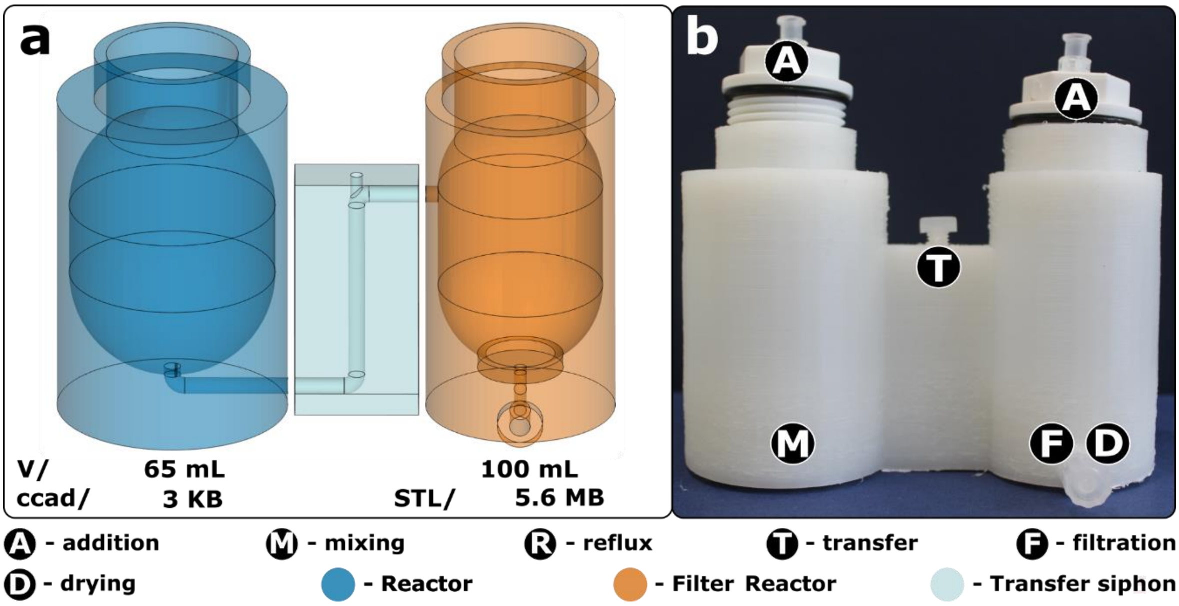

Figure 31: Reactionware for the Suzuki-Miyaura reaction, a – transparent model with individual volumes of reactors and associated file sizes, b – front view of real model, associated unit operations, their types and locations are highlighted (see legend).

4-Bromobenzoic acid (0.5 g, 2.5 mmol) and phenylboronic acid (0.37 g, 3.0 mmol) were added to Module 1 along with a magnetic stir bar. In a separate 50 mL beaker, sodium carbonate (0.80 g, 7.5 mmol) was added with 15 mL deionised water and stirred for 10 mins until fully dissolved. Once dissolved, the sodium carbonate solution was added to Module 1 via Port 1 using a syringe and stirred vigorously for 10 mins until all reactants were dissolved. Once fully dissolved, Port 1 on Module 1 was closed using a 1” polypropylene screw cap and the reaction vessel was lowered into a pre-heated water bath and the solution was heated to 70 °C. Once the desired temperature was reached, the palladium catalyst stock solution (1.0 mL, 0.25 mM) was added to Module 1 via Port 1 and the solution stirred for a further 30 mins at 70 °C. After 10 mins of heating, the product began to precipitate as a white solid and the solution was stirred for a further 20 mins to ensure reaction completion. After this time had elapsed, the reaction vessel was allowed to cool to room temperature before being placed into an ice bath situated on a magnetic stir plate. To Module 1, hydrochloric acid (25 mL, 1 M) was added slowly via Port 1, with vigourosly stirring using a disposable pipette. Once all the acid was added, the reaction mixture was stirred vigourosuly for a further 5 mins. Once this time had elapsed, the screw cap equipped with a Luer lock was replaced on Port 1 and a nitrogen gas line attached to P_{aux} on Port 1. With the line attached and the slow flow of nitrogen gas into Module 1. Whilst stirring, the screw valve (V1) was opened slightly to allow the reaction solution to be transferred from Module 1 into Module 2 (equipped with a stir bar). Once all the solution is transferred, the screw valve was tightened and the nitrogen line removed from P_{aux} on Port 1. To isolate the crude product by vaccum filtration, the vaccum line was attached to Port 3 on Module 2 and 5 mL of deionised water added directly into Module 2 via Port 2 to wash the crude product. The vaccum was applied for 5 mins to ensure complete dryness. To isolate the purified product, 10 mL of ethanol was added to Module 2 via Port 2, along with 20 mL of deionised water and stirred at room temperature for 5 mins until complete dissolution was observed. To this mixture, hydrochloric acid (5 mL, 1 M) was added slowly to Module 2 via Port 2 over 5 mins using a disposable pipette. Once all added, the reaction solution was stirred vigourosuly for 30 mins at room temperature to ensure reaction completion. To obtain the purified product, the same procedure for vaccum filtration was followed as above, washing the product with 5 mL of ice-cold ethanol prior to pulling the vaccum to remove any remaining impurities.

The product, **6**, was then dried thoroughly overnight under vaccum yielding 350 mg (1.77 mmol, 71%) of the target product with 92 % purity according to the ESI(-) LC-MS.

^{1}H NMR (600.1 MHz; 303 K; DMSO; δ, ppm; J, Hz): 12.96 (broad <u>H</u>O-R), 8.03-8.02 (2H, d), 7.80-7.79 (2H, d), 7.74-7.73 (2H, d), 7.51-7.49 (2H, t), 7.43-7.41 (1H, t).

^{13}C NMR (150 MHz; 303 K; DMSO; δ, ppm; J, Hz): 167.2, 144.4, 139.1, 130.0, 129.7, 129.1, 128.3, 127.0, 126.8.

ν_{max} (neat, cm^{-1}) 2807, 2545, 1672, 1606, 1581, 1560, 1487.

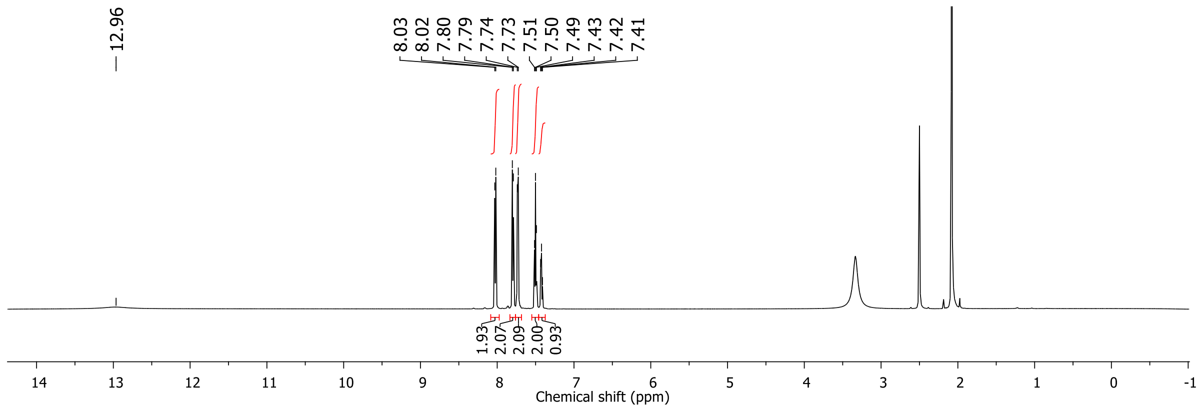

Figure 32: ^{1}H NMR of compound **6** synthesised using method 2 in reactionware.

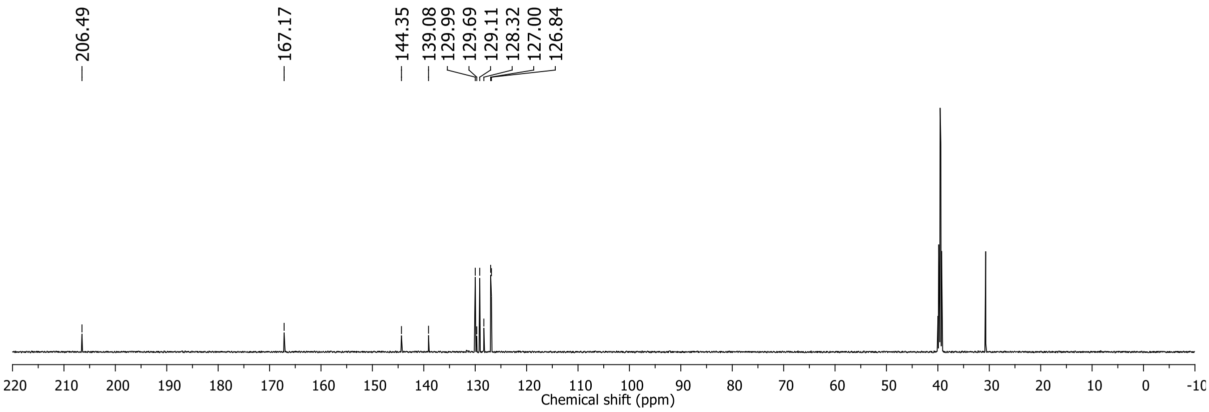

Figure 33: ^{13}H NMR of compound **6** synthesised using method 2 in reactionware.

5.2.1. Operations Table

Operation	Icon	Reagent (g, mL)	From	To	Solvent (mL)	Temp (°C)	Stir Rate (RPM)	Time (min)	Hazard
**Add**		4-bromobenzoic acid (0.5 g, 2.5 mmol)	Spatula	Module 1		RT	0	0.5	
**Add**		phenylboronic acid (0.37 g, 3.0 mmol)	Spatula	Module 1		RT	0	0.5	
**Add**			Syringe	Module 1	sodium carbonate (0.80 g in 15 mL water, 7.5 mmol)	RT	0	0.5	
**Mix**				Module 1		RT	250	10	
**Heat**				Module 1		90	250	30	
**Add**				Module 1	palladium catalyst stock solution (1.0 mL, 0.25 mM)	90	250	0.5	
**Mix**				Module 1		90	250	30	
**Cool**				Module 1		RT	250	30	
**Add**		1 M HCl (25 mL)		Module 1		RT	500	10	Corrosive
**Mix**				Module 1		RT	500	5	
**Transfer**			Module 1	Module 2		RT	500	5	
**Filter**				Module 2		RT	500	0.5	
**Add**				Module 2	5 mL H_2O	RT	250	0.5	
**Dry**				Module 2		RT	250	5	
**Add**				Module 2	EtOH (10 mL)	RT	250	0.5	
**Add**				Module 2	deionised water (20 mL)	RT	250	0.5	

**Mix**	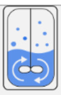			Module 2		RT	250	5	
**Add**	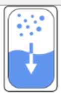	Hydrochloric acid (5 mL, 1 M)		Module 2		RT	500	5	Corrosive
**Mix**	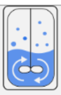			Module 2		RT	500	30	
**Cool**	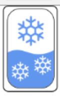			Module 2		RT	250	20	
**Filter**	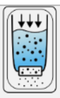			Module 2	5 °C EtOH (5 mL)	RT	250	5	
**Dry**	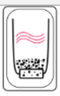			Module 2		RT	250	1440	

Table 11: Operations table for the alternative execution of the cartridge synthesis of 4-phenyl benzoic acid using method 2 (compound **6**).

6. Sulfanilamide synthesis

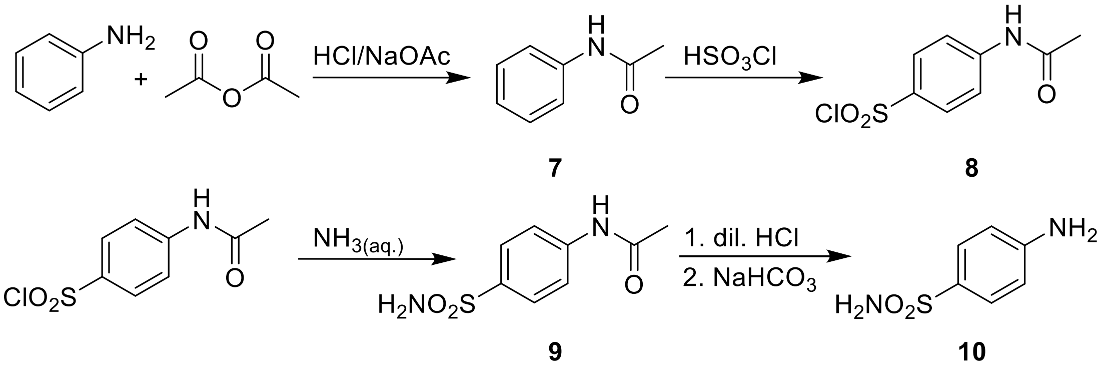

Scheme 8: four step synthesis scheme for the synthesis of sulfanilamide (compounds **7** - **10**).

6.1. Glassware synthesis

Glassware procedure was repeated as per reference.[3]

6.2. Reactionware synthesis

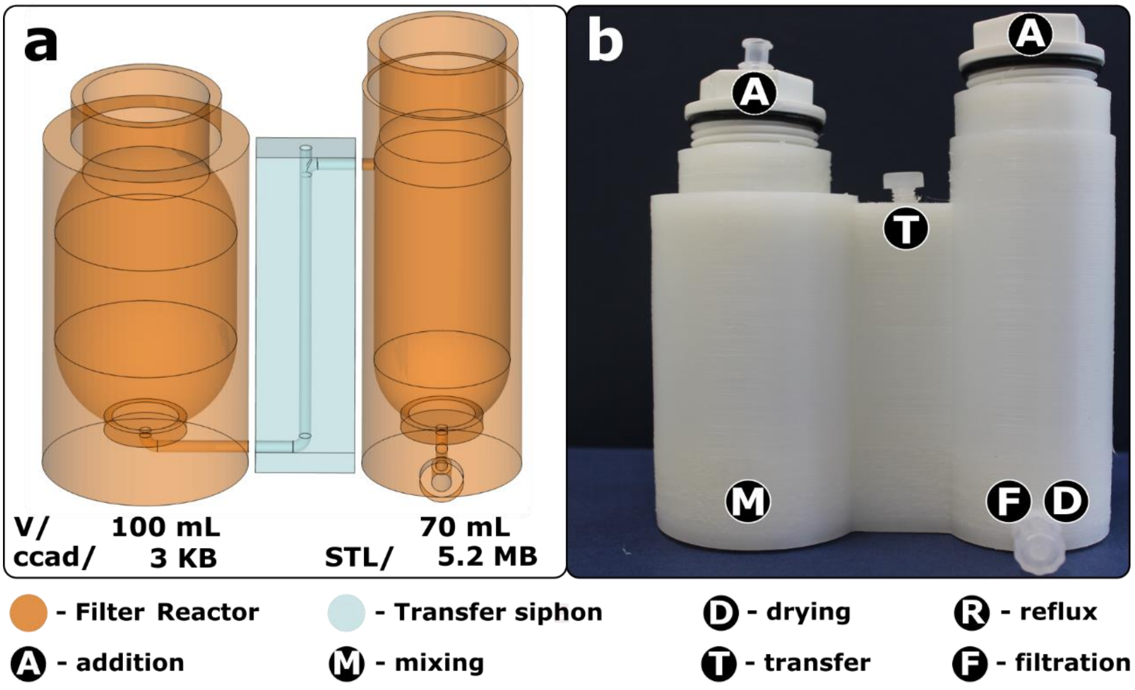

Figure 34: Reactionware for the 4-step synthesis of Sulfanilamide, where **a** – transparent model with individual volumes of reactors and associated file sizes, **b** – front view of real model, associated unit operations, their types and locations are highlighted (see legend).

Acetanilide (Compound **7**): Aniline (2 g, 21.5 mmol, 1 eq.) was dissolved in H_2O (60 mL) and conc. HCl (2 mL) in Module 1. Acetic anhydride (2.4 mL, 25.4 mmol, 1.18 eq.) and aqueous sodium acetate (2 g in 12 mL) were added simultaneously to Module 1 with vigorous stiring. The mixture was stirred for 10 minutes and reactionware was submerged into an ice bath for 30 mins. The monolith was removed

from the ice bath and allowed to return to RT. Then, a filtration was carried out in Module 1, a vacuum line attached to it, and the residue (compound A) was left to dry at RT for 24 hours.

^{1}H NMR (600.1 MHz; 303 K; $CDCl_3$; δ, ppm): 7.47-7.46 (2H, d), 7.40 (1H, s), 7.30-7.27 (2H, t) 7.09-7.07 (1H, t), 2.15 (3H, s).

^{13}C NMR (150 MHz; 303 K; $CDCl_3$; δ, ppm): 168.1, 137.4, 128.6, 124.0, 119.5, 24.1.

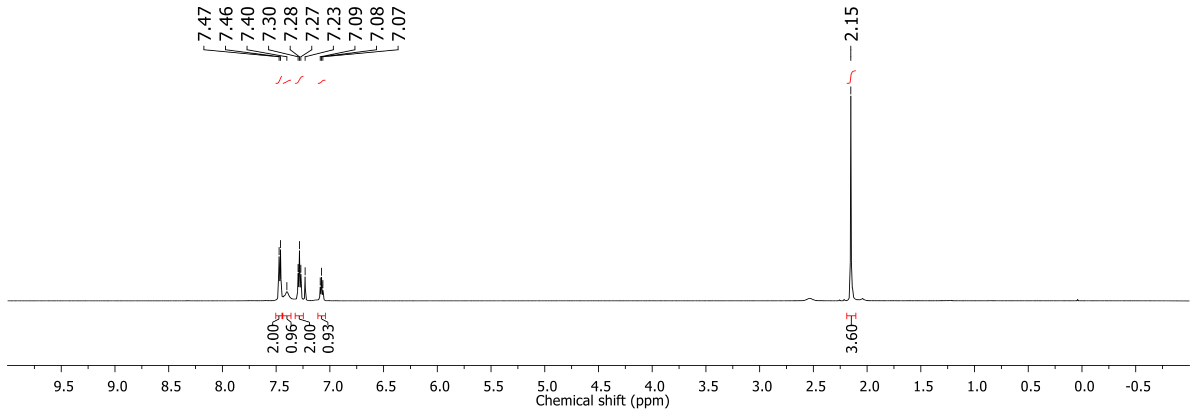

Figure 35: ^{1}H NMR of acetanilide synthesized in reactionware (compound **7**).

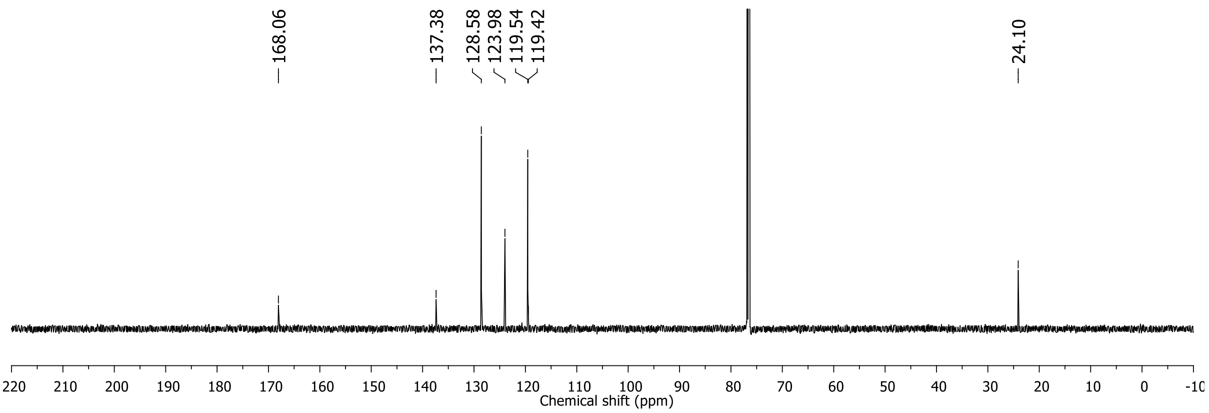

Figure 36: ^{13}C NMR of acetanilide synthesized in reactionware (compound **7**).

***p*-Acetamidobenzenesulfonyl chloride** (Compound **8**): The vacuum line was detached from Module 1. With stirring, chlorosulfonic acid (6 mL, 90.1 mmol, 4.19 eq.) was added to Module 1 **slowly** using a micropipette and left to sit for 10 minutes. Reactionware was submerged into a pre-heated oil bath (T=120 °C) and left to stir for another 10 minutes. After that period, the monolith was taken out and left to cool to RT. Cold H_2O (24 mL, ~5 C°) was loaded into Module 2 with a stir bar. With **extreme caution** and under stirring, the mixture from Module 1 was transferred to Module 2 by applying compressed air to the top of Module 1. The screw-valve was positioned such as to allow flow between the two modules, but retain the flowing mixture inside reactionware. A precipitate formed in Module 2 which was later collected by performing a filtration.

^{1}H NMR (600.1 MHz; 303 K; $CDCl_3$; δ, ppm): 7.96-7.95 (2H, d), 7.75-7.74 (2H, d), 7.56 (1H, s), 2.22 (3H, s).

^{13}C NMR (150 MHz; 303 K; $CDCl_3$; δ, ppm): 168.2, 143.7, 138.4, 128.3, 118.9, 24.4.

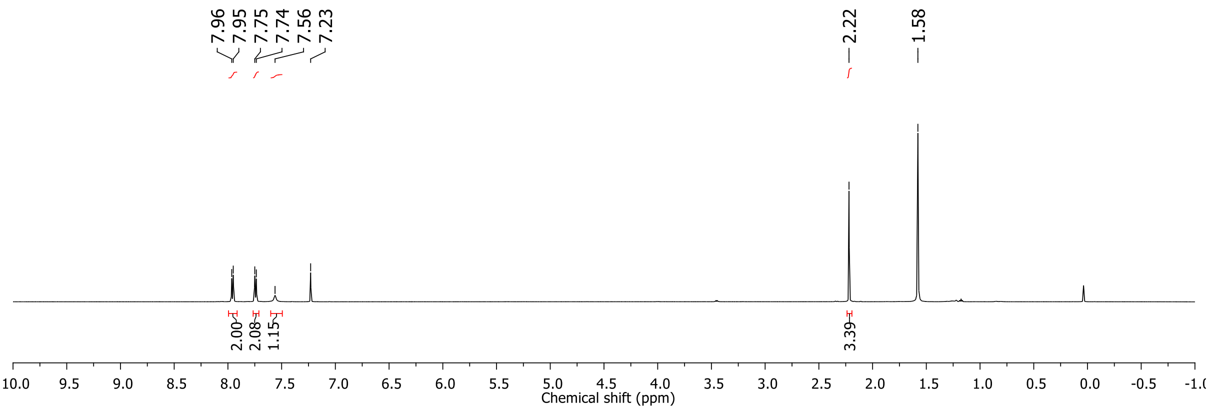

Figure 37: ^{1}H NMR of *p*-acetamidobenzenesulfonyl chloride synthesized in reactionware (compound **8**).

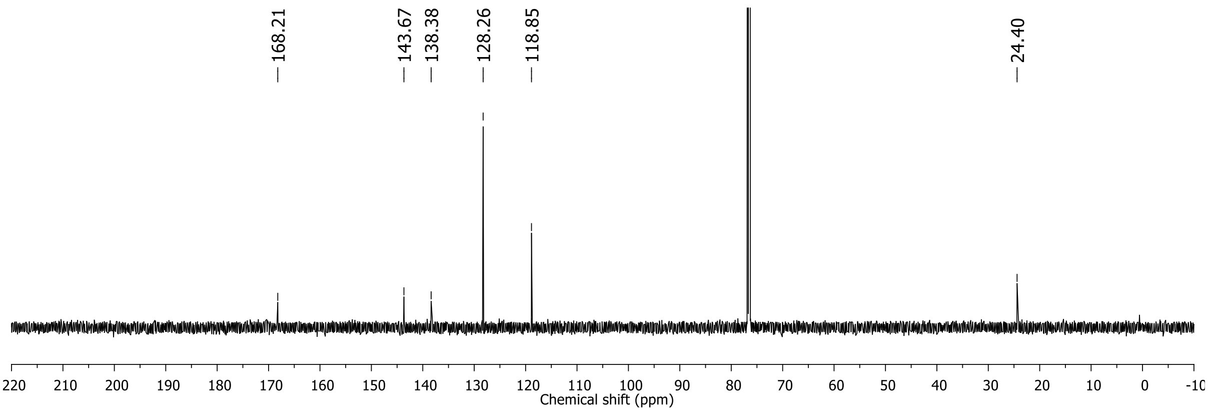

Figure 38: ^{13}C NMR of *p*-acetamidobenzenesulfonyl chloride synthesized in reactionware (compound **8**).

***p*-Acetamidobenzenesulfonamide** (Compound **9**): 25 % NH_3/H_2O (11 mL, 282 mmol, 13.1 eq.) and more H_2O (11 mL) was added to Module 2 using a syringe. Reactionware was submerged into a pre-heated oil bath (T=120 °C) and left to stir for 40 minutes. After that period, the monolith was taken out and left to cool to RT. The reactionware monolith was submerged in an ice bath for another 20 minutes before carrying out the final filtration in Module 2 affording the product compound as a white solid.

^{1}H NMR (600.1 MHz; 303 K; DMSO; δ, ppm): 10.28 (1H, s), 7.75-7.71 (4H, m), 2.08 (3H, s).

^{13}C NMR (150 MHz; 303 K; DMSO; δ, ppm): 169.0, 142.2, 138.1, 126.7, 118.5, 24.1.

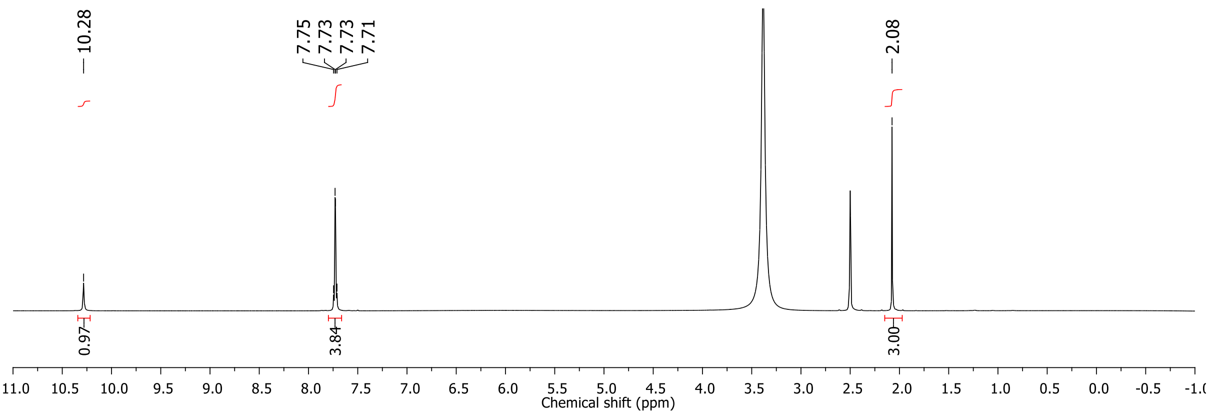

Figure 39: ^{1}H NMR of *p*-Acetamidobenzenesulfonamide synthesized in reactionware (compound **9**).

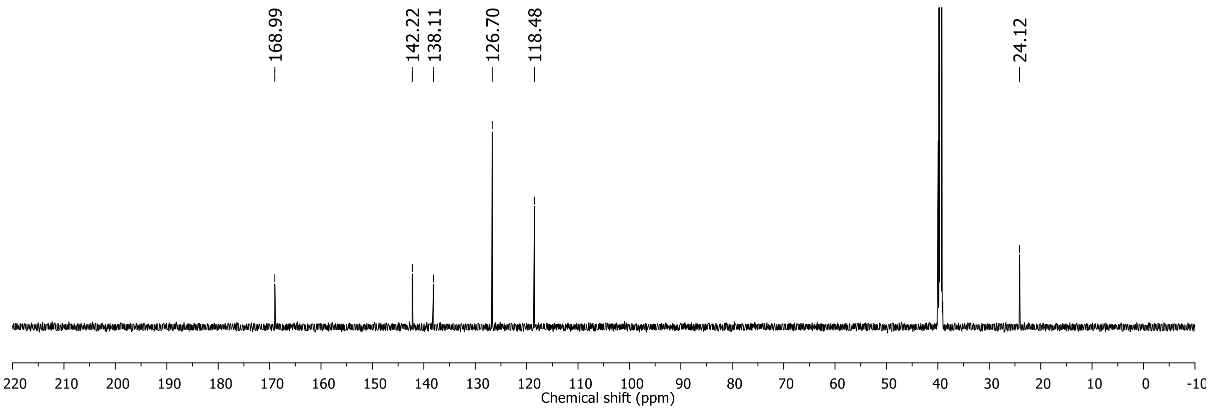

Figure 40: ^{13}C NMR of *p*-Acetamidobenzenesulfonamide synthesized in reactionware (compound **9**).

Sulfanilamide (Compound **10**): Conc. HCl (3.6 mL) and H_2O (7.2 mL) was added to Module 2 and left to stir for 10 minutes. The reactionware monolith was placed in a conventional microwave oven and irradiated for 2 hours at 20 % power. After irradiation, the solution was allowed to cool to RT. Aqueous Na_2CO_3 (0.94 M, 2 g in 12 mL H_2O) was added in portions under stirring to reach a pH of 7. Final product compound precipitated out and was collected *via* filtration as a white powder (379 mg, **22%**).

^{1}H NMR (600.1 MHz; 303 K; DMSO; δ, ppm): 7.45-7.43 (2H, d), 6.87 (2<u>H</u>N-R, s), 6.59-6.58 (2H, d), 5.78 (2<u>H</u>N-R, s).

^{13}C NMR (150 MHz; 303 K; DMSO; δ, ppm): 151.9, 130.0, 127.4, 112.5.

v_{max} (neat, cm^{-1}) 3549, 3461, 3387, 3325, 3190, 1629, 1594, 1494, 1433.

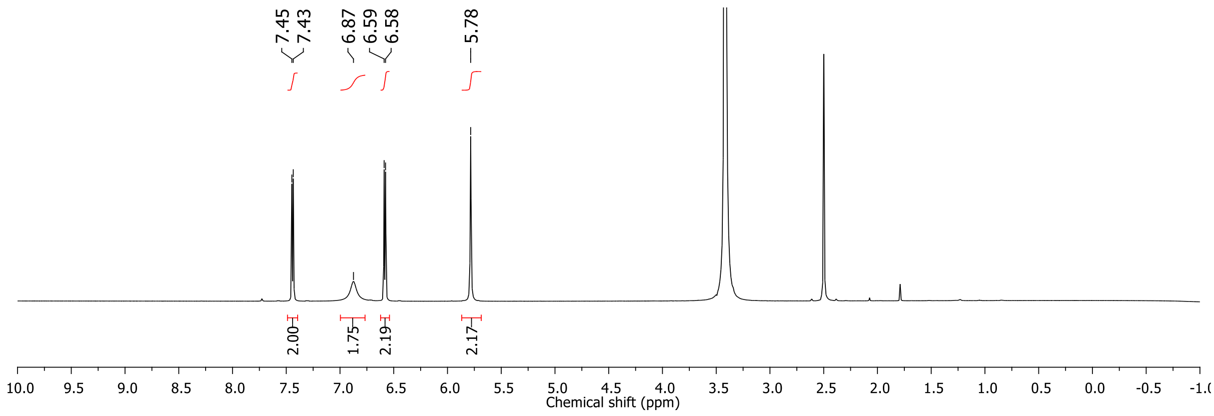

Figure 41: ^{1}H NMR of sulfanilamide synthesized in reactionware (compound **10**).

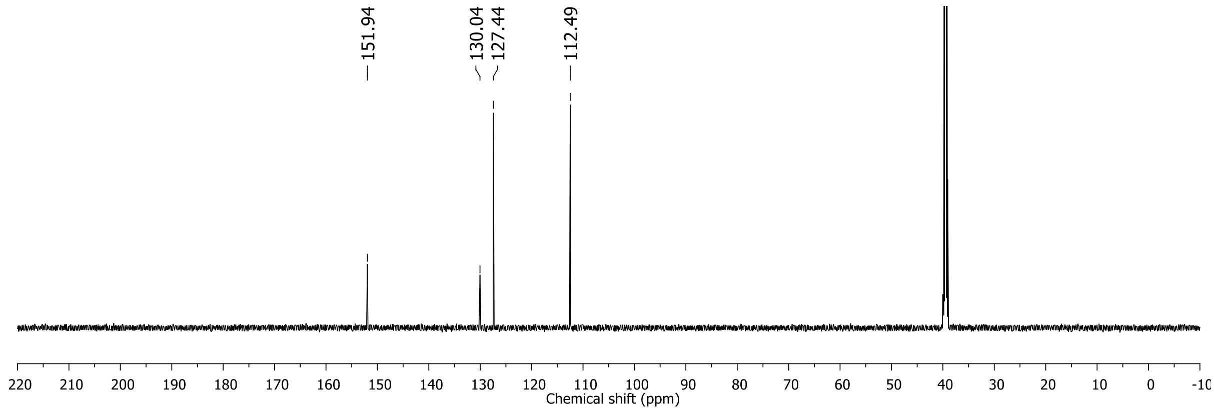

Figure 42: ^{13}C NMR of sulfanilamide synthesized in reactionware (compound **10**).

6.2.1. Operations Table

Operation	Icon	Reagent (g, mL)	From	To	Solvent (mL)	Temp (°C)	Stir Rate (RPM)	Time (min)	Hazard
**Add**	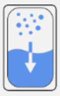	Solution of aniline (2 g, 21.5 mmol, 1 eq.) in water (60 mL) and conc. HCl (2 mL)	Syringe	Module 1		RT	750	0.5	
**Add**	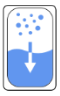		Syringe	Module 1	Acetic anhydride (2.4 mL, 25.4 mmol, 1.18 eq.),	RT	750	0	
**Add**	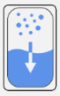		Syringe	Module 1	aqueous sodium acetate (2 g in 12 mL water)	RT	750	0	
**Mix**	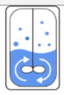			Module 1		RT	750	10	

**Cool**				Module 1		0	500	30	
**Dry**				Module 1		RT	500	overnight	
**Heat**		No reagent added, pre-heat oil bath		Module 1		120	250	10	
**Add**		chlorosulfonic acid (6 mL, 90.1 mmol, 4.19 eq.)		Module 1		RT	500	10	Highly corrosive
**Mix**				Module 1		120	500	10	
**Add**		Cold H_2O (24 mL, ~5 °C) + stir-bar	Syringe	Module 1		RT	0	25	
**Cool**				Module 1		RT	0	0.5	
**Transfer**			Module 1	Module 2		RT	500	5	
**Mix**				Module 2		RT	500	25	
**Filter**		No reagent added, filtering precipitate		Module 2		RT	500	10	
**Heat**		No reagent added, pre-heat oil bath		Module 2		120	250	10	
**Add**		25 % NH_3/H_2O (11 mL, 282 mmol, 13.1 eq.)	Syringe	Module 2		120	500	40	
**Cool**		Cooled to RT and placed in ice-bath		Module 2		0	0	60	
**Filter**		Filtering precipitate		Module 2		RT	250	10	
**Add**		Conc. HCl (3.6 mL) and H_2O (7.2 mL)		Module 2		RT	250	0.5	Highly corrosive
**Mix**				Module 2		RT	250	10	
**Heat**		Heated in microwave at 20% power		Module 2		100	0	120	
**Cool**		Cool to RT		Module 2		0	250	30	
**Mix**				Module 2		RT	250	10	
**Add**				Module 2	Aqueous Na2CO3 (0.94 M, 2 g in 12 mL	RT	250	10	

					H_2O) added in 1 mL portions to reach pH 7				
**Filter**	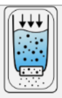	Filtering precipitate		Module 2		RT	250	5	
**Dry**	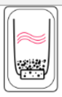	Drying precipitate		Module 2		RT	0	1440	

Table 12: Operations table for the execution of the cartridge synthesis of sulfanilamide (compound **10**).

Section B: Laboratory Tutorials for Students

Abstract

The advent of 3D-printed reactors brings with it the opportunity to translate traditional undergratuade chemistry experiments into workflow. 5 examples of experiments are described below which are designed to promote learning for students and provoke thought for demonstrators. We have provided detailed information in the following sections for each experiment, with procedures for reproducing the syntheses in glassware and reactionware (for demonstrators) and a series of pre- and post-lab questions for students to guide their learning while using the reactionware in the laboratory.

1. **Ester hydrolysis – functional group interconversion and unknown product compound characterisation**
2. **Wittig reaction – formation of double bonds and chemistry of colour**
3. **MIDA boronates – synthesis of useful small organic precursors**
4. **Suzuki-Miyara coupling – carbon-carbon sigma bond formation**
    a. **Method 1**
    b. **Method 2**
5. **Sulfanilamide - process selection, subsequent reactor design and batch synthesis of drug molecules**

Each of the laboratory experiments will be comprised of the following:

- Introduction
- Pre-lab questions
- Experimental procedure
- Post-lab questions

7. MIDA boronate formation: preparing coupling reagents

Introduction

Being one of the most famous carbon-carbon bond forming reactions, the Nobel prize award winning Suzuki-Myiaura reaction has been used extensively in natural product, pharmaceutical and other molecule synthesis for decades. The reaction involves a vinyl or aryl boronic acids reacting with vinyl or aryl halides and is catalysed by palladium (0) complexes.[4] This makes the diversity of boronic acid substrates very desirable by chemists. Often, a particular boronic acid can be unstable and will degrade over time under bench conditions. This can be avoided by stabilising the boronic acid by turning it into a bench stable ester, which in turn can be used for the Suzuki coupling. One popular method uses MIDA boronate esters as the bench stable versions of boronic acids.[5]

In this experiment you will be tasked to stabilize a boronic acid by turning it into an MIDA ester version of it. Characterisation can be performed using $^{1}H/^{13}C/^{15}B$ NMR and IR.

Experimental procedure:

1. Add boronic acid (1 mmol, 1 eq.) and MIDA anhydride (3 mmol, 3 eq.) to a flame-dried glass beaker and dissolve under stirring in anhydrous 1,4-dioxane (2.5 mL).
2. Flush **M1** with an Ar balloon.
3. Transfer mixture to **M1** using a syringe, making sure all other ports are fully sealed.
4. Equip **M1** with a 3D-printed high surface polypropylene condenser via Port 1, making sure to close all other ports.
5. Lower the vessel into a pre-heated oil bath at 100 °C and stir for 24 hours.
6. After this time has elapsed, remove the vessel from the oil bath and allow to cool completely to room temperature (approx. 30 minutes).
7. Once cooled, add H_2O (18 µL, 1 mmol, 1 eq.) and 1,4 dioxane (2.5 mL) to **M1** via Port 2 and leave to stir for 1h at RT.
8. After this time has elapsed, lower the vessel in a pre-heated oil bath at 50 °C and attach a vaccumm line to **M1** via Port 1.
9. Once attached, evaporate the solvent for 24 hrs at 50 mmHg pressure.
10. One dry, remove from the oil bath and allow to cool to RT (approx. 30 mins), detach the vacuum line from Port 1.
11. Add acetone (5 mL) to **M1** via Port 1 and stir at RT for 20 minutes.
12. Add a mixture of Et_2O:hexane (1:1, 40 mL) to **M2** under stirring,
13. Attach a nitrogen line to Port 1 on **M1** and transfer the reaction mixture to **M2** under constant stirring.
14. Once fully transferred, leave **M2** to stir for 30 minutes at RT.
15. During this time, a white solid will precipitate which can be filtered.
16. Attach a vaccum line to the bottom port on **M2** and perform a filtration to isolate the solid precipitate of the product.
17. Keep the vacuum line attached to **M2** and leave to dry overnight under vacuum to yield a white powder of the desired MIDA boronate ester.
18. Characterise the product using NMR and IR.

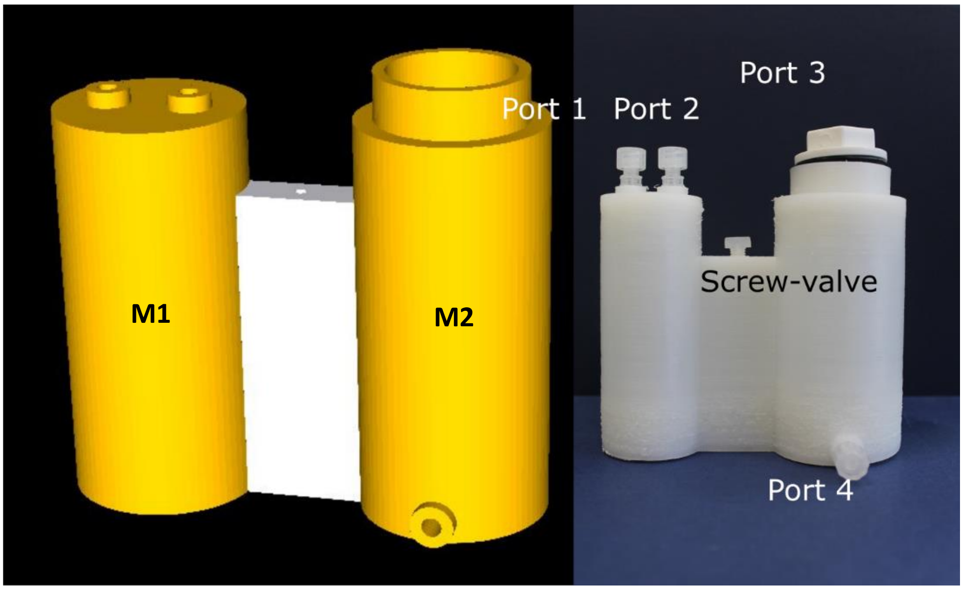

Figure 43: Dual cartridge model of the reactionware for MIDA boronate ester synthesis (left); labelled version of the 3D printed cartridge (right).

Pre-lab and post-lab questions:

1. What does it mean if a reagent is labelled as 'hygroscopic'?
2. What measures can be taken to protect moisture-sensitive reactions from degradation?
3. Why is an excess amount of MIDA anhydride needed in this reaction?
4. Considering the potential by-product(s) in this reaction, why is water added in step 6 prior to work-up?
5. What role does acetone play in this reaction?
6. Considering the stoichiometric amounts of each reagent, construct a rate-law for this reaction and determine the order of rate i.e. zero, first, second etc.
7. Considering solvent density, which layer is on top upon solvent addition in step 9 (aqueous or organic) and which layer will contain the desired product?
8. How might one monitor this reaction to determine if it's complete? Hint: consider characteristic features for both the starting materials and product
9. How could one improve this synthesis to make it greener according to the 12 principles of green chemistry?
10. What effect would temperature have on this reaction?
11. Draw a curly-arrow mechanism for this reaction

<u>Guideline Answers:</u>

1. The term hygroscopic describes a reagent which absorbs water.

2. To prevent this degradation from occurring either the reaction can be formed in a fully enclosed system which has been prior flushed with an argon balloon or the reaction can be performed under inert atmosphere of nitrogen inside a glove-box.

3. The excess of MIDA anhydride is used to react with any remaining starting material to ensure it is all converted into the more reactive MIDA acid. This excess will also ensure the reaction equilibrium shifts more owards the formation of product as understood by Le Chatiliers' Principle of dynamic equilibria. Acts as an *in situ* dessicant.

4. The water is added to convert the MIDA anhydride to the highly polar diacid MIDA.

5. Acetone dissolves the product not the highly polar MIDA diacid.

6. The rate law for this reaction is as follows: this paper describes the rate law for the reverse reaction, hydrolysis of the MIDA boronate esters as proceeding by two different (fast or slow) mechanisms. Note – the reaction of MIDA anhydride with a boronic acid is comparable to the reverse of a slow release hydrolysis pathway. - https://www.ncbi.nlm.nih.gov/pmc/articles/PMC5115273/

7. Sufficient water isn't added here to be doing a phase separation.

8. This reaction can be monitored by NMR using timed experiments since the formation of the characteristic MIDA ester peaks around 4 ppm (two roofing doublets) will indicate product formation. Also using boron NMR could measure the rate of product formation over time.

9. By using greener solvents (such as water) and also lower temperatures for the synthesis would make the procedure much greener according to the 12 principles.

10. Increasing the temperature would increase the rate of reaction and subsequently push the equilibrium towards the product formation side of the equilibrium as shown by the rate law.

11. Mechanism below is proposed and uses three equivalents of MIDA anhydride: A few notes are as follows: i) the reaction would not proceed in water and ii) addition of water would quench the produced salts at the end of the reaction, and 3 eq. was used to ensure reproducibility with commercial boronic acids of variable moisture content.

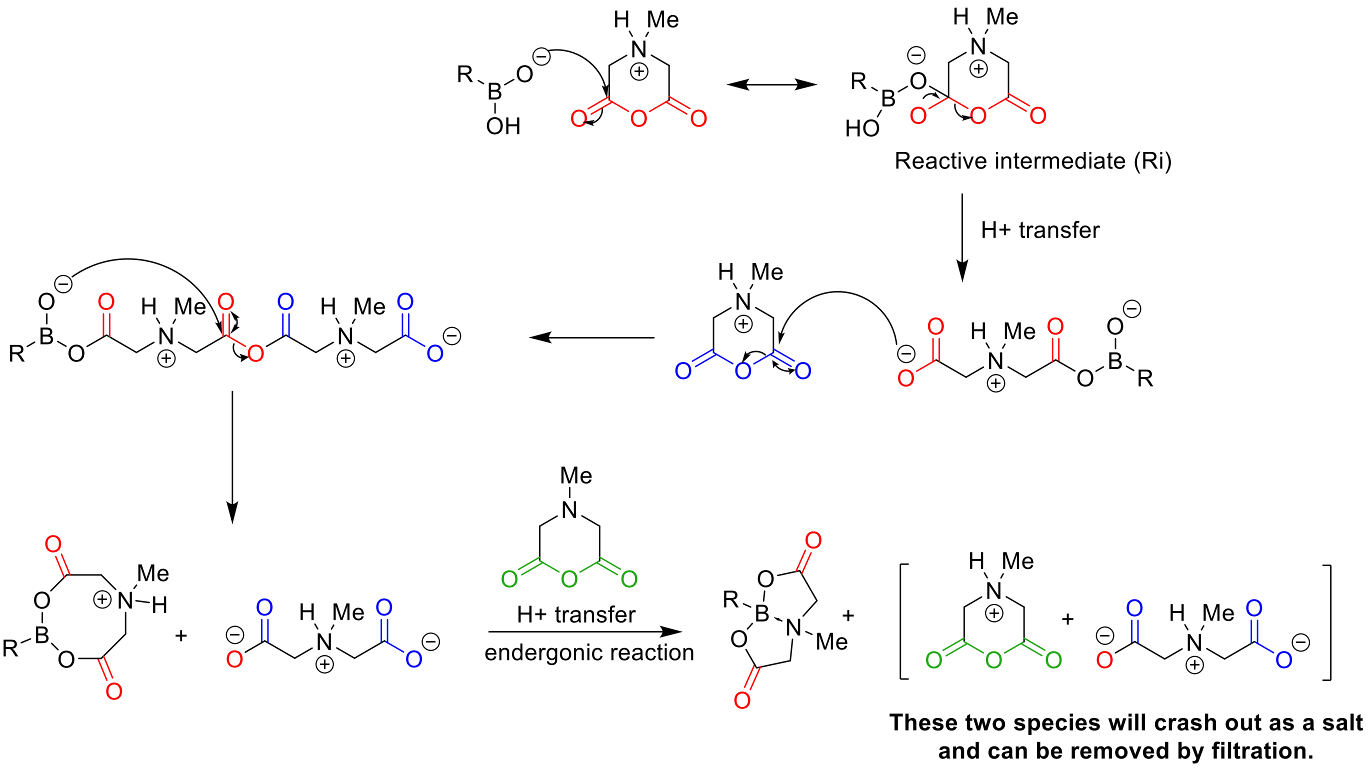

8. Ester hydrolysis: FGIs and identification of unknown compound

Introduction

Hydrolysis is the cleavage of a chemical species by water.[6] Medicinal chemists often use hydrolysis to afford desired products. The hydrolysis of esters has been employed in the synthesis of desired drug products for decades, and is still important today.[7] Often ester hydrolysis is vital step in a multi-step organic synthesis of other functional groups such as amides.

In this experiment, you will be tasked to identify an unknown ester by carrying out a functional group interconversion (FGI) reaction (hydrolysis) and characterising the final product using $^{1}H/^{13}C$ NMR and IR.

Pre-lab questions

1. Each step in the ester hydrolysis reaction is in equilibrium. Comment on all the possible ways to drive the equilibrium?
2. If some of the *ester* remains at the end of the reaction, what are some ways to get rid of it?
3. If excess *alcohol* remains at the end of the experiment, what are some ways to get rid of it?
4. What effect does temperature have on the rate of this reaction, considering the rate law for this reaction?

5. Comment briefly on the stability of carboxylate anions via resonance.
6. Draw a complete mechanism for the acid-catalyzed esterification of acetic acid with ethanol (the reverse reaction of the one to be carried out).

Guideline Answers:

1. The equilibrium can be shifted towards the desired product by either: (a) using a large excess of one reagent or (b) by removing one of the products. This is according to Le Châtelier's Principle.
2. Esters can be cleaved back into a carboxylic acid and an alcohol by reaction with water and a catalytic amount of acid (i.e. the product mixture can be resubjected to the reaction conditions).
3. Initial washing with water will remove most of the water soluble species which includes any excess alcohol (ethanol), carboxylic acid (acetic acid), and sulfuric acid. The ester is not very soluble in water so will separate into a separate layer.
4. Given the reaction is first order with respect to concentration of the starting materials, an increase in temperature (as shown by the integrated rate law), will result in a directly correlated increase in the rate of reaction.
5. Carboxylate ion is stabilised by resonance. Carboxylate ion is resonance hybrid of two equivalent structures so that the negative charge is delocalised on both oxygen atoms. This leads to stability through resonance. Carboxylic acids are more acidic than phenols. Carboxylate ion has two equivalent resonance structures in which negative charge is delocalised over more electronegative two oxygen atoms. Phenoxide ion has non equivalent resonance structures in which negative charge is delocalised over one oxygen atom and less electronegative carbon atom. Hence, carboxylate ion is more stable than phenoxide ion.
6. The mechanism for this transformation is shown below, where R' = Et:

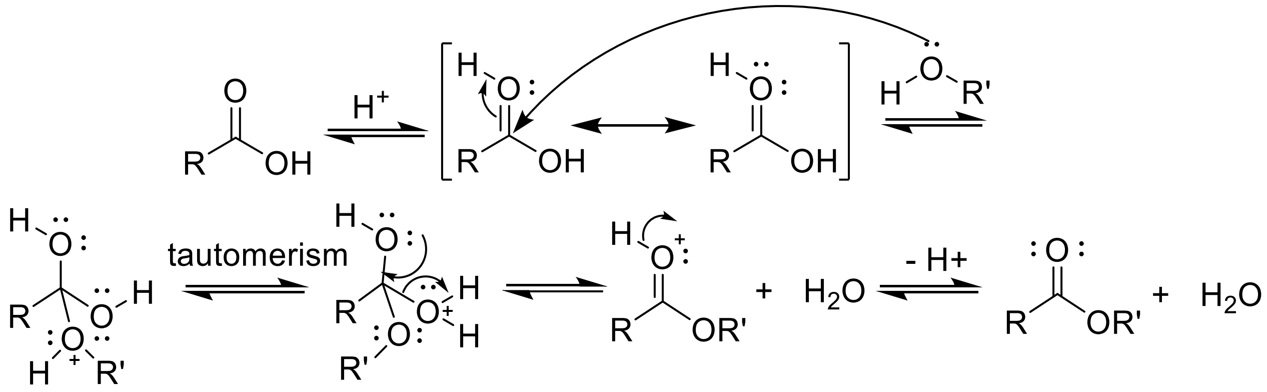

Experimental procedure

1) Add 20-25 drops of unknown ester into **M1** through Port 1, equipped with a stirrer bar and 2 boiling stones.
2) Add 15 mL aqueous sodium hydroxide (5 M) into **M1** through Port 1 followed by 3 mL ethanol.
3) Attach reflux condenser to **M1 via Port 2.**
4) Lower cartridges into oil bath and heat until reflux for about 25 min.
5) To initiate the transfer of liquid from **M1** to **M2**, open the screw valve slightly using the screwdriver with a light flow of nitrogen gas through Port 1 into **M1**.
6) Add 15 mL aqueous hydrochloric acid (5 M) to **M2** via Port 3 (carboxylic acid crashes out). Use universal paper to check pH. Add more HCl (5 M) via Port 3 until pH is approx. 1.
7) Apply pressure to **Port 1** / vacuum to **Port 4** and dry solid in **M2.**
8) Measure melting point of solid carboxylic acid, identify unknown starting ester.

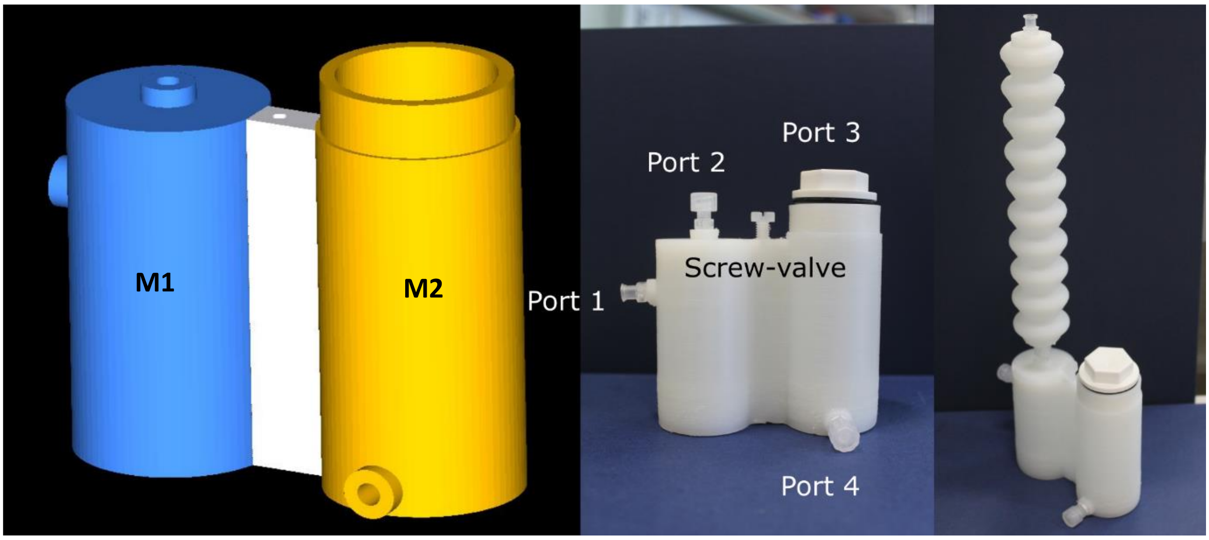

Figure 1: Dual cartridge monolith for ester hydrolysis (left) and labelled 3D printed model (right).

Ester	*Carboxylic acid*	*Melting point (°C)*
Methyl benzoate	Benzoic	122-123

Table 13: Ester, carboxylic acid and it's respective melting point, other esters can be used as unknowns.

Post-lab questions

1) Provide a curly arrow reaction mechanism for the reaction, making clear the role of base in the reaction.

2) What is the purpose of adding aqueous sodium hydroxide in step 2?

3) What are the best ways to determine if any residual acid is mixed in with your ester? Consider mp, NMR, IR, & UV and comment on the usefulness of each.

4) What gas was evolved during step 6? Write a balanced equation for the reaction that produced the gas.

5) Using the Twelve Principles of Green Chemistry, what principles does this experiment illustrate? Be sure to look at side products, stoichiometry (to see if there are excess reagents), and the safety of by-products.

6) How might you improve the "greenness" of this synthesis?

Guideline Answers:

1. The reaction mechanism for the base-catalysed ester hydrolysis is shown below. As shown, the role of base in this reaction is as a catalyst to promote formation of the desired product. (Chemistry Steps)

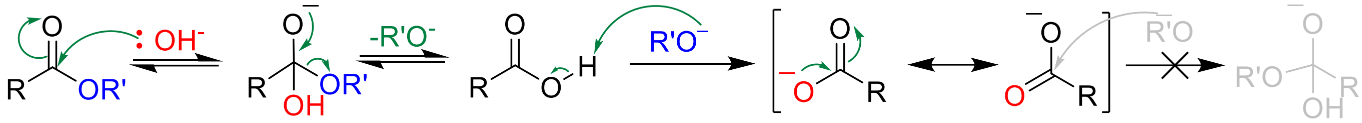

2. The addition of NaOH in Step 2 facilitates the elimination of the ester group since the excess of base added will shift the position of equilibrium towards the desired hydroxy-ester product allowing for the deprotonation of the carboxylic acid in step 3.

3. Given the different functional groups present, the best analytical technique would be IR since we can observe the disappearance of the broad -OH peak in the spectrum once the ester functional group is cleaved. If there was any acid remaining the -OH would still be seen in addition to the peaks between 1680-1750 cm^{-1} for the -C=O bonds in both the acid and ester. UV is less useful here since the functional groups will not absorb very strongly in the obeserved region due to weak orbital-orbital transitions and so it may be difficult to effectively distinguish the different functional groups. Using NMR is effective here as the -OH group can show as broad peak but this may be unobservable due to the H-D exchange on the timescale of the experiment, at room temperature at least. To counter this, the ester will show characteristic peaks in the NMR spectrum indicating its presence as the desired product. Due to this small difference in molecular composition, NMR may not be the best choice since the majority of the NMR spectrum for the carboxylic acid and ester will have chemical shifts within the same range.

4. Step 6 is the acid-catalysed conversion of the ester into the corresponding carboxylic acid product. The reaction equation, in equilibrium, is as follows:

RCOOR' + H_2O ←→ RCOOH + R'OH (water provided by the acid in addition to the protons)

5. This synthesis highlights the principle of using catalysts to both increase the rate and sustainability (low cost) of this process. There is an equal stoichiometry of the ester and water which allows for all of the excess ester to be reacted in the conversation to the corresponding carboxylic acid. The by-products are relatively inert as the alcohol is able to be recycled and either used in other processes or used as solvents for other transformations.

6. To improve the greenness of this reaction, one could look to reduce the reaction temperature and importantly aim to use a weaker base which is still capable of the catalysis but can effectively reduce the cost and associated safety implications of this reaction in its current form.

9. Wittig olefination: forming C=C bonds

Introduction

The Wittig reaction is widely used in organic chemistry for the preparation of alkenes. It involves the reaction of aldehyde or ketone with triphenylphosphine to give an alkene and triphenylphosphine oxide. The apparent simplicity of this reaction allows for a wide variety of alkenes to be synthesised including stereospecific and chiral alkenes which have applications in the early stage production of certain pharmaceuticals.[8,9]

Depending on the solvent used, the yield of the afforded alkene can differ, which is due to the stability of the tetrahedral intermediate in the reaction in terms of solvation enthalpy. [Fleming, R. H.; Quina, F. H.; Hammond, G. S., *J. Am. Chem. Soc.* 96, 7738-7741 (1974)].

Experimental procedure:

1. Add 345 mg of 9-anthraldehyde (1.7 mmol, 1.0 eq), 0.6 g of benzyltriphenyl phosphonium chloride (1.8 mmol, 1.1 eq) and DMF (1.5 mL) to **FR1**, making sure to equip it with a magnetic stir bar and then replace the screw cap.
2. With stirring, using a 1 mL syringe, measure 0.75 mL of 50 % NaOH and add this to **FR1** dropwise via Port 1.
3. Seal Port 1 and place on a stir plate and stir for 1 hour at room temperature.
4. After this time has elapsed, add 2-propanol/H_2O (12 mL, 1:1) to **FR1** and stir for a further 10 minutes.
5. Apply vacuum (tighten drill hole screw) to **FR1** via Port 2 to filter the solution.
6. Remove vaccum line and close Port 2 on **FR1**.
7. Pre-heat an oil bath to 115 °C and add 2-propanol (12 mL) to **FR1**.
8. Submerge the vessel into the oil bath, making sure to close port 1 before attaching a reflux condensor to the auxillary Port 1.
9. With stirring, heat the mixture at 95 C° until the contents has dissolved (approx. 10 minutes).
10. Remove vessel from the oil bath and allow to cool completely to room temperature. As cooling, the Wittig product will appear to crystalise as yellow needles.
11. Attach the vaccum line as before and filter the liquid before allowing the crystals to dry under vacuum overnight.
12. Remove crystals of the product, record the weight and analyse using NMR and IR.

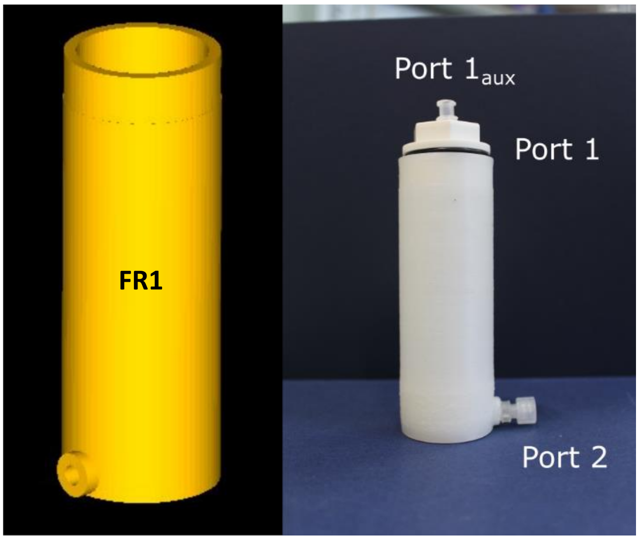

Figure 44: Single cartridge for Wittig olefination (left) and labelled 3D printed model (right).

Pre-lab questions & post-lab questions:

1. Explain the role of the phosphonium chloride in this reaction.
2. What is an expected by-product from this reaction?
3. Why is it necessary to cool the solution completely in step 9 prior to placing in an ice bath?
4. Why is only a minimal amount of ethanol required in step 8?
5. Why do you think the original aldehyde dissolved in the NaOH, but the triphenylphosphonium salt 1 did not?
6. Three intermolecular forces impact solubility: hydrogen-bonding, dipole-dipole forces, and London forces. Which of those three best explains why triphenylphosphine oxide (side product) was completely soluble in ethanol, whereas was the alkene (product) was not (at least at low temperature)?
7. When "Wittig Reagents" are formed from phosphonium salts, normally very strong bases such as butyl lithium or LDA are used. (In class, we're defaulting to butyl lithium). In today's experiment, however, we were able to use hydroxide. What structure feature in our phosphonium salt 1 made it more acidic than an ordinary phosphonium salt, enough so that hydroxide was sufficiently strong as a base to convert 1 to Wittig Reagent 3?
8. Draw a mechanism for formation of the Wittig reagent.
9. Draw a mechanism for the conversion of the aldehyde to the alkene using the Wittig reagent.
10. At the end of the reaction time, there were two neutral products formed, the desired product (alkene) and the side product triphenylphosphine oxide. What's the term for the general process that was used to selectively remove the oxide while leaving the alkene in a form that could be harvested?
11. Given an alkene is formed, two isomers (E and Z) can theoretically form. Which of these will dominate for this given aldehyde and how could one determine this from analytical data such as proton NMR spectra of the final product? (J coupling above 10 Hz = trans isomer, E)
12. How might you use TLC to monitor this reaction to determine when it has reached completion?

Guideline Answers:

1. In this reaction, benzyltriphenyl phosphonium chloride is used to form the reactive Wittig reagent which allows the formation of the alkene product to proceed in high atom economy from the corresponding aldehyde/ketone.

2. The expected by-product is triphenylphosphine oxide.

3. The slow cooling step is necessary to allow the formation of needle-like crystals of the Wittig product, where rapid cooling can lead to disordered and much smaller crystals. Placing the cooled vessel into the ice-bath allows for the crystalisation process to complete at a regulated low temperature, which further allows for this slower crystalisation growth to occur.

4. The minimal amount of ethanol is needed to dissolve any soluble impurities and allow the recrystalisation to take place without any significant loss in yield of the desired Wittig product.

5. There is the old adage when it comes to dissolution that “like dissolves like”. Most reactions are conducted in a homogeneous solution, where everything is dissolved and can move around such that reactants can collide. This is difficult to accomplish, however, when you have both strongly hydrophobic reactants (the aldehyde in this experiment) and strongly hydrophilic reactants (sodium hydroxide). The phosphonium salt is also ionic, and thus also has problems dissolving in organic solvent.

6. The differences in solubility can be explained by the extent of hydrogen bonding interactions in solution. The ionic hydroxide and the phosphonium salt can go into the water, and the aldehyde and the product alkene can go into the 2-propanol. When the ylide forms, it has no overall charge, and thus can switch phase from the water to the organic phase. (This is called a “Phase Transfer” reaction.) Note: Phase transfer can only take place at the interface between the two phases. In order to maximise contact between the two phases, it is very important that the mixture be well stirred to provide lots of small droplets and lots of surface area for organic/water contact.

7. This is because the carbanion of compound 1 that is produced is stabilised not only by the positive phosphorus, but also by conjugation with the benzene ring and is therefore very strongly nucleophilic. The positive phosporus depresses the pKa of the alpha proton such that is can be removed by hydroxide. Notice that carbanion has a resonance structure in which it is unnecessary to draw any formal charges. Either resonance structure is reasonable; 1’ has the advantage that it involves no formal charge, and has a double bond to carbon in exactly the same place where the final alkene C=C double bond ends.

8. The mechanism is shown below for the formation of the Wittig reagent and subsequent formation of the alkene product:

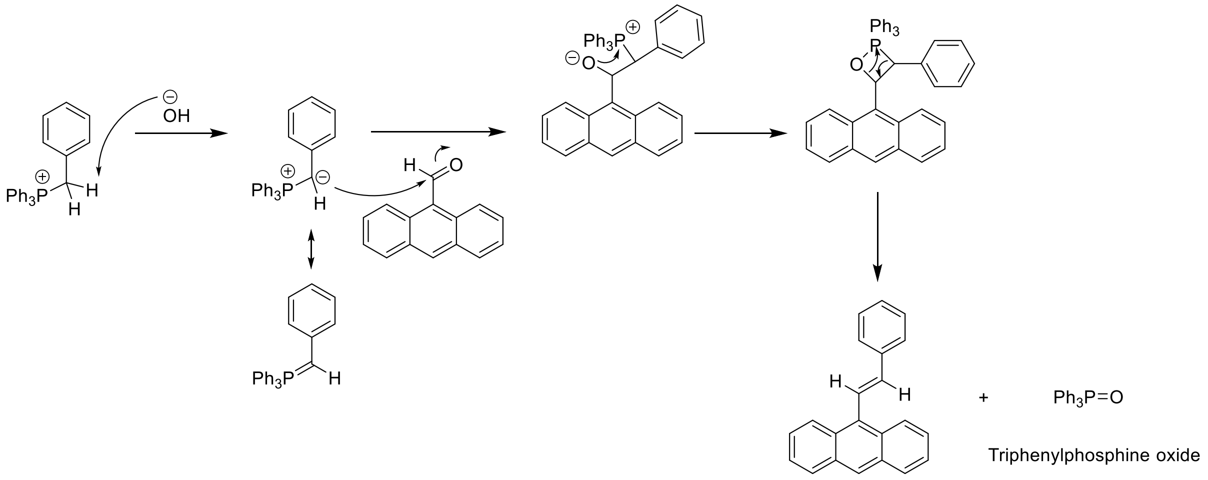

9. See Steps 5 and 6 above for formation of the alkene product from the aldehyde using the Wittig reagent.

10. The general term for selective removal of the triphenylphosphine oxide is aqueous extraction through phase transfer and works by dissolving the oxide in the water phase and forming the alkene product at the phase boundary (interface) of the solvent mixture.

11. In this reaction, the isomer selectivity is completely towards formation of the E isomer, given the bulkiness of the R groups which result in steric hindrance meaning the Z isomer cannot form. The experiment can be extended to illustrate the use of spectroscopy to elucidate stereochemistry. The infrared spectrum ($CHCl_3$ solvent) of the product has a strong peak at 962 cm^{-1}, indicating a trans —CH=CH— grouping. Unfortunately, there is also a peak at 680 cm^{-1} (because of monosubstituted benzene) that could be interpreted as cis —CH=CH—. The proton NMR spectrum ($CDCl_3$ solvent) gives a clearcut answer, however. The vinylic protons produce the expected pair of doublets of an AX system; one of these (6.82, J = 17 Hz) is clearly separated from the signal (7.3-8.5) representing the aromatic protons. (The other doublet, at 7.82, is imbedded in the aromatic signal.) The J value (as explained by the Karplus equation) proves that the structure is trans.

12. By using a suitable mixed solvent system, a spot of the starting material aldehyde and product mixture can be used to give a difference in R_f values to observe product formation over time. An example system for monitoring this reaction is given here; TLC conditions: (eluent: hexanes/ethyl acetate 5:1); Rf product = 0.75, spot 1 and R_f anthraldehyde = 0.50, spot 2. By monitoring the emergence of spot 2 while spot 1 disappears over time, we can conclude when the reaction has gone to completion.

10. Suzuki-Miyaura: forming C-C bonds

Introduction

The Suzuki-Miyaura cross-coupling reaction is a well-documentated organic chemistry procedure which faciliatates C-C bond formation using through the coupling of a boronic acid and organohalide, usually catalysed by a [Pd]-based catalyst. The reaction has been shown to be universally applicable to wide variety of substrates which require C-C bonds to be formed.[10] By modifying the aryl groups on either reagent, it has been shown that chiral or stereoselective compounds may be afforded[11] with a recent application employing a biologically-based solvent.[12]

The following method (Method 1) has been adapted from J. Chem. Educ. 2013, 90, 11, 1509-1513: An Operationally Simple Aqueous Suzuki–Miyaura Cross-Coupling Reaction for an Undergraduate Organic Chemistry Laboratory. Method 2 is an alternative method which employs the dynamic nature of the equilibrium in this reaction was the addition of excess water to afford the final product.

Method 1/2

Experimental procedure

1. Add 4-bromobenzoic acid (2.5 mmol, 0.5 g) and phenylboronic acid (3.0 mmol, 0.37 g) to R1, equipped with a magnetic stir bar.
2. In a 50 mL beaker, dissolve sodium carbonate (7.5 mmol, 0.80 g) in 15 mL deionised water and stir until dissolved.
3. Using a syringe, add this sodium carbonate solution to R1 via Port 1, stirring vigorously until all reactants are dissolved (~10min).

4. Heat the solution inside of the vessel to 70 °C and add 1.0 mL of the palladium catalyst stock solution to R1 via Port 1 and continue to stir. A water bath can be used for this step.
5. Allow the reaction to stir at 70 °C for at least 30 minutes. The product should precipitate as a white solid. The reaction can be monitored by TLC (EtOAc:PE, 2:1)
6. Allow to cool to RT, then put the monolith into an ice bath.
7. Whilst vigorously stirring, dropwise add 25 mL of 1M HCl *via* a disposable pipette into R1 (Port 1).
8. Once all the HCl is added, stir for 5 minutes.
9. Close Port 1 with a cap equipped with a Luer lock. Unscrew the screw-valve between the chambers just enough for the solution to be pushed from R1 to FR2, apply compressed air/N_2 gas to transfer the reaction mixture to FR2, then close the drill hole by tightening the screw.
10. Isolate the product in FR2 by vacuum filtration. Add 5 mL of water to Port 2 on FR1 to wash the crude product and pull the vacuum to dry the crude product for about 2 minutes.
11. Add 4 mL of 1M HCl and a stirrer bar to Port 2 and heat the stirred solution to 70 °C. A water bath can be used for this step.
12. Slowly add 15-20 mL of EtOH to Port 2 to dissolve the material fully.
13. a) Let the solution cool to room temperature and then place in an ice bath for 15 mins. If the product crystallizes, continue to step 14. If product fails to crystallize, follow step 13 b).
13. b) Heat up the solution back up to 70 °C until full dissolution. When dissolved, take the monolith out of the heating bath. Quickly equip Port 2 with a screw cap with a Luer lock, open the bottom port on FR2 and use compressed air/N_2 gas to filter the hot solution into a glass beaker where the product will crystallize. When the mixture is at room temperature, put inside an ice bath for 20 minutes.
14. Isolate the crystalline product by vacuum filtration - can add a few mL of cold EtOH to wash the product (if necessary).

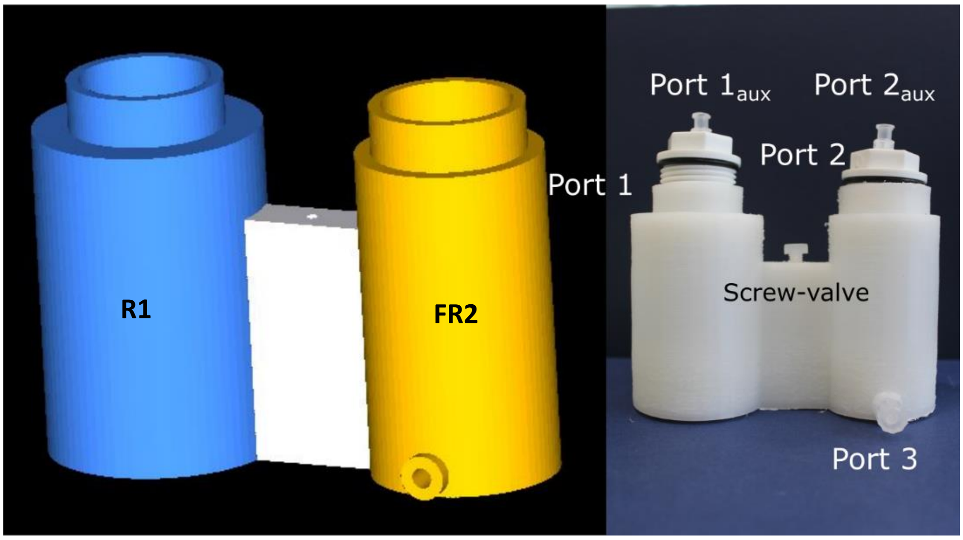

Figure 45: Dual cartridge for the synthesis of the cross-coupled product using Method 1 (left); actual 3D printed model of the cartridge labelled with the ports and valve (right).

Method 2/2

Experimental procedure

1. Add 4-bromobenzoic acid (2.5 mmol, 0.5 g) and phenylboronic acid (3.0 mmol, 0.37 g) to R1, equipped with a magnetic stir bar.
2. In a 50 mL beaker, dissolve sodium carbonate (7.5 mmol, 0.80 g) in 15 mL deionised water and stir until dissolved.
3. Using a syringe, add this sodium carbonate solution to R1 via Port 1, stirring vigorously until all reactants are dissolved (~10min).
4. Heat the solution inside of the vessel to 70 °C and add 1.0 mL of the palladium catalyst stock solution to R1 via Port 1 and continue to stir. A water bath can be used for this step.
5. Allow the reaction to stir at 70 °C for at least 30 minutes. The product should precipitate as a white solid. The reaction can be monitored by TLC (EtOAc:PE, 2:1)
6. Allow to cool to RT, then put the monolith into an ice bath.
7. Whilst vigorously stirring, dropwise add 25 mL of 1 M HCl *via* a disposable pipette into R1 (Port 1).
8. Once all the HCl is added, stir for 5 minutes.
9. Close Port 1 with a cap equipped with a Luer lock. Unscrew the screw-valve between the chambers just enough for the solution to be pushed from R1 to FR2, apply compressed air/N_2 gas to transfer the reaction mixture to FR2, then close the drill hole by tightening the screw.
10. Isolate the product in FR2 by vacuum filtration. Add 5 mL of water to Port 2 on FR1 to wash the crude product and pull the vacuum to dry the crude product for about 2 minutes.
11. Add 5 mL of 1M HCl and a stirrer bar to Port 2 at room temperature.
12. Slowly add 10mL of EtOH to Port 2 to dissolve the material fully.
13. Add 20 mL of deionised water to Port 2 and stir vigourously for 30 min at room temperature. (N.B. ratio of HCl:EtOH:H_2O is 1:2:4)
14. Isolate the crystalline product by vacuum filtration - can add a few mL of cold EtOH to wash the product (if necessary).

Alternative method found by addition of water: in a ratio of 1:2:4 with 1M HCl:EtOH:water, addition of water at room temperature and vigorous stirring for 30 mins will precipate the carboxylic acid product, with a yield of 87% in glassware (after drying overnight) and purity of 99% (negative ESI in acetonitrile) and in reactionware a yield of 75% and a purity of 92%. This presents a viable alternative route to the product since the long-chain (13 carbons) carboxylic acid is highly insoluble in water and therefore will precipitate upon addition of excess water due to the fluctuating equilibrium taking place. This route requires no understanding of recrystalisation and is much safer due to not requiring the use of boiling solvent (EtOH) which is needed in the standard route to gain pure product. However, if one of the teaching objectives was to understand how recrystalisation works, then this method could be used subsequently to addition of water and drying to result in crystals which have a higher purity

and could then be used to obtain a powder x-ray diffraction (XRD) measurement and compare to the known structure, further exposing students to the variety of analytical techniques available to gain chemical and physical data relating to the compound. Further information about this alternative route including NMR spectra is available in the Supporting Information.

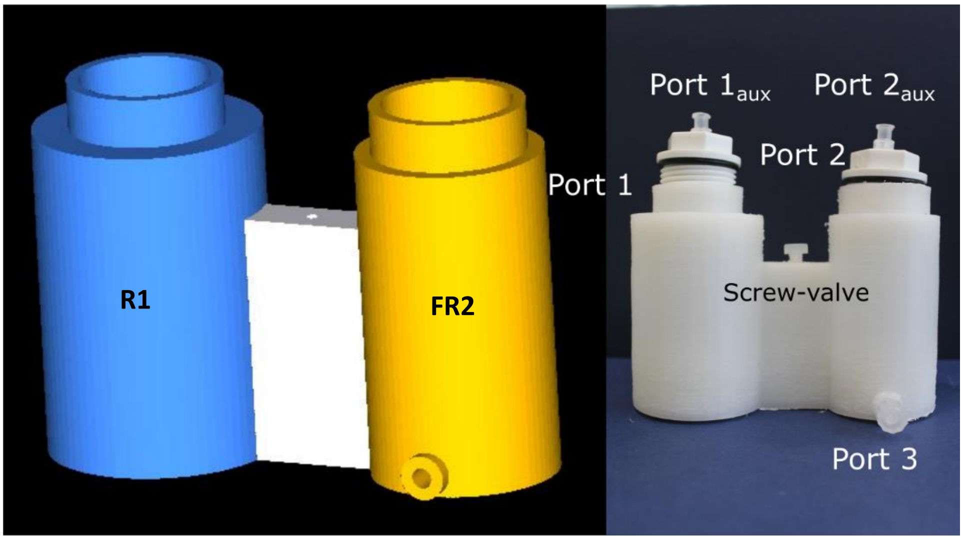

Figure 46: Dual cartridge for the synthesis of the cross-coupled product using Method 2 (left); actual 3D printed cartridge labelled with the ports and valve (right).

Pre- and post-lab questions:

1. Explain the role of [Pd] in this reaction, with reference to the observed rate of reaction.
2. Explain why, when monitoring the reaction by TLC, a solvent mixture of EtOAc and diethyl ether was chosen?
3. To what extent is this reaction dependant on temperature?
4. Why is 1 M HCl added in step 7?
5. What are the expected side products of this reaction and what is one way the reaction may be driven towards the desired product?
6. Describe fully (with curly-arrows) the mechanism of the cross-coupling reaction, including the role of the [Pd].
7. Using knowledge of the 12 principles of green chemistry, how could the yield of this reaction be optimised?

Guideline Answers:

1. The role of the [Pd] catalyst is to increase the rate of reaction by facilitating the formation of reactive transmetallated Pd(II) ligand complex. Kinetic studies showed that reductive elimination obeys first order kinetics, hence the rate and reaction is dependent only on the concentration of the post-transmetallation Pd-complex.
2. The mixture of solvents was chosen in order to allow for dissolution of impurities in the reaction mixture and provide observable separation between the TLC spots for the starting materials, product and co-spot for the reaction mixture. The volatility of this solvent mixture allows for the solvent front to easily travel up the TLC plate and observe a measurable difference for calculating the R_f value.

3. Temperature has little to no effect on the reaction as the large turnover number (TON) of the [Pd] catalyst means it has the most significant effect on reaction rate as this is directly proportional to the concentration of the [Pd] catalyst. However a recent study suggests that reactivity is reduced at lower temperatures when employing aryl iodides as a coupling agent (*Organometallics* 2018, 37, 11, 1745–1750).
4. The highly concentrated acid is added in step 7 in order to quench any remaining base in the reaction mixture and ensure the equilibrium shifts to towards complete product formation.
5. Beta-hydride elimination competes with reductive elimination, which affords a side product that greatly reduces the yield. This is observed with reactants that have beta-hydrogens, most commonly alkyl substrates, but sometimes can be avoided by using Ni catalysts or ligands with larger bite angles.

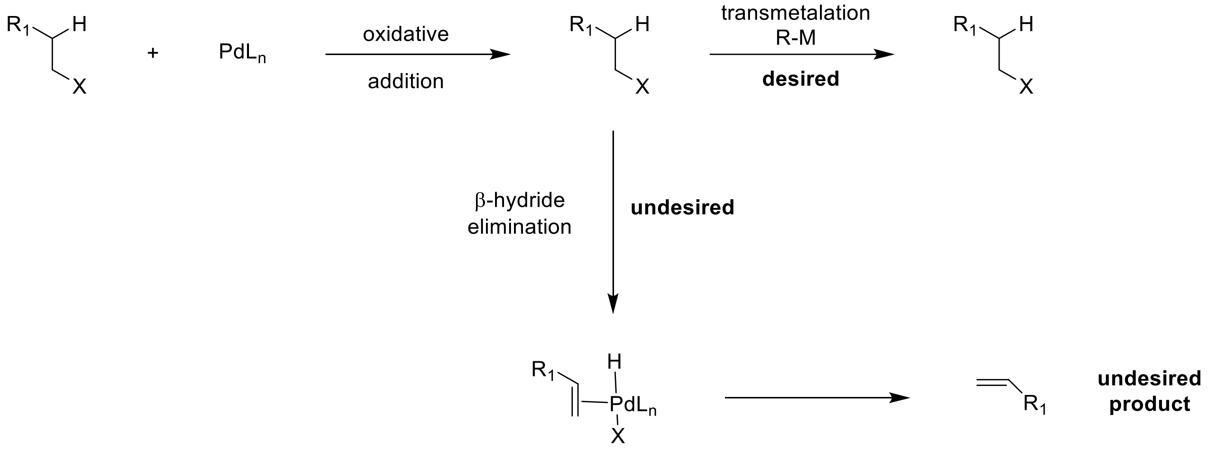

6. The catalytic cycle for the cross-coupling is shown below:

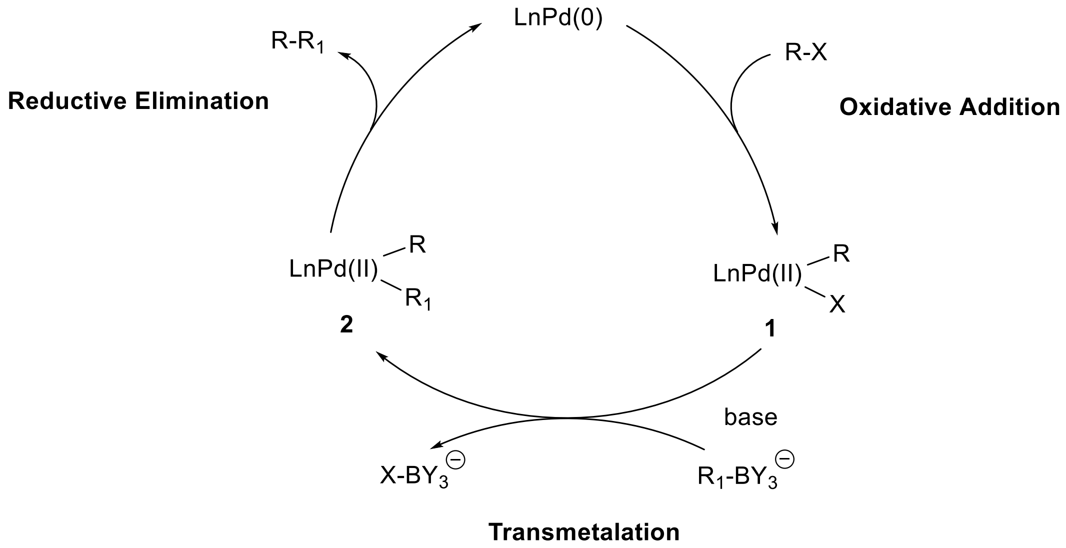

7. The yield may be increased by using more reactive aryl iodides, which perform better than similar aryl bromides in this reaction. The use strong bases (NaOH, TlOH, and NaOMe) perform optimally in THF or H_2O solvent systems, which are considered greener solvent alternatives to the diethyl

ether for example. Also, Thallium bases such as TlOH, and its derivatives Tl_2CO_3 and TlOEt, enable cross coupling between alkyl boranes and alkyl halides. It also allows this reaction to proceed at lower temperatures (20 °C from the usual 50 to 80 °C). Unfortunately, these bases are air and light sensitive, and exceptionally toxic, but are still widely used. Use of Ni in catalysis is favored due to its abundance, and thus low cost. Organoboranes are nontoxic and stable to extreme heating and exposure to oxygen or water. Consequently, these reagents can be easily used at benchtop, and do not require special equipment or techniques, such as gloveboxes or air-sensitive and dry technique. This reaction has a high atom economy seeing that the byproducts are typically salts and water soluble borane byproducts. The lack of interfering byproducts allows for one-pot syntheses to further reduce waste and increase reaction efficiency. These attractive features of organoborane reagents increases its utility and synthetic convenience, and makes Suzuki coupling optimal for large scale and industry synthesis.

11. Synthesis of sulfanilamide: preparing drug molecules

Reference: Williamson, K.L. Macroscale and Microscale Organic Experiments; Heath: Lexington, MA, 1994; pp 541-557]

Introduction

The project is comprised of three parts:

- Process selection
- Practical experiment
- Critical evaluation

Part 1 – process selection, reactor design

In this project, you will be tasked to compare two chemical processes and perform a selection by looking closer at the reaction conditions and unit operations and evaluating which of the two is more efficient. The next step will involve process analysis and the dissection of the chemical process into its respective unit operations. Defining "Unit Operations" (UnOps) is a central skill required of chemical engineers. Chemical engineers often use PFDs or Process Flow Diagrams to depict the details of unit operations. You will be tasked to draw a PFD and, using CAD software designed for chemists, computationally design reactionware reactor systems in which the process could (theoretically) be carried out. This part be turned into for marking.

Part 2 – practical experiment

By this point you will have received feedback on your selected process, PFD as well as the turned-in reactionware model. You will be given the correct synthesis details and it's PFD together with a printed reactionware model. Accompanied there are pre-lab questions that should be answered before the beginning of the practical experiment. In the practical part, carry out the procedure for the synthesis of Sulfanilamide in reactionware according to the procedure given and try to associate each operation to the one depicded in the PFD. After turning in your product

Sulfonilamide powder, answer the post-lab questions and turn your answers along with your answers to the pre-lab questions for marking.

Pre-lab questions

1. List the principles of green chemistry.
2. What do you understand to be a ‘unit operation’?
3. Given your understanding of what a PFD should depict, how might you improve your PFD to reflect better the processes involved in this synthesis?
4. Given a list of four solvents: water, methanol, 1,4-dioxane and THF, rank these in order of “greenness” taking into account the 12 principles of green chemistry and explain clearly the reasons for your chosen order.
5. In an industrial batch process, there are often various key factors at play, which all contribute to the economic cost for the manufacturer and ultimately the consumer of the product being made. Given this procedure for sulfinilamide, what are the key factors and how could they be altered to make the process more cost-effective?
6. Given two routes for this synthesis: a) route involves high-pressure reactors and high-temperature, yet provides high yield and purity or b) route involves lower temperature and yield, less harmful solvent and faster reaction time – which would you choose and why?
7. Explain two spectroscopic methods you could use to determine whether or not you have made the desired product and what characteristic features you would look for in each technique.
8. In industry such reactions as these are done on kilogram-scale, often in large heated metal vats or using large glassware appartus. However, here we use 3D-printed polypropylene reactionware to synthesise the desired product. In your own words, provide a brief description about how you think reactionware compares to these alternative setups.
9. These 3D-printed reactionware monoliths use polypropylene as the material for manufacture, as it is relatively cheap and highly chemically inert. With reasons, suggest what other factors are important when deciding the material to use for these 3D-printed vessels?
10. If you were given this experiment again, what would you change to improve the learning outcomes and/or your experiences with using the ChemSCAD software, following the worked examples and carrying out the synthesis in reactionware?

Guideline Answers:

1. Students should list the 12 principles as follows, with brief description of each principle, which can include an example (from Sigma Aldrich):

- Prevention: It is better to prevent waste than to treat or clean up waste after it has been created.
- Atom Economy: Synthetic methods should be designed to maximize the incorporation of all materials used in the process into the final product.
- Less Hazardous Chemical Syntheses: Wherever practicable, synthetic methods should be designed to use and generate substances that possess little or no toxicity to human health and the environment.

- Designing Safer Chemicals: Chemical products should be designed to affect their desired function while minimizing their toxicity.
- Safer Solvents and Auxiliaries: The use of auxiliary substances (e.g., solvents, separation agents, etc.) should be made unnecessary wherever possible and innocuous when used.
- Design for Energy Efficiency: Energy requirements of chemical processes should be recognized for their environmental and economic impacts and should be minimized. If possible, synthetic methods should be conducted at ambient temperature and pressure.
- Use of Renewable Feedstocks: A raw material or feedstock should be renewable rather than depleting whenever technically and economically practicable.
- Reduce Derivatives: Unnecessary derivatization (use of blocking groups, protection/ deprotection, temporary modification of physical/chemical processes) should be minimized or avoided if possible, because such steps require additional reagents and can generate waste.
- Catalysis: Catalytic reagents (as selective as possible) are superior to stoichiometric reagents.
- Design for Degradation: Chemical products should be designed so that at the end of their function they break down into innocuous degradation products and do not persist in the environment.
- Real-time analysis for Pollution Prevention: Analytical methodologies need to be further developed to allow for real-time, in-process monitoring and control prior to the formation of hazardous substances.
- Inherently Safer Chemistry for Accident Prevention: Substances and the form of a substance used in a chemical process should be chosen to minimize the potential for chemical accidents, including releases, explosions, and fires.

2. Reaction mechanisms below for each step as guidance.

Step 1: Formation of Acetanilide

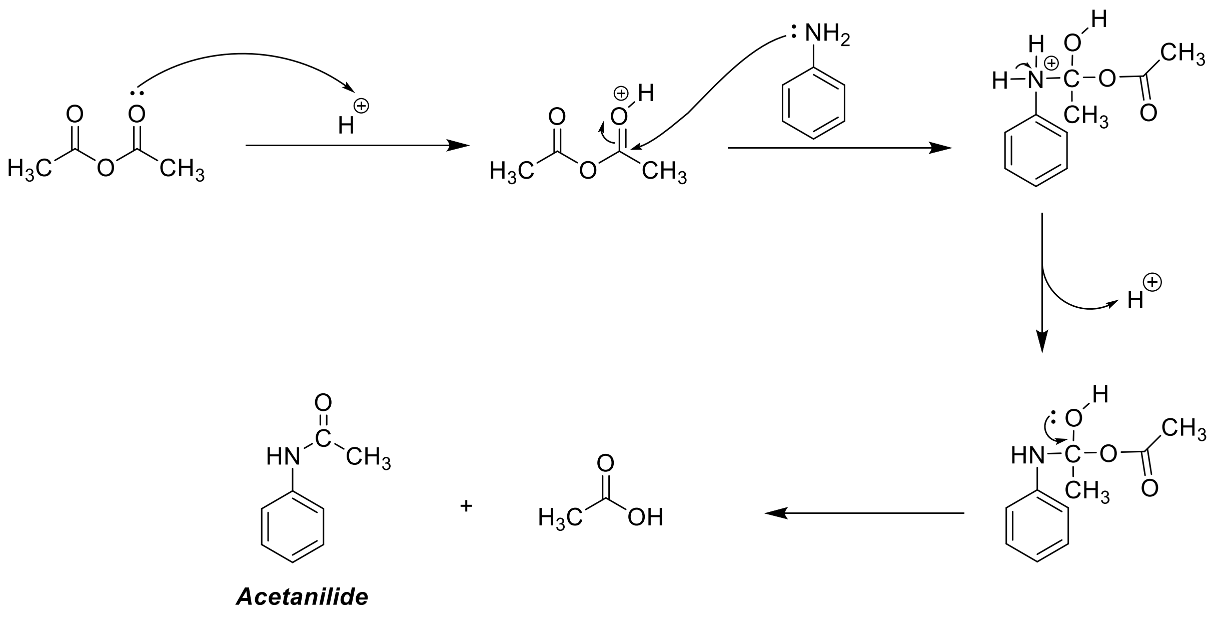

Step 2: Dimerisation of Chlorosulfonic acid (electrophile formation)

Chlorosulfonic acid reacts with itself to form a “dimeric” chlorosulfonic anhydride. This anhydride is the electrophilic reagent in the electrophilic aromatic substitution reaction with acetanilide.

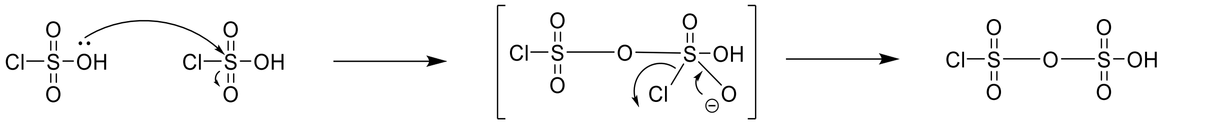

Chlorosulfonic anhydride

Step 3: Formation of p-acetamidobenzenesulfonylchloride via EAS

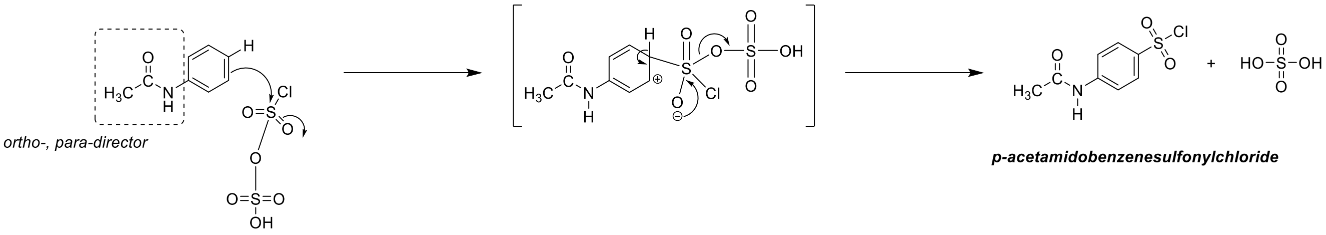

Step 4: Formation of p-acetamidobenzenesulfonamide

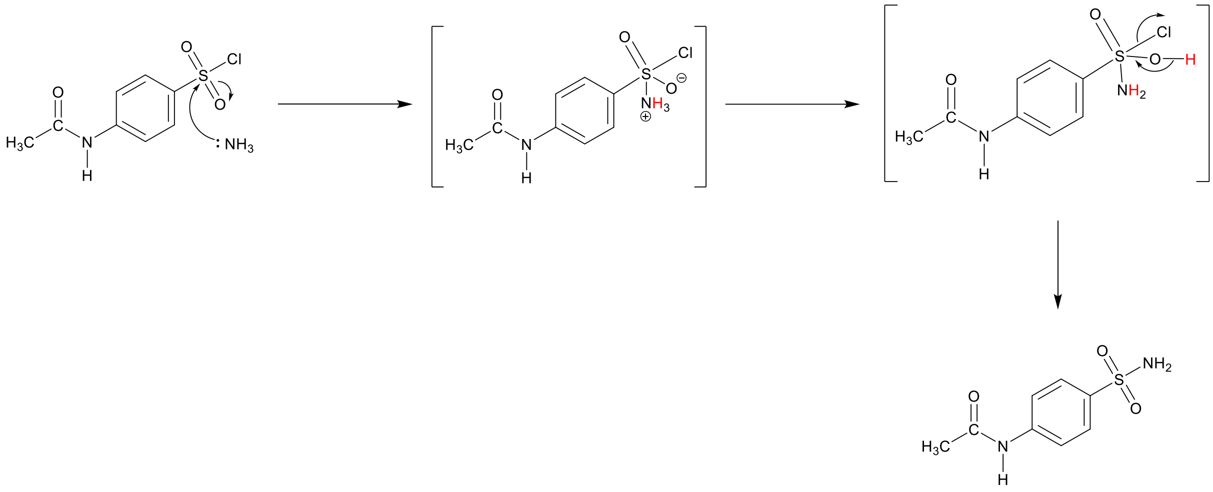

p-acetamidobenzenesulfonamide

Step 5: Amide hydrolysis of p-acetamidobenzenesulfonamide to form sulfanilamide

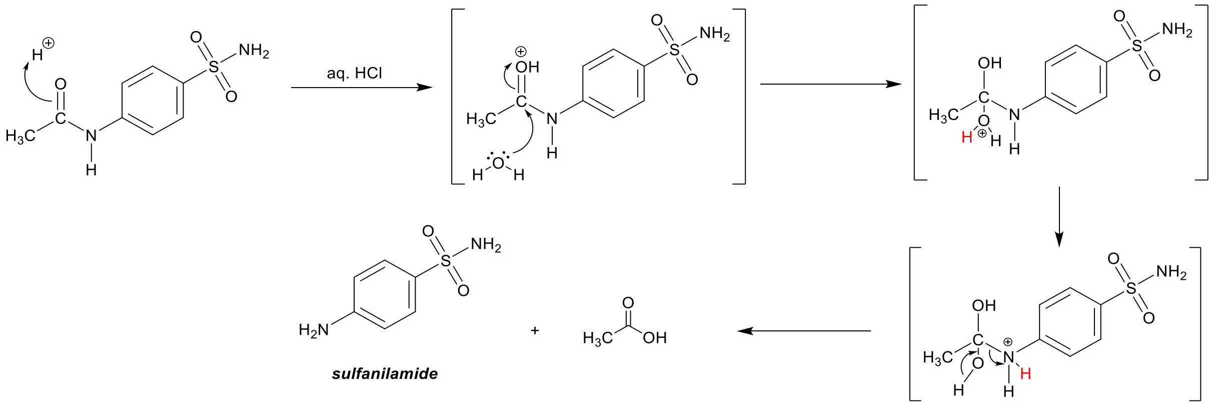

3. Alternatives to MW heating can be suggested i.e. oil bath at high temperature with stirring, use of catalyst (solid or liquid) to lower activation barrier, mechanochemical methods (e.g. ball mill). Logical reasons given, such as heating in oil bath gives good temperature distribution. Mechanochemical methods can be faster due to physical aggitation with the ball.

4. An answer to this effect will be acceptable: In chemical engineering and related fields, a unit operation is a basic step in a process. Unit operations involve a physical change or chemical transformation such as separation, crystallization, evaporation, filtration, polymerisation, isomerisation, and other reactions.

5. Suggestions for improving the PFD may include adding more detail for the work-up steps and combining some steps to increase efficiency of the procedure. A clear understanding of the unit operations must be shown and an appreciation of how these operations map to the processes being performed in reactionware.

6. The ranking of solvents in terms of environmental safety is as follows:

Water < Methanol < 1,4-dioxane < THF (water most safe, THF least safe)

Reasons for this includes risk of flammability, toxicity and irritability of solvent. As well as tendency to perform side reactions. Cost is also a factor to consider, water is plentiful compared to THF which is relatively expensive, for example. Other valid reasons may be provided by students building on their understanding of the 12 principles of green chemistry outlined above.

7. In industry, the key to any batch process is the recovery of materials and regulatory processes involved with producing any drug or drug-like molecule. Therefore, important factors affecting economics of this process involve scale of the synthesis, recovery of solvent and starting materials, recyclability of reagents and electricity costs to run the process plant. In reationware these processes are telescoped so costs are lower in terms of solvent use and material cost (£1000 / 50 kg of PP). Concern in Reactionware is recyclability of the monoliths and recovery of lost product during transfer steps for example.

8. Students may suggest either route as viable if valid reasons are given. For route A, a suggestion could be the procedural cost is lower due to reaction time being shorter and use of high pressures means more material may be produced overall, leading to cost-effectiveness for this procedure. However, high temperature coupled with high pressure presents major safety risks in terms of potential explosions during manufacturing. Less of an issue in reactionware given smaller scale but still a considerable risk. For route B, lower temperature and less harmful solvent provides a greener alternative (12 principles) but sacrifices yield and purity of the final product. In reactionware, route B is preferred as the use of microwave heating allows for similar benefits to using a high-pressure reactor in route A. Students could suggest a hybrid method which combines aspects of route A and B, which could feed into their PFD design for the synthesis procedure, thus demonstrating understanding and critical evaluation of the pros and cons for each route.

9. NMR is a key method to identify peaks of interest. Best to do is carbon-13 proton decoupled, proton NMR and nitrogen-15 proton decoupled NMR. Also, IR provides a finger print for the key functional groups in the molecule (i.e. amide bond). A melting point analysis may be used to determine the crude purity of the product too.

10. Students can suggest benefits of reactionware such as: easy to handle, easy to operate following procedure given, safer since operations are contained, easy to map operations performed to reactionware, overall cost much lower than glassware procedure. Cons of reactionware could be: bespoke for a given synthesis, may not adapt well to more complex processes such as total synthesis,

time for printing can lead to lots of waiting around (less time to analyse, repeat experiments), more difficult to parallise a synthesis in reactionware, solvent compatibility is an issue due to PP being used as material of reactionware, heat retention in reactionware is poor (heating needs to be 20 °C higher than target temp in Reactionware), recyclability of reactionware hasn't been assessed, not clear if reactions may be repeated in same monolith.

11. In addition to the lowest cost and high chemical inertness of PP, students can suggest the temperature resistance of PP as a benefit, but als a negative i.e can't heat above 120 °C without swelling of reactor taking place. Ability to design and print complex geometries and topologies not possible in glassware to simplify complex procedures involving multiple operations. Any other viable reasons may be suggested in addition to these.

12. Students can suggest ideas for improvements to the ILOs such as more time spent in *ChemSCAD* understanding the software before designing their monoliths. In the worked examples, suggestions around providing more extensive PFD examples to aid learning and understanding, and in the synthesis procedure, suggestions about performing reaction at different scale, doing 3D printing themselves to learn how this works to build desired topology can all be suggested ideas to improve. Also suggestiosn for improved feedback can be given, such as discussion about translating procedure to unit operations to PFD to *ChemSCAD* design.

Experimental procedure

The following experimental procedures are performed in the monolith shown below. The figure details the 3D model of the monolith (left) and a labelled actual 3D printed model of the monolith (right). All procedures refer to the 3D printed model as labelled.

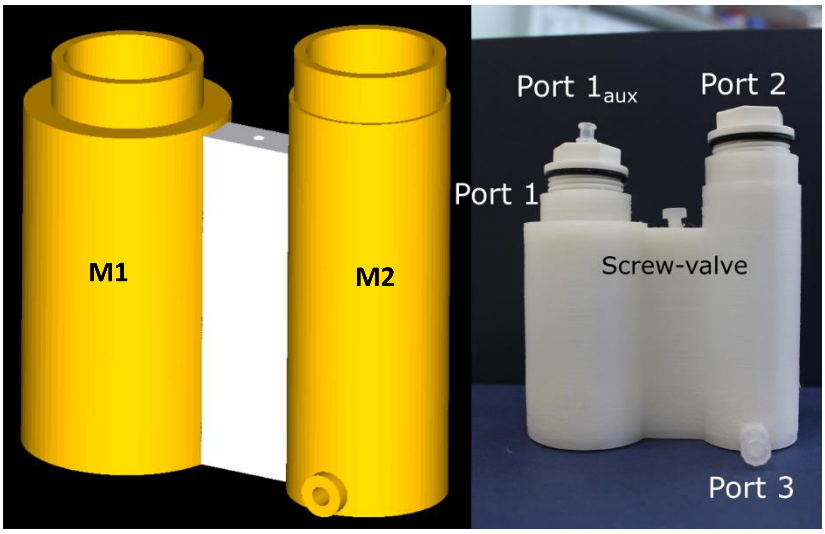

Figure 47: Dual cartridge for the synthesis of sulfanilamide as a 3D model (left); actual 3D printed monolith labelled with ports and valve (right).

Acetanilide:

Note: the first synthesis step uses aniline, which is hygroscopic. If a fresh aniline bottle is used, the solution to be prepared will be colorless and can be prepared directly in **M1**. Otherwise it will be brown. If that's the case, the coloration can be removed by first preparing the solution in glassware and filtering it through a pad of activated carbon (charcoal) on a Buchner funnel. Acetic anhydride, which also starts undergoing degradation upon the opening of a new reagent bottle, also goes off, thus, the yields for the first step of the synthesis will vary from student to student. The amount of materials need to be scaled accordingly for further steps.

1) Dissolve of aniline (2 g, 21.5 mmol) in water (60 mL) and concentrated hydrochlorid acid (2 mL) in **M1**. Use a medium-sized stir bar and a magnetic hotplate.
2) Prepare R_1: a syringe of acetic anhydride (2.4 mL).
3) Prepare R_2: a syringe of 2M aqueous sodium acetate (2 g in 12 mL).
4) With stirring, at the same time add both R_1 and R_2 into **M1**. The addition of both reagent requires to be simultaneous as this maximises the yield due to the acetic anhydride starting to react with the aqueous medium.
5) Stir the mixture for approximately 5 minutes.
6) Load ice into a oil bath and submerge the reactionware monolith in it. Cool for approximately 30 minutes. Sodium chloride can be added around the monolith to reach lower temperatures (optional).
7) Attach a vacuum line fitted with a solvent trap to P_2 to filter the white crystalline powder product.
8) Close both P_1 and P_2, use PTFE tape to ensure sealed conditions.

9) Dry overnight before the next step. Attach a vacuum line to P_{1aux}.
10) Characterize the acetanilide product. Note appearance, yield, use common NMR techniques (^1H and ^{13}C).

p-Acetamidobenzenesulfonyl chloride:

Note: chlorosulfonic acid, being a superacid, is **extremely reactive** when exposed even to slight amounts of moisture, expelling harmful HCl fumes as soon as the reagent bottle is opened. Hence, **extra care** should be taken when handling this reagent and it is imperative that the acetanilide is dried, otherwise, unwanted by-products and H_2SO_4 will dominate the resulting mixture turning it into a black sludge.

11) Prepare an oil bath with silicone oil by heating it up to 120 °C.
12) With stirring, add chlorosulfonic acid (5 mL, 0.75 mol) dropwise to **M1** containing the dried acetanilide (2 g, 15 mmol).
13) Allow the mixture to react for 10 minutes, before submerging the reactionware monolith into the oil bath. Make sure that P_4 is closed. Meanwhile, prepare a beaker of cold of deionized water (24 mL) by submerging the beaker in an ice bath.
14) Allow the mixture to react a further 10 minutes before raising it out of the oil bath. Wipe excess oil off sides of monolith.
15) Add a medium-sized stir bar and the cold water to **M2**.
16) With stirring, perform a pressure transfer of the reaction mixture from **M1** to **M2** keeping **M2** at the centre of the magnetic field of the stir plate. Refer to the material transfer section of the SI.
17) Keep stirring until an even suspension of white solid is obtained.
18) Perform a vaccum filtration by applying a vacuum line equipped with a solvent trap to P_4 to collect the acetamidobenzenesulfonyl chloride as a white chalky solid. The solid can be washed with a small amount of water to remove residual impurities.

p-Acetamidobenzenesulfonamide:

19) Prepare R_3: 25% ammonium hydroxide (9 mL) in water (9 mL).
20) Prepare an oil bath by heating it up to 120 °C.
21) With stirring, add R_3 into **M2**.
22) Submerge monolith into oil bath and heat with stirring for 30 minutes. Ensure there is no solid above the solvent from in the reactor.
23) Lift the monolith from the oil bath and wipe off the excess oil.
24) Cool the monolith in an ice bath for at least 30 minutes.
25) Perform a vacuum-filtration through P_4 to collect the product as a moist white powder.

Sulfanilamide:

26) Prepare $R_{4:}$ concentrated HCl (3 mL) with of water (6 mL).
27) Stir the mixture until an even suspension is formed.
28) Place the monolith into a microwave oven and irradiate at a low microwave setting for at least an hour with all ports fully closed apart from P_1 cap being half-way screwed in. Note: We achieved a mostly reacted mixture when irradiating for 2 hours at a 20% power setting with a Daewoo (KOR-6A0R, 800 W, 230 V, ~50Hz) domestic microwave oven.

29) Allow the solution to cool to room temperature. Upon cooling no solid should deposit. If a solid forms, consider irradiating for longer / higher power setting.
30) Submerge monolith in an ice bath and let the temperature to equilibrate.
31) Prepare R_5: 0.94 M aqueous Na_2CO_3 (15 mL).
32) With stirring, add R_5 to **slowly** to **M2**. Note: using sodium carbonate produces much foam. Alternatively, sodium hydrogen carbonate can be used, but at the cost of total reaction mixture volume. A larger volume of sodium hydrogen carbonate will be needed thus a larger version of **M2** would need to be designed using ChemSCAD.
33) The product sulfinamide will precipitate out as the pH reaches 7. Carry out a vacuum filtration by applying vacuum to P_4.
34) Dry the product and characterize.

References

1 Kelly, A. M., Chen, P.-J., Klubnick, J., Blair, D. J. & Burke, M. D. A Mild Method for Making MIDA Boronates. *Organic Letters* **22**, 9408-9414, doi:10.1021/acs.orglett.0c02449 (2020).

2 Jaworek, C. & Iacobucci, S. Wittig Reaction: The Synthesis of trans-9-(2-Phenylethenyl)anthracene Revisited. *Journal of Chemical Education* **79**, 111, doi:10.1021/ed079p111 (2002).

3 Williamson, K. L. Synthesis 2: Synthesis of Sulfanilamide from Aniline. *Macroscale and microscale organic experiments*, 541-557 (1994).

4 Gujral, S. S., Khatri, S., Riyal, P. & Gahlot, V.

5 Kelly, A. M., Chen, P.-J., Klubnick, J., Blair, D. J. & Burke, M. D. A Mild Method for Making MIDA Boronates. *Organic Letters*, doi:10.1021/acs.orglett.0c02449 (2020).

6 Waterman, K. C. *et al.* Hydrolysis in Pharmaceutical Formulations. *Pharmaceutical Development and Technology* **7**, 113-146, doi:10.1081/PDT-120003494 (2002).

7 Brown, D. G. & Boström, J. Analysis of Past and Present Synthetic Methodologies on Medicinal Chemistry: Where Have All the New Reactions Gone? *Journal of Medicinal Chemistry* **59**, 4443-4458, doi:10.1021/acs.jmedchem.5b01409 (2016).

8 Pinho e Melo, Teresa M. V. D. *et al.* Novel Asymmetric Wittig Reaction: Synthesis of Chiral Allenic Esters. *European Journal of Organic Chemistry* **2004**, 4830-4839, doi:https://doi.org/10.1002/ejoc.200400413 (2004).

9 Maryanoff, B. E. & Reitz, A. B. The Wittig olefination reaction and modifications involving phosphoryl-stabilized carbanions. Stereochemistry, mechanism, and selected synthetic aspects. *Chemical Reviews* **89**, 863-927, doi:10.1021/cr00094a007 (1989).

10 Hussain, I., Capricho, J. & Yawer, M. A. Synthesis of Biaryls via Ligand-Free Suzuki–Miyaura Cross-Coupling Reactions: A Review of Homogeneous and Heterogeneous Catalytic Developments. *Advanced Synthesis & Catalysis* **358**, 3320-3349, doi:https://doi.org/10.1002/adsc.201600354 (2016).

11 Molander, G. A. & Felix, L. A. Stereoselective Suzuki–Miyaura Cross-Coupling Reactions of Potassium Alkenyltrifluoroborates with Alkenyl Bromides. *The Journal of Organic Chemistry* **70**, 3950-3956, doi:10.1021/jo050286w (2005).

12 Wilson, K. L., Murray, J., Jamieson, C. & Watson, A. J. B. Cyrene as a bio-based solvent for HATU mediated amide coupling. *Organic & Biomolecular Chemistry* **16**, 2851-2854, doi:10.1039/C8OB00653A (2018).

13 Hou W., Bubliauskas, A., Kitson, J. P., Francoia J. P., Powel-Daview H., Guitierrez Parrilla J. M., Frei P., Manzano S. J., & Cronin, L. Automatic Generation of 3D-Printed Reactionware for Chemical Synthesis Digitization using ChemSCAD. *Central Science* **7**, (2) 212-218, doi.org/10.1021/acscentsci.0c01354.

14 Zalesskiy, S.S., Kitson, P.J., Frei, P. et al. 3D designed and printed chemical generators for on demand reagent synthesis. Nat Commun 10, 5496 (2019). https://doi.org/10.1038/s41467-019-13328-6

Appendix

Experiment 5 – Carboxylic Acid and Ester Functional Groups

Introduction

In this experiment the aim is to identify an unknown ester (it will be either a **methyl or ethyl** ester) by hydrolysing it, isolating the acid produced, and identifying it from its melting point. Boiling points are hard to measure accurately, and are more prone to variation (e.g. from atmospheric pressure variation) than melting points, so the latter are used for identification.

Esters can be hydrolysed under *alkaline conditions* to give alcohols and the carboxylate salts. In this experiment, the unknown ester will be hydrolysed using hot aqueous alkali, to produce the sodium salt of the carboxylic acid, and either **methanol or ethanol** (depending on whether an ethyl ester or an ethyl ester was provided). Acidification of this reaction mixture will give the (water insoluble) carboxylic acid. This acid is collected and its melting point is measured, thusly providing its identification. From this, the identity of the unknown ester can be established.

Safety considerations

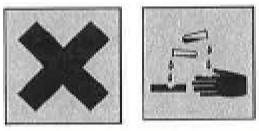

Although the quantities you are handling are small, remember that all **carboxylic acids** and **esters** are flammable, corrosive/irritant, and toxic. Hot **5 M sodium hydroxide** and **5 M hydrochloric acid** are extremely corrosive and must be handled with great care.

Location of chemicals

The **ester** is in your kit. The 5 M **hydrochloric acid** and 5 M **sodium hydroxide** solutions are kept above the benches. The balances are kept on the bench. **Ethanol** is kept above the benches. **Purified water** is found in plastic squeeze bottles above the benches.

Figure 48: Page 1/3 of a scanned laboratory manual detailing the ester hydrolysis experiment.

Hydrolysis and identification of the ester

Firstly record the **number** of the unknown ester that you have been supplied with. Note that Ester 4 is a **methyl** ester, while the rest are **ethyl** esters. If you have been provided with a solid ester, weight accurately about 1.00 g into a 50 mL round-bottomed flask (see **Appendices 1-2** and pre-lab online **simulations** for weighing). If your ester is a liquid, use a Pasteur pipette to transfer 20-25 drops of the ester into a 50 mL round-bottomed flask (see **Appendix 8**). Record accurately the **mass** of the ester used.

Add 15 mL aqueous sodium hydroxide solution (5 M) to the flask containing your ester, followed by 3 mL ethanol (see **Appendix 8** for pipette use). *Note that aqueous sodium hydroxide solution is corrosive and should be handled with care.* Now add two **boiling stones** to the flask to prevent bumping during reflux. Record any changes.

Set up reflux apparatus using a heating mantle and the 50 mL round-bottomed flask containing your mixture (see **Appendix 11**). Two clamps are required, one on the neck of the flask, and one around the middle of the Findenser (see **Appendix**). Do not over-tighten the clamps. If you are still unsure how to set up the apparatus correctly, ask a demonstrator. Heat the flask on full heat until the solution just begins to boil, then reduce the heat and continue to heat the flask, maintaining vigorous reflux for approx. 25 mins.

Now switch off the heating mantle, and using the clamp, lift the flask out of the heating mantle (Findenser still attached), allowing the apparatus to cool before removing the Findenser. When the flask is cool enough to handle comfortably, slowly add 15 mL aqueous hydrochloric acid (5 M). *Note that aqueous hydrochloric acid is corrosive, and should be handled with care.* Swirl the flask to help dissipate the heat produced by addition of the acid. Use a glass rod to spot a small amount of the mixture onto universal indicator paper, to check if the solution is acidic. If necessary, add a little more aqueous hydrochloric acid (5 M) to ensure that the pH of the mixture is approx. 1.

Once the acid has been added and the flask left to cool, the resulting carboxylic acid will precipitate out of solution. If crystallisation does not occur, cool

Figure 49: Page 2/3 of a scanned laboratory manual detailing the ester hydrolysis experiment.

the flask in an ice-bath (see **Appendix 10**) and carefully scratch the side of the flask using a glass rod.

When crystallisation is complete, collect the crystals of carboxylic acid under vacuum using a Buchner funnel and flask (see **Appendix 5** and pre-lab online **simulations**). Wash the crystals in the flask with a little de-ionised water, and dry them for 5 mins under vacuum.

Record the mass of carboxylic acid obtained (see **Appendices 1-2** and pre-lab online **simulations**) and the melting point (this should be a range, see **Appendix 7** and pre-lab online **simulations**).

Do not leave the laboratory until the demonstrator has checked your work area is clean and tidy and you have submitted your labelled product samples.

Remember to complete and submit your online auto-graded report within 24 h.

Melting Point Ranges of Selected Carboxylic Acids	
Carboxylic Acid	***Melting Point Range (°C)***
Benzoic	122-123
2-Hydroxybenzoic	158-160
3-Hydroxybenzoic	201-203
4-Hydroxybenzoic	215-217
2-Methylbenzoic	103-105
3-Methylbenzoic	108-110
4-Methylbenzoic	180-182
2-Methoxybenzoic	98-100
3-Methoxybenzoic	106-108
4-Methoxybenzoic	182-185

Figure 50: Page 3/3 of a scanned laboratory manual detailing the ester hydrolysis experiment.
